# Electrochemical Benzylic C(sp^3^)–H Direct Amidation

**DOI:** 10.1021/acs.orglett.3c04012

**Published:** 2024-01-16

**Authors:** Anthony Choi, Oliver H. Goodrich, Alexander P. Atkins, Matthew D. Edwards, David Tiemessen, Michael W. George, Alastair J. J. Lennox

**Affiliations:** † School of Chemistry, University of Bristol, Bristol BS8 1TS, U.K.; ‡ School of Chemistry, University of Nottingham, Nottingham NG7 2RD, U.K.

## Abstract

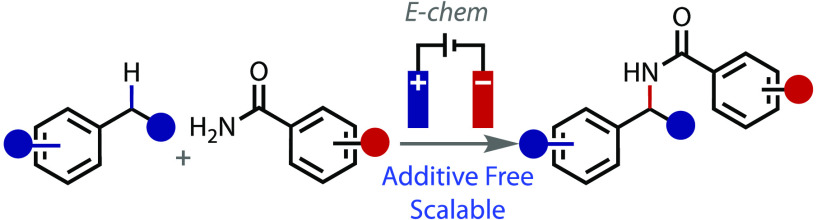

Amide bonds are ubiquitous and found in a myriad of functional molecules. Although formed in a reliable and robust fashion, alternative amide bond disconnections provide flexibility and synthetic control. Herein we describe an electrochemical method to form the non-amide C–N bond from direct benzylic C(sp^3^)–H amidation. Our approach is applied toward the synthesis of secondary amides by coupling secondary benzylic substrates with substituted primary benzamides. The reaction has been scaled up to a multigram scale in flow.

Amides are ubiquitous and important motifs found in many drugs and natural products.^1,2^ The unique hydrogen-bonding properties, polarity, conformational rigidity, and high structural stability of the amide bond provide the functional rationale for amide incorporation into myriad biologically active compounds.^2^ While traditional methods that form amide bonds readily enable their preparation in a robust and reliable fashion^3,4^ (Figure 1A), there is a continuing necessity to develop a range of diverse synthetic methods to prepare these motifs. In particular, a departure from amide bond-forming strategies provides enhanced flexibility to retrosynthetic planning and potential benefits for drug discovery through target derivatization.^4−6^

**Figure 1:**
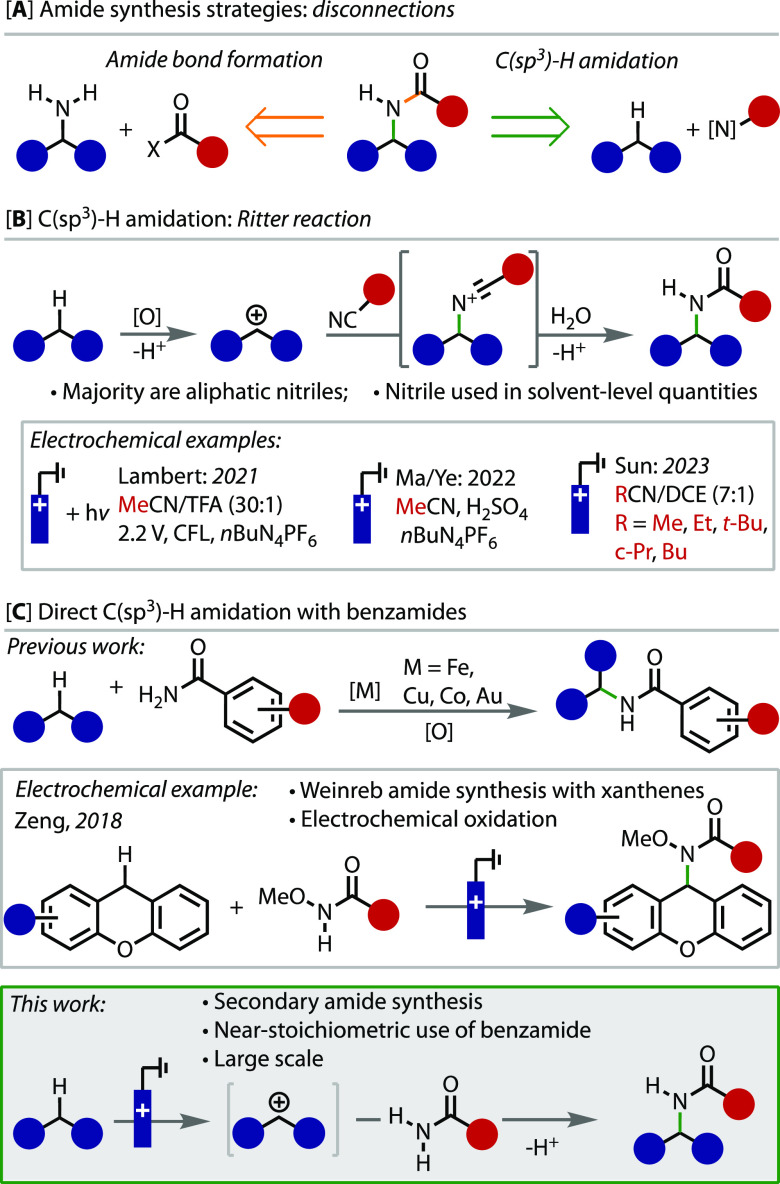
(A) Amide bond-forming strategies. (B) Well-established Ritter reaction with nitriles and electrochemical examples. (C) Direct C(sp^3^)–H amidation with benzamides as coupling partners.

An alternative disconnection for the preparation of secondary amides is the non-amide N–C bond. This bond formation is especially appealing when accessed through a C(sp^3^)–H amidation because of the simplicity and frequency of suitable C(sp^3^)–H bond-containing coupling partners (Figure 1A). Central to this strategy is the Ritter reaction,^7^ in which a nitrile group quenches a carbocation to form the C–N bond, and further hydrolysis yields a secondary amide (Figure 1B). This approach is particularly relevant and appealing when the cation derives from the oxidation and deprotonation of a C(sp^3^)–H bond.^8,9^

Electrochemistry provides a more sustainable and selective method for conducting oxidation reactions^10^ and has been shown to enable a plethora of new reactivity and selectivity.^11−13^ Indeed, anodic oxidation has successfully been applied in Ritter-type C–H amidation reactions (Figure 1B).^14−16^ However, a fundamental limitation with this approach is the availability and quantity needed for the nitrile coupling partner, as it is generally used in solvent-level quantities. This is reflected in the vast majority of cases featuring acetonitrile as the nitrile source, which proceeds to give acetamides.

The use of primary amides as nucleophilic coupling partners in oxidative benzylic C(sp^3^)–H functionalization reactions is an alternative strategy. This transformation has been successfully realized with the use of stoichiometric oxidants in combination with metal catalysts (Figure 1C).^17−20^ Although these methods are versatile and can be applied to a wide variety of substrates, the use of metal catalysts and stoichiometric oxidants is costly and generates waste, which are notable disadvantages. The use of electrochemical oxidation has been shown to be an effective method for the formation of Weinreb amides of xanthenes (Figure 1C).^21^ In a related contribution, Xu and co-workers demonstrated that sulfonamides are suitable nucleophiles under similar reaction conditions.^22^ However, it has not thus far been demonstrated that benzamides are competent nucleophiles and can be used directly to form secondary amides. Herein we demonstrate that electrochemical oxidation can successfully generate the required reactive intermediate for direct reaction with benzamides, which are used in near stoichiometric quantities and without activation with base.

To initiate optimization studies, we sought to first identify suitable electrochemical conditions that could be applied toward the oxidation of benzylic substrates in the presence of benzamides. We immediately turned to our previously optimized benzylic acyloxylation conditions,^23^ which involve the use of graphitic carbon electrodes in dichloromethane (DCM) with 2,6-lutidine·HBF_4_ as the supporting electrolyte. Applying these conditions in an undivided cell toward the oxidation of **1a**, but now in the presence of benzamide (2 equiv), gave a 23% yield of amide **3a** (Table 1, entry 1). While it was pleasing to obtain this result, indicating that the transformation could be successfully carried out, we then sought to further improve the yield of **3a**. The main side product observed is the benzyl ketone, which is presumably formed from adventitious water that competes with the amide nucleophile. The possibility of oxygen attack from the benzamide was discounted after not observing any benzonitrile byproduct in the reaction mixture (GCMS). Changing the reaction solvent had a detrimental effect on the yield of **3a**, where lower yields were observed in chloroform, acetonitrile, and THF (entries 2–4). When the cathode material was changed from graphite to nickel or platinum (entries 5 and 6), improved yields of amide **3a** were obtained, with platinum providing the best improvement.^24^ Increasing the current to 20 mA gave no improvement in the yield (entry 7), but increasing the number of equivalents of charge passed first to 5*F* and then to 10*F* gave significant improvements in the yield of **3a** (entries 8 and 9). Two equivalents of benzamide **2a** was found to be optimal because the yield did not change when 4 equiv was used and dropped when 1 equiv was used (entries 9 and 10). Full conversion of **1a** occurred after 10*F* (any further increases in charge passed led to a decline in yield), leading to an optimized yield of 78% (isolated = 73%).

**Table 1:**
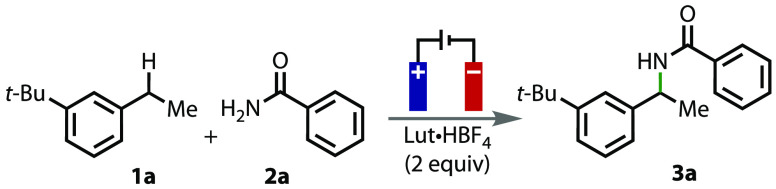
Selected Optimization Data for **3a**^a^

entry^a^	solvent	electrodes (anode:cathode)	current/mA	equiv of charge/*F*	yield of **2a**/%^b^
1	DCM	Gr:Gr	10	2.5	23
2	CHCl_3_	Gr:Gr	10	2.5	7
3	MeCN	Gr:Gr	10	2.5	11
4	THF	Gr:Gr	10	2.5	0
5	DCM	Gr:Ni	10	2.5	27
6	DCM	Gr:Pt	10	2.5	36
7	DCM	Gr:Pt	20	2.5	35
8	DCM	Gr:Pt	10	5.0	56
9^c^	DCM	Gr:Pt	10	5.0	53
10^d^	DCM	Gr:Pt	10	5.0	18
**11**	**DCM**	**Gr:Pt**	**10**	**10.0**	**79**

a Reactions were performed on a 0.2 mmol scale with 2 equiv of **2a**, unless otherwise stated.

b ^1^H NMR yields using 1,3,5-trimethoxybenzene as an internal standard.

c 4 equiv of **2a** was used.

d 1 equiv of **2a** was used.

Having obtained optimized reaction conditions, a range of substrates containing benzylic C(sp^3^)–H bonds were investigated in the reaction (Figure 2). A range of *para*-substituted ethylbenzene substrates were tolerated by the conditions; ethylbenzene and 1-ethyl-4-methylbenzene gave moderate yields of products **3b** and **3c**. Electron-withdrawing groups at the *para* position were less tolerated, as the reaction with 1-ethyl-4-fluorobenzene gave only a 9% yield of the amide product by ^1^H NMR. Furthermore, the reaction with 1-ethyl-4-bromobenzene was unsuccessful, resulting in decomposition of the starting material (see the Supporting Information (SI) for more details). In contrast, a substrate containing an electron-donating group at the *para* position, 1-ethyl-4-methoxybenzene (**1d**), gave an excellent yield of amide **3d**. Reactions with propylbenzene (**1e**) and diphenylmethane (**1f**) gave the desired amide products **3e** and **3f** in good and moderate yields, respectively, with benzophenone being formed as a major side product in the latter case. Bicyclic substrates, indane and tetralin, gave the expected amide products **3g** and **3h** in good yields. Finally, the reaction could be applied toward Celestolide, a synthetic musk, to give amide **3i** in moderate yield. Tertiary benzylic arenes were found to be unsuitable coupling partners in the reaction (see the SI).

**Figure 2:**
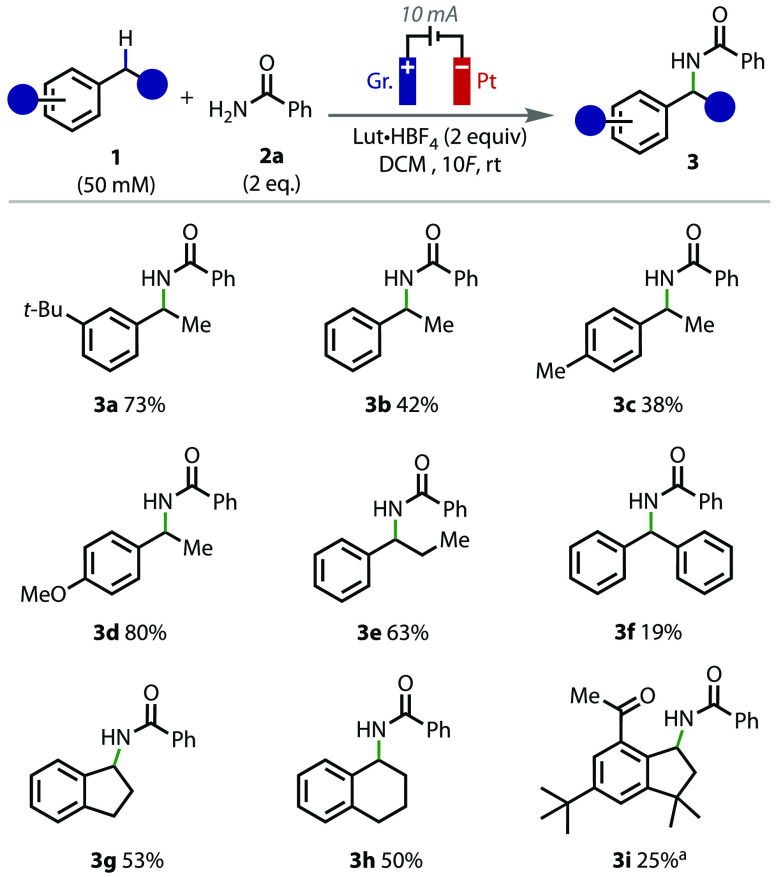
Benzylic substrate scope. All of the listed yields are isolated yields. ^a^4 equiv of **2a** was used for this example.

To investigate the reaction scope further, different substituted benzamides were tested in the reaction. Early on in the process, it became clear that the solubility of the benzamide derivative was important for the success of the reaction. Despite benzamide **2a** being soluble in the reaction medium, benzamides such as 4-bromobenzamide and 4-methoxybenzamide were insoluble. Efforts to use a solvent or solvent blend that could dissolve these substrates were ultimately unsuccessful. Nevertheless, several other benzamides were found to be soluble and therefore were suitable coupling partners, including 3-fluorobenzamide (**2b**), which coupled with several benzylic substrates to give products **3j**–**n** in good to very good yields (Figure 3). Similarly, 3-chlorobenzamide (**2c**) and 3-methylbenzamide (**2d**) reacted with 1-ethyl-4-methoxybenzene to give products **3o** and **3p** in moderate to good yields. Alkyl-substituted benzamides (**2e**–**h**) reacted in good to excellent yields to give benzamides **3p**–**s**, for example, 4-methylbenzamide gave **3q** in 95% isolated yield. Tertiary alkyl amides and secondary amides were found not to be suitable coupling partners, which we propose to be due to steric encumbrance.

**Figure 3:**
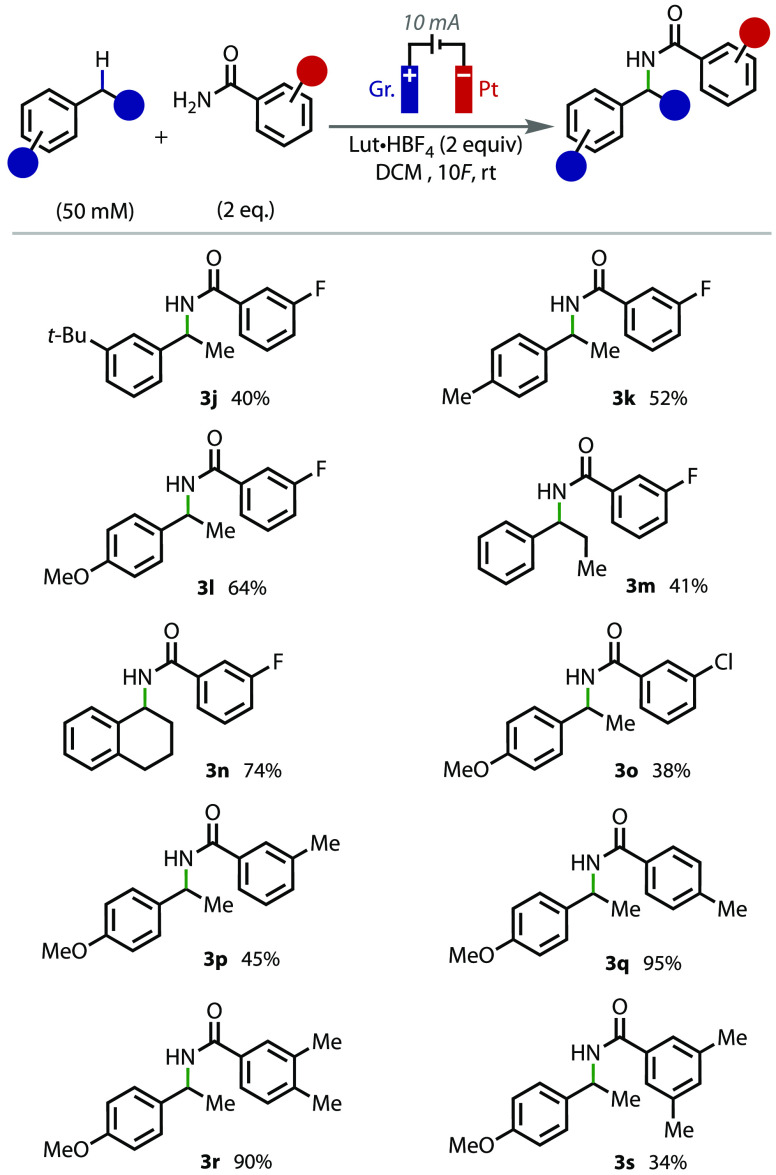
Benzamide substrate scope. All yields listed are isolated yields.

Having investigated the substrate scope, attention was directed toward increasing the scale of the reaction. We elected to use the ElectroVortex reactor,^25^ which consists of a rotating inner electrode with a small but variable annular gap to a static outer electrode. Taylor vortices are generated from the rotation of the inner electrode, which enhances mixing and mass transfer, as is also shown in the corresponding PhotoVortex reactor.^26−28^

As the optimized conditions in batch employed platinum electrodes and moderate concentrations (entry 1, Figure 4B), we conducted a preliminary reoptimization in batch mode of the model reaction of **1g** with **2a** to specifically address these issues. With a higher concentration of **2a** and a stoichiometric parity of substrates (entry 2), the yield decreased, but it improved when the concentration of the benzylic coupling partner was doubled (entry 3) and a higher current was applied (entry 4). We switched the electrodes to materials that are amenable to the ElectroVortex and found that stainless steel (SS) and graphite (Gr) were both suitable (entry 5). Finally, further improvements to the solubility could be achieved by replacing 1 equiv of the 2,6-lutidine·HBF_4_ with TBABF_4_ (entry 6).

**Figure 4:**
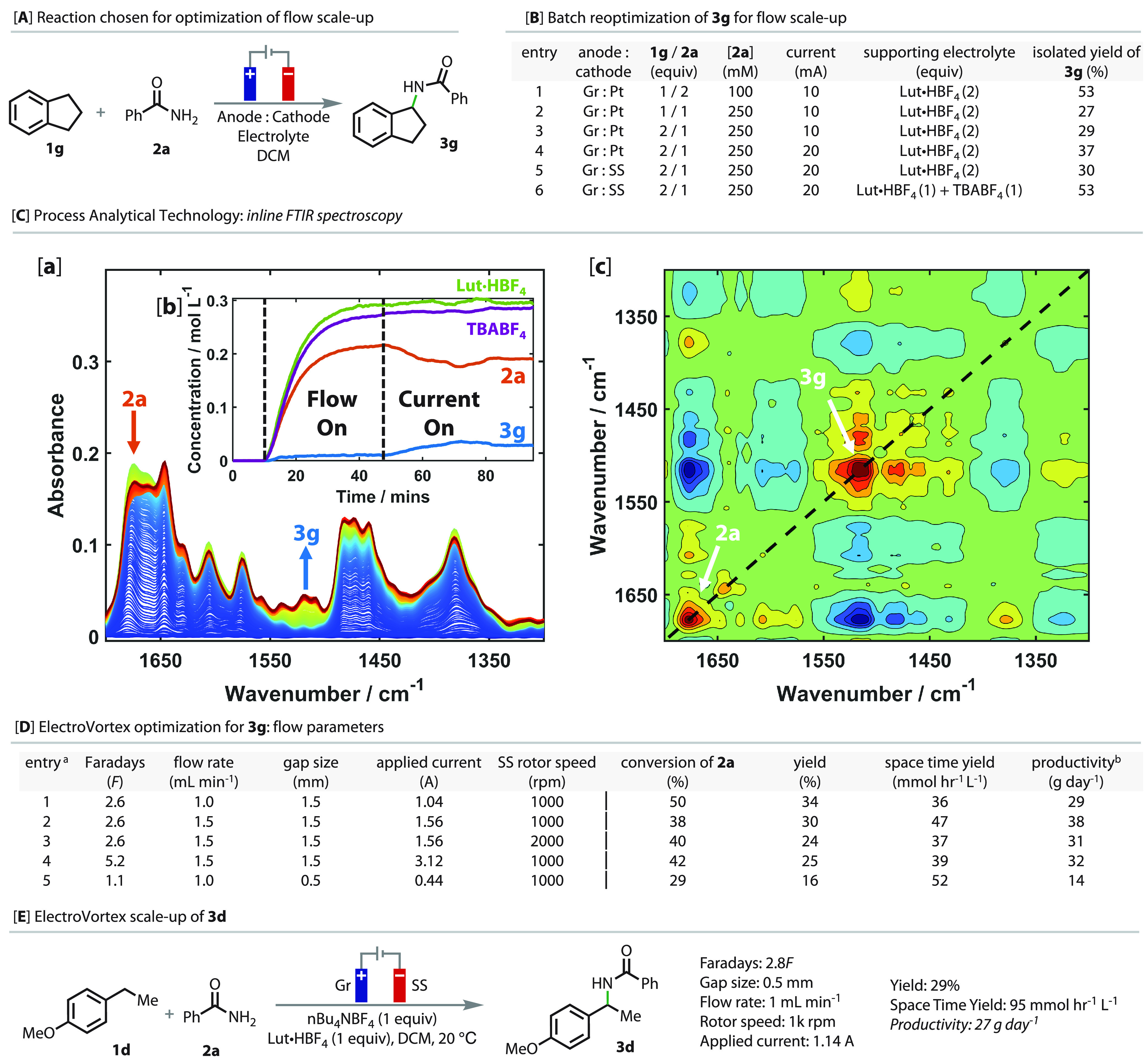
Scale up studies. (A) Reaction chosen for optimization. (B) Reactions performed in the ElectraSyn on a 1 mmol scale with respect to the limiting reagent. The reactions were run at 10 mA and 10*F* at room temperature. (C) (a) FTIR spectra of a flow experiment. Blue spectra show the starting material flowing in, and red spectra show the formation of the product after the current was applied. (b) Concentration profiles of the different compounds from the MCR model. (c) 2D-COS synchronous spectrum showing negative correlation between the characteristic peaks for **2a** and **3g** in the off-diagonal region. (D) Optimization of the electrochemical parameters of the ElectroVortex using 0.25 M benzamide in DCM. ^a^Yields were calculated using quantitative ^1^H NMR with methyl terephthalate as the internal standard. ^b^All productivities are projected over a 24 h continuous run. (E) Scale-up of substrate **1d** to **3d**.

Moving to the ElectroVortex reactor, we used in-line process analysis technology (PAT) to aid rapid optimization and understanding of the flow conditions. In operando FTIR spectroscopy was conducted (Figure 4Ca,b), where the blue spectra show benzamide **2a** flowing through the reactor while red spectra show the reaction at steady state with the product **3g** present. The conversion of **2a** can be seen at ∼1675 cm^–1^ (ν_C=O_), while the peak at ∼1520 cm^–1^ (ν_C–N_ + ν_C–C_ + δ_N–H_) signifies the formation of **3g**. The reaction was challenging to monitor using FTIR due to significant product and benzamide peak overlap. Therefore, a quantitative IR multivariate curve resolution (MCR) model was developed by using calibration solutions. This technique enabled the deconvolution of complex spectra into their components (Figure 4Cc). Two-dimensional correlation spectroscopy (2D-COS) was used to visualize the change in the spectrum as the current was applied. Two positive (red) peaks were observed at 1675 and 1520 cm^–1^, signifying a significant change in these peaks. A negative cross-correlation peak (blue) at 1675 and 1520 cm^–1^ was observed between the two peaks, showing that the consumption of starting material leads to the formation of product. Hence, FT-IR facilitated the rapid screening of conditions and provided information on when steady state was achieved.

Optimization of the electrochemical parameters of the ElectroVortex (Figure 4D) revealed that a 1.5 mm gap size with 4*F* was able to achieve a yield of 34% (entry 1). Increasing the flow rate to 1.5 mL/min improved the projected productivity to 38 g/day with good selectivity over side products. Increasing the rotation speed of the rotor from 1000 to 2000 rpm resulted in a decrease in both yield and selectivity (entry 3), which is likely due to the SS being coated with benzamide, a phenomenon that was observed throughout optimization. Increasing the number of equivalents of charge passed or decreasing the flow rate did not improve the reaction (entries 4 and 5).

4-Ethylanisole was also investigated in the reaction (see the SI for the full details). With a reduced gap size of 0.5 mm (4.6 mL volume), stable conditions could be achieved, leading to a productivity of 27 g day^–1^ (Figure 4E; see the SI for more details). With the reduction in gap size came a greatly increased space-time yield, which is a key performance metric for larger-scale production.^29^

In summary, we have developed a process to trap electrochemically generated benzylic carbocations using benzamide derivatives to produce a range of amide products. Furthermore, this work has been transferred to the ElectroVortex reactor, which has enabled the scale-up of the reaction from milligrams to multigrams in a single-pass flow regime.

## Data Availability

The data underlying this study are available in the published article and its Supporting Information.

## Supporting Information Available

Experimental procedures, characterization data for known and new compounds, and spectra for new compounds (PDF)
