# Supplementary material for: Electrochemical Benzylic C(sp3)–H Direct Amidation
Source: Org Lett. 2024 Jan 16;26(3):653–7. doi: 10.1021/acs.orglett.3c04012 (PMC10825869; doi:10.1021/acs.orglett.3c04012)
Supplement: Supplementary file 1 — ol3c04012_si_001.pdf [file ol3c04012_si_001.pdf]

Supporting Information

Electrochemical Benzylic C(sp^3)-H Direct Amidation

Anthony Choi,[a] Oliver H. Goodrich,[b] Alexander P. Atkins,[a] Matthew D. Edwards,[b] David Tiemessen,[b] Michael W. George,[b] and Alastair J. J. Lennox[a]* (a.lennox@bristol.ac.uk)

[a] *University of Bristol, School of Chemistry, Bristol, BS8 1TS, U.K.*

[b] *University of Nottingham, School of Chemistry, Nottingham, NG7 2RD, U.K*

Contents

General Electrochemistry Setup Information 3

General Procedure 5

Characterisation Data 6

Failed Substrates and Limitations of Benzylic Amidation Reaction 21

Electrochemical Flow Procedure 24

^{1}H, ^{13}C and ^{19}F NMR Spectra 26

References 50

General Experimental Information

Air-sensitive procedures were carried out using Schlenk-line techniques under an atmosphere of N_2. Glassware was dried in a 180 °C oven before use. Chemicals were purchased from Sigma Aldrich, Acros, Fluorochem, Fisher Scientific or Alfa Aesar and used as received unless otherwise stated. Additions of <200 µL were made with Gilson Pipetman pipettes. Anhydrous solvents were collected *via* the Grubbs anhydrous double alumina drying system.

Technical grade solvents were used for column chromatography. Column chromatography was performed using silica gel (230-400 mesh, 60 Å pore size) or *via* a Biotage Selekt with Sfär Silica D Duo capsules. TLC analysis was performed using SiO_2 coated aluminium plates visualized by UV fluorescence or potassium permanganate stains. Unless stated otherwise, petrol refers to petroleum ether 40-60 °C.

NMR samples were submitted in $CDCl_3$ (Sigma Aldrich) and spectra were recorded on Bruker Nano 400, Jeol ECS 300, Jeol ECS 400, Jeol ECZ 400, Varian 500 or Bruker Advance III HD 500 Cryo spectrometers. Chemical shifts are reported in parts per million (ppm) and referenced to residual solvent. Coupling constants (J) are quoted in Hz to the nearest 0.5 Hz. Multiplicities are reported as s (singlet), d (doublet), t (triplet), q (quartet) and m (multiplet).

IR analysis was performed on a PerkinElmer Spectrum 100 FTIR with an ATR accessory and frequencies reported in wavenumbers (cm^{-1}). Online IR analysis was obtained using a Mettler Toledo ReactIR 702L with a 6.3 mm fibreoptic probe coupled to a flow cell. High Resolution Mass Spectrometry was recorded on QExactive (GC-Orbitrap), Orbitrap Elite (LC-Orbitrap) or Synapt G2S (IMS-Q-TOF) instruments using electrospray ionisation (ESI). Samples were submitted in CH2Cl2High Resolution Mass Spectrometry was recorded on QExactive (GC-Orbitrap), Orbitrap Elite (LC-Orbitrap) or Synapt G2S (IMS-Q-TOF) instruments using electrospray ionisation (ESI). Samples were submitted in CH_2Cl_2

Electrolysis was performed using an IKA ElectraSyn 2.0 with ElectraSyn electrodes, lids and reaction vials. Graphite electrodes were sonicated in a 50/50 mixture of EtOH/Acetone and dried using a heat gun before use. If material had deposited onto the electrode surface a fine sandpaper was used to remove it before washing. CV analysis was performed using a Palmsens Multisens 4 with a glassy carbon working electrode, platinum wire counter electrode and a 0.1 M $Ag/AgNO_3$ reference electrode. All electrodes were polished and the reaction mixture was stirred and degassed by a stream of N_2 for approximately 90 seconds before each CV.

General Electrochemistry Setup Information

Construction of Pt Counter Electrode Clip: A 1000 μL Blue Gilson pipette tip was cut 10 mm and 20 mm from the end of the tip as shown in **Figure S1 (A)**. The 10 mm middle section was used as the Pt Electrode Clip.

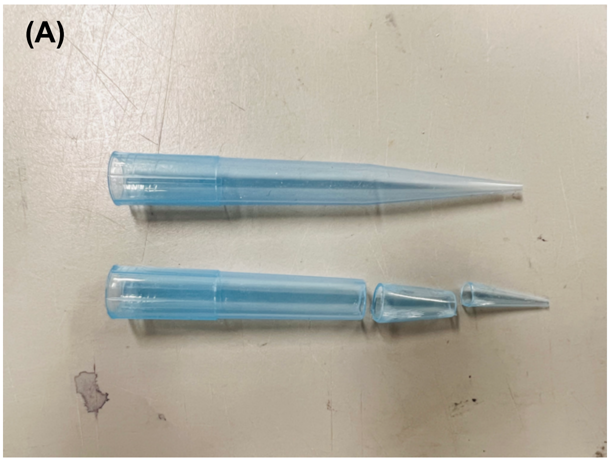

Figure S1

Construction of Pt Counter Electrode: 300 mm of Pt wire (0.4 mm diameter from Advent Research Materials, Product Code: PT5441) was wrapped around a standard NMR tube 15 times to produce a coil as shown in **Figure S2 (B)**. The coil was flattened to give the electrode shown in **Figure S2 (C)**. The other end of the Pt wire was placed through the Pt Electrode Clip as shown in **Figure S2 (D)**. The remainder of the Pt wire was wrapped around a standard NMR tube to complete the electrode as shown in **Figure S2 (E)**.

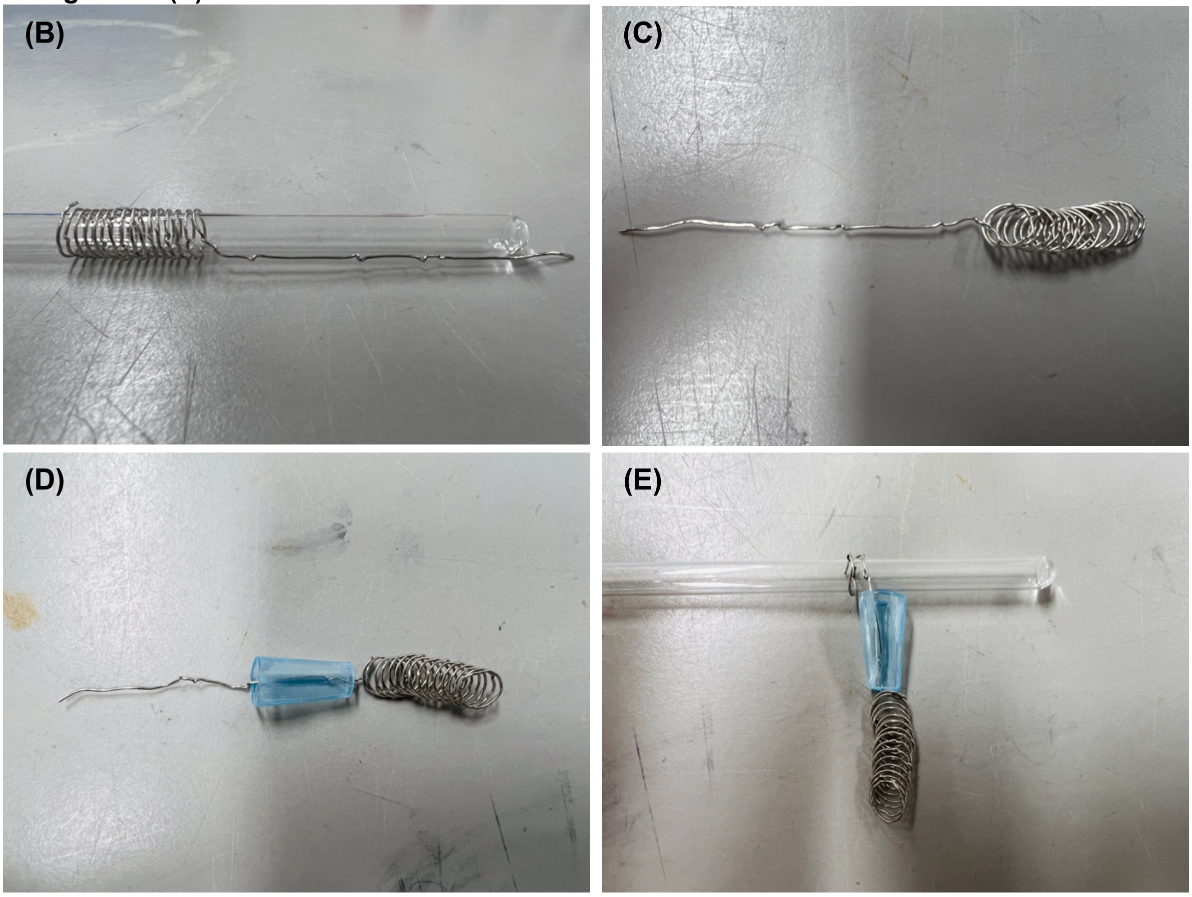

Figure S2

Assembly of Apparatus: The materials used to setup reactions on the ElectraSyn 2.0 are shown in **Figure S3 (F)**. Left to Right: 5 mL ElectraSyn vial, graphite ElectraSyn electrode, Pt counter electrode, ElectraSyn cap, Parafilm strip (12 cm x 2.5 cm), balloon attached to syringe barrel.

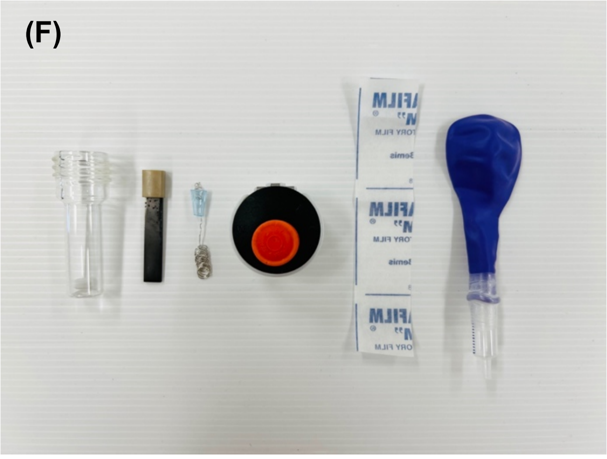

Figure S3

The ElectraSyn cap was modified by replacement of the commercial seal with a B14 septum. The Pt counter electrode was attached to the cap followed by the graphite ElectraSyn electrode as shown in **Figure (G)**. Once reagents and solvent were placed in the ElectraSyn vial the cap was screwed on and the setup was sealed with Parafilm as shown in **Figure (H)**. The reaction mixture was degassed through the B14 septum with N_2 and the electrochemical reaction was initiated as shown in **Figure (I)**.

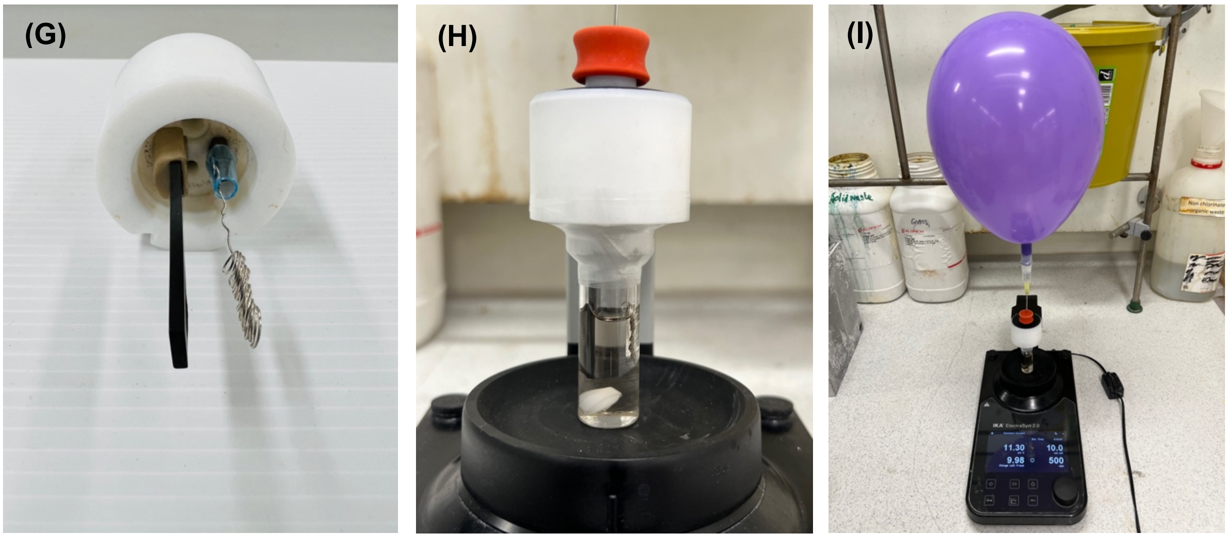

Figure S4

General Procedure

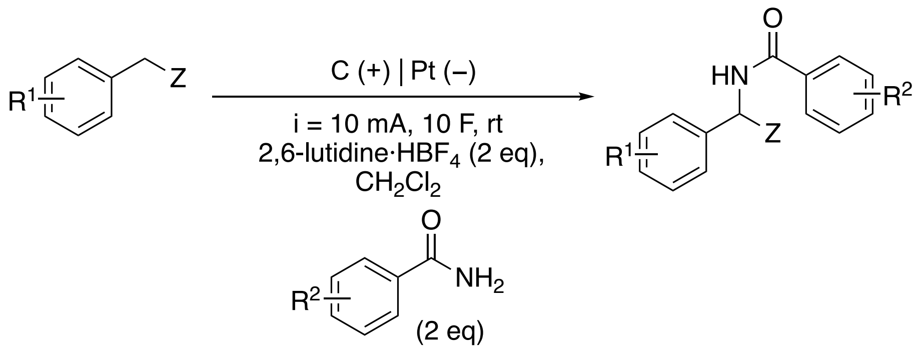

To a 5 mL oven-dried ElectraSyn vial equipped with a magnetic stirrer bar was added substrate (0.2 mmol, 1 equiv.), primary amide (0.4 mmol, 2 equiv.), 2,6-lutidine•HBF_4 (78 mg, 0.4 mmol, 2 equiv.) and DCM (4 mL). The connection between the ElectraSyn vial and cap was sealed with parafilm and the reaction mixture was degassed with N_2. An N_2 balloon was placed through the septum and the reaction was subjected to electrolysis on the ElectraSyn 2.0 at 10 mA constant current for 10 *F*. The reaction mixture was partitioned between DCM (50 mL) and water (25 mL) and the electrode were rinsed with DCM (~20 mL). The organic layer was separated, dried over $MgSO_4$, filtered and concentrated under reduced pressure to give the crude product. Purification by column chromatography on silica gel gave the secondary amide product.

Characterisation Data

*N-(1-(3-(tert-Butyl)phenyl)ethyl)benzamide (**3a**)*

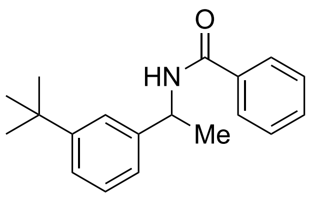

Title compound **3a** was prepared using the general procedure using 1-*tert*-butyl-3-ethylbenzene (38 μL, 0.2 mmol) and benzamide (49 mg, 0.4 mmol). After column chromatography on silica gel eluting with 15% EtOAc in *n*-pentane gave amide **3a** (41 mg, 73%) as an amorphous brown solid.

R_f = 0.43 (3:7 EtOAc–petrol)

^{1}H NMR (400 MHz, $CDCl_3$): δ = 7.79–7.74 (m, 2H), 7.52–7.46 (m, 1H), 7.45–7.38 (m, 3H), 7.35–7.28 (m, 2H), 7.22 (dt, *J* = 7.0, 2.0 Hz, 1H), 6.34 (br d, *J* = 7.0 Hz, 1H), 5.35 (quin, *J* = 7.0 Hz, 1H), 1.62 (d, *J* = 7.0 Hz, 3H), 1.33 (s, 9H).

^{13}C NMR (101 MHz, $CDCl_3$): δ = 166.7, 151.9, 142.8, 134.9, 131.6, 128.7, 128.6, 127.0, 124.7, 123.7, 123.2, 49.7, 34.9, 31.5, 21.9.

HRMS (ESI) m/z calc: [M+Na]+ ($C_{19}H_{23}NONa$) 304.1677; measured: 304.1671 = 2.0 ppm difference

IR (FTIR, cm^{-1}): ν_{max} = 3317 (N–H), 2960, 1634 (C=O), 1539, 1490

N-(1-Phenylethyl)benzamide (**3b**)

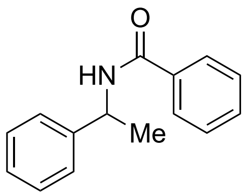

Title compound **3b** was prepared using the general procedure using ethylbenzene (24 μL, 0.2 mmol) and benzamide (49 mg, 0.4 mmol). After column chromatography on silica gel eluting with 15% EtOAc in *n*-pentane gave amide **3b** (19 mg, 42%) as an orange oil.

R_f = 0.14 (1:4 EtOAc–petrol)

^{1}H NMR (400 MHz, $CDCl_3$): δ = 7.79–7.75 (m, 2H), 7.48 (dt, *J* = 3.0, 2.0 Hz, 1H), 7.45–7.35 (m, 6H), 7.29 (dt, *J* = 5.0, 2.0 Hz, 1H), 6.33 (br s, 1H), 5.41–5.26 (m, 1H), 1.61 (d, *J* = 7.0 Hz, 3H).

^{13}C NMR (101 MHz, $CDCl_3$): δ = 166.7, 143.3, 134.8, 131.6, 128.9, 128.7, 127.6, 127.1, 126.4, 49.4, 21.9.

Data consistent with that reported in the literature [1]

N-(1-(*p*-Tolyl)ethyl)benzamide (**3c**)

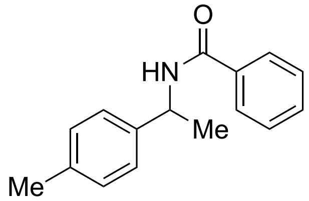

Title compound **3c** was prepared using the general procedure using 1-ethyl-4-methylbenzene (28 μL, 0.2 mmol) and benzamide (49 mg, 0.4 mmol). After column chromatography on silica gel eluting with 10% EtOAc in *n*-pentane gave amide **3c** (18 mg, 38%) as a brown oil.

R_f = 0.19 (1:4 EtOAc–petrol)

^{1}H NMR (400 MHz, $CDCl_3$): δ = 7.78–7.74 (m, 2H), 7.50–7.46 (m, 1H), 7.44–7.39 (m, 2H), 7.29 (d, *J* = 8.0 Hz, 2H), 7.17 (d, *J* = 8.0 Hz, 2H), 6.31 (br d, *J* = 6.5 Hz, 1H), 5.31 (quin, *J* = 7.0 Hz, 1H), 2.34 (s, 3H), 1.60 (d, *J* = 7.0 Hz, 3H).

^{13}C NMR (101 MHz, $CDCl_3$): δ = 166.6, 140.3, 137.3, 134.8, 131.6, 129.6, 128.7, 127.0, 126.4, 49.1, 21.8, 21.2.

Data consistent with that reported in the literature [1]

N-(1-(4-Methoxyphenyl)ethyl)benzamide (**3d**)

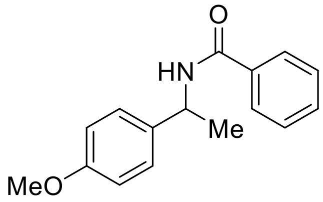

Title compound **3d** was prepared using the general procedure using 1-ethyl-4-methoxybenzene (28 μL, 0.2 mmol) and benzamide (49 mg, 0.4 mmol). After column chromatography on silica gel eluting with 20% EtOAc in *n*-pentane gave amide **3d** (41 mg, 80%) as a pale orange oil.

R_f = 0.11 (1:4 EtOAc–petrol)

^{1}H NMR (400 MHz, $CDCl_3$): δ = 7.78–7.74 (m, 2H), 7.51–7.45 (m, 1H), 7.44–7.38 (m, 2H), 7.35–7.30 (m, 2H), 6.91–6.87 (m, 2H), 6.26 (br d, *J* = 7.5 Hz, 1H), 5.30 (quin, *J* = 7.0 Hz, 1H), 3.80 (s, 3H), 1.60 (d, *J* = 7.0 Hz, 3H).

^{13}C NMR (101 MHz, $CDCl_3$): δ = 166.6, 159.1, 135.4, 134.8, 131.6, 128.7, 127.6, 127.0, 114.3, 55.5, 48.8, 21.7.

Data consistent with that reported in the literature [1]

N-(1-Phenylpropyl)benzamide (**3e**)

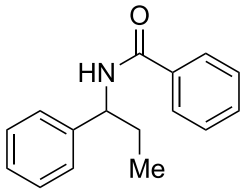

Title compound **3e** was prepared using the general procedure using propylbenzene (28 µL, 0.2 mmol) and benzamide (49 mg, 0.4 mmol). After column chromatography on silica gel eluting with 15% EtOAc in *n*-pentane gave amide **3e** (30 mg, 63%) as a brown oil.

R_f = 0.31 (1:4 EtOAc–petrol)

^{1}H NMR (400 MHz, $CDCl_3$): δ = 7.80–7.74 (m, 2H), 7.52–7.46 (m, 1H), 7.46–7.38 (m, 3H), 7.37–7.33 (m, 3H), 7.32–7.27 (m, 1H), 6.34 (br s, 1H), 5.16–5.02 (m, 1H), 2.07–1.84 (m, 2H), 0.96 (t, *J* = 7.5 Hz, 3H).

^{13}C NMR (101 MHz, $CDCl_3$): δ = 166.9, 142.2, 134.9, 131.6, 128.9, 128.72, 128.69, 127.6, 127.0, 126.8, 55.5, 29.3, 10.9.

Data consistent with that reported in the literature [2]

N-Benzhydrylbenzamide (**3f**)

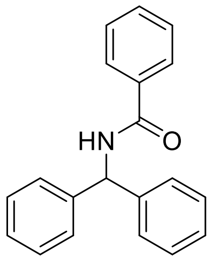

Title compound **3f** was prepared using the general procedure using diphenylmethane (30 µL, 0.2 mmol) and benzamide (49 mg, 0.4 mmol). After column chromatography on silica gel eluting with 10% EtOAc in *n*-pentane gave amide **3f** (11 mg, 19%) as an off-white amorphous solid.

R_f = 0.19 (1:4 EtOAc–petrol)

^{1}H NMR (400 MHz, $CDCl_3$): δ = 7.86–7.79 (m, 2H), 7.55–7.48 (m, 1H), 7.47–7.41 (m, 2H), 7.39–7.27 (m, 10H), 6.68 (br d, *J* = 7.0 Hz, 1H), 6.46 (d, *J* = 8.0 Hz, 1H).

^{13}C NMR (101 MHz, $CDCl_3$): δ = 166.6, 141.6, 134.4, 131.9, 128.9, 128.8, 127.73, 127.65, 127.2, 57.6.

Data consistent with that reported in the literature [1]

N-(2,3-Dihydro-1H-inden-1-yl)benzamide (**3g**)

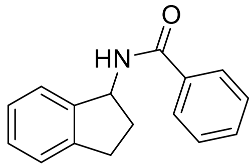

Title compound **3g** was prepared using the general procedure using indane (25 µL, 0.2 mmol) and benzamide (49 mg, 0.4 mmol). After column chromatography on silica gel eluting with 15% EtOAc in *n*-pentane gave amide **3g** (25 mg, 53%) as a pale orange oil.

R_f = 0.21 (1:4 EtOAc–petrol)

^{1}H NMR (400 MHz, $CDCl_3$): δ = 7.74–7.68 (m, 2H), 7.44–7.39 (m, 1H), 7.37–7.31 (m, 2H), 7.27 (d, *J* = 7.5 Hz, 1H), 7.21–7.17 (m, 2H), 7.17–7.11 (m, 1H), 6.35 (br d, *J* = 7.5 Hz, 1H), 5.60 (q, *J* = 7.5 Hz, 1H), 2.99–2.90 (m, 1H), 2.89–2.79 (m, 1H), 2.66–2.56 (m, 1H), 1.90–1.79 (m, 1H).

^{13}C NMR (101 MHz, $CDCl_3$): δ = 167.3, 143.7, 143.3, 134.6, 131.6, 128.7, 128.2, 127.1, 127.0, 125.0, 124.2, 55.3, 34.3, 30.4.

Data consistent with that reported in the literature [3]

N-(1,2,3,4-Tetrahydronaphthalen-1-yl)benzamide (**3h**)

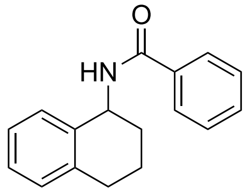

Title compound **3h** was prepared using the general procedure using 1,2,3,4-tetrahydronaphthalene (27 µL, 0.2 mmol) and benzamide (49 mg, 0.4 mmol). After column chromatography on silica gel eluting with 15% EtOAc in *n*-pentane gave amide **3h** (25 mg, 50%) as a yellow oil.

R_f = 0.24 (1:4 EtOAc–petrol)

^{1}H NMR (400 MHz, $CDCl_3$) : δ = 7.80–7.75 (m, 2H), 7.53–7.47 (m, 1H), 7.46–7.40 (m, 2H), 7.37–7.32 (m, 1H), 7.23–7.16 (m, 2H), 7.16–7.11 (m, 1H), 6.32 (br d, *J* = 7.5 Hz, 1H), 5.44–5.36 (m, 1H), 2.90–2.77 (m, 2H), 2.20–2.10 (m, 1H), 1.99–1.86 (m, 3H).

^{13}C NMR (101 MHz, $CDCl_3$): δ = 166.8, 137.9, 136.8, 134.8, 131.6, 129.4, 128.9, 128.7, 127.5, 127.1, 126.5, 48.1, 30.3, 29.4, 20.2.

Data consistent with that reported in the literature [4]

*N-(7-Acetyl-5-(tert-butyl)-3,3-dimethyl-2,3-dihydro-1H-inden-1-yl)benzamide (**3i**)*

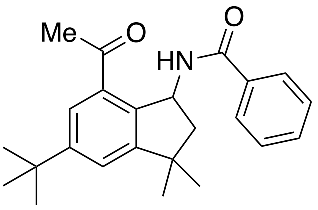

Title compound **3i** was prepared using the general procedure using celestonide (73 mg, 0.2 mmol) and benzamide (98 mg, 0.8 mmol). After column chromatography on silica gel eluting with 20% EtOAc in *n*-pentane gave amide **3i** (18 mg, 25%) as an amorphous white solid.

R_f = 0.26 (1:4 EtOAc–pentane)

^{1}H NMR (400 MHz, $CDCl_3$): δ = 7.75–7.71 (m, 2H), 7.61 (d, *J* = 2.0 Hz, 1H), 7.49–7.44 (m, 1H), 7.42–7.36 (m, 3H), 6.44 (br d, *J* = 6.0 Hz, 1H), 5.80–5.72 (m, 1H), 2.56 (s, 3H), 2.51 (dd, *J* = 13.5, 7.5 Hz, 1H), 2.17 (dd, *J* = 13.5, 4.5 Hz, 1H), 1.38 (overlapping s, 12H), 1.33 (s, 3H).

^{13}C NMR (101 MHz, $CDCl_3$): δ = 201.8, 167.2, 155.2, 152.9, 137.1, 136.3, 134.9, 131.5, 128.7, 127.1, 124.9, 123.3, 53.9, 49.3, 42.8, 35.1, 31.6, 31.0, 29.9, 29.1.

HRMS (ESI) m/z calc: [M+H]+ ($C_{24}H_{30}NO_2$) 364.2271; measured: 364.2273 = 0.6 ppm difference

IR (FTIR, cm^{-1}): ν_{max} = 3301, 2957, 2866, 1683, 1636, 1527, 1480, 1363, 1234, 730

*N-(1-(3-(tert-Butyl)phenyl)ethyl)-3-fluorobenzamide (**3j**)*

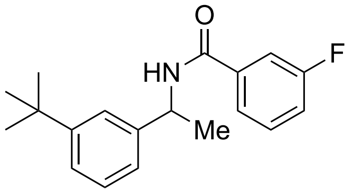

Title compound **3j** was prepared using the general procedure using 1-*tert*-butyl-3-ethylbenzene (38 μL, 0.2 mmol) and 3-fluorobenzamide (56 mg, 0.4 mmol). After column chromatography on silica gel eluting with 15% EtOAc in *n*-pentane gave amide **3j** (24 mg, 40%) as an amorphous orange solid.

R_f = 0.26 (1:4 EtOAc–petrol)

^{1}H NMR (400 MHz, $CDCl_3$): δ = 7.58–7.45 (m, 2H), 7.43–7.28 (m, 4H), 7.24–7.14 (m, 2H), 6.32 (br d, *J* = 7.0 Hz, 1H), 5.32 (quin, *J* = 7.0 Hz, 1H), 1.62 (d, *J* = 7.0 Hz, 3H), 1.33 (s, 9H).

^{13}C NMR (101 MHz, $CDCl_3$): δ = 165.4, 162.9 (d, *J* = 248 Hz), 151.9, 142.5, 137.2 (d, *J* = 7.0 Hz), 130.4 (d, *J* = 8.0 Hz), 128.7, 124.9, 123.7, 123.2, 122.5 (d, *J* = 3.0 Hz), 118.6 (d, *J* = 21 Hz), 114.5 (d, *J* = 22.8 Hz), 49.9, 34.9, 31.5, 21.8.

^{19}F NMR (377 MHz, $CDCl_3$): δ = −111.81.

HRMS (ESI) m/z calc: [M+H]+ ($C_{19}H_{23}NOF$) 300.1758; measured: 300.1748 = 3.3 ppm difference

IR (FTIR, cm^{-1}): ν_{max} = 3311 (N–H), 2963, 1730, 1636 (C=O), 1586, 1537, 1483, 1270

*3-Fluoro-N-(1-(p-tolyl)ethyl)benzamide (**3k**)*

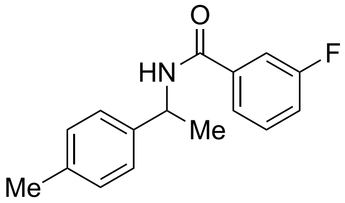

Title compound **3k** was prepared using the general procedure using 1-ethyl-4-methylbenzene (28 μL, 0.2 mmol) and 3-fluorobenzamide (56 mg, 0.4 mmol). After column chromatography on silica gel eluting with 15% EtOAc in *n*-pentane gave amide **3k** (27 mg, 52%) as a brown oil.

R_f = 0.26 (1:4 EtOAc–petrol)

^{1}H NMR (400 MHz, $CDCl_3$): δ = 7.52–7.46 (m, 2H), 7.40–7.34 (m, 1H), 7.29–7.26 (m, 2H), 7.20–7.14 (m, 3H), 6.37 (br d, *J* = 7.0 Hz, 1H), 5.28 (quin, *J* = 7.0 Hz, 1H), 2.34 (s, 3H), 1.59 (d, *J* = 7.0 Hz, 3H).

^{13}C NMR (101 MHz, $CDCl_3$): δ = 165.4, 162.9 (d, *J* = 248 Hz), 140.0, 137.5, 137.1 (d, *J* = 7.0 Hz), 130.3 (d, *J* = 8.0 Hz), 129.6, 126.3, 122.5 (d, *J* = 3.0 Hz), 118.6 (d, *J* = 21 Hz), 114.5 (d, *J* = 23 Hz), 49.3, 21.7, 21.2.

^{19}F NMR (377 MHz, $CDCl_3$): δ = −111.84.

HRMS (ESI) m/z calc: [M+Na]+ ($C_{16}H_{16}NOFNa$) 280.1114; measured: 280.1116 = 0.7 ppm difference

IR (FTIR, cm^{-1}): ν_{max} = 3286 (N–H), 3068, 2974, 2927, 2873, 1898, 1792, 1669 (C=O), 1582, 1535, 1484, 1278, 1227, 1110, 817, 755, 677,

*3-Fluoro-N-(1-(4-methoxyphenyl)ethyl)benzamide (**3l**)*

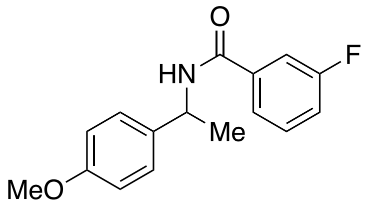

Title compound **3l** was prepared using the general procedure using 1-ethyl-4-methoxybenzene (28 μL, 0.2 mmol) and 3-fluorobenzamide (56 mg, 0.4 mmol). After column chromatography on silica gel eluting with 20% EtOAc in *n*-pentane gave amide **3l** (35 mg, 64%) as a brown oil.

R_f = 0.16 (1:4 EtOAc–petrol)

^{1}H NMR (400 MHz, $CDCl_3$): δ = 7.52–7.46 (m, 2H), 7.41–7.35 (m, 1H), 7.33–7.29 (m, 2H), 7.21–7.14 (m, 1H), 6.92–6.86 (m, 2H), 6.32 (br d, *J* = 7.0 Hz, 1H), 5.27 (quin, *J* = 7.0 Hz, 1H), 3.80 (s, 3H), 1.59 (d, *J* = 7.0 Hz, 3H).

^{13}C NMR (101 MHz, $CDCl_3$): δ = 165.3, 162.9 (d, *J* = 248 Hz), 159.1, 137.1 (d, *J* = 7.0 Hz), 135.1, 130.3 (d, *J* = 8.0 Hz), 127.6, 122.5 (d, *J* = 3.0 Hz), 118.5 (d, *J* = 21.5 Hz), 114.5 (d, *J* = 23 Hz), 114.3, 55.4, 49.0, 21.6.

^{19}F NMR (377 MHz, $CDCl_3$): δ = −111.85.

HRMS (ESI) m/z calc: [M+H]+ ($C_{16}H_{17}NO_2F$) 274.1238; measured: 274.1228 = 3.7 ppm difference

IR (FTIR, cm^{-1}): ν_{max} = 3290 (N–H), 3070, 2875, 2827, 2067, 1874, 1734, 1627 (C=O), 1537, 1519, 1479, 1267, 1240, 1182, 1124, 1038, 831, 805, 751.

*3-Fluoro-N-(1-phenylpropyl)benzamide (**3m**)*

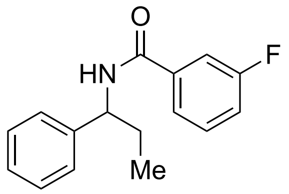

Title compound **3m** was prepared using the general procedure using propylbenzene (28 µL, 0.2 mmol) and 3-fluorobenzamide (56 mg, 0.4 mmol). After column chromatography on silica gel eluting with 15% EtOAc in *n*-pentane gave amide **3m** (21 mg, 41%) as an amorphous orange solid.

R_f = 0.33 (1:4 EtOAc–petrol)

^{1}H NMR (400 MHz, $CDCl_3$): δ = 7.54–7.45 (m, 2H), 7.43–7.38 (m, 1H), 7.37–7.34 (m, 3H), 7.32–7.27 (m, 2H), 7.22–7.15 (m, 1H), 6.26 (br d, *J* = 7.0 Hz, 1H), 5.13–5.03 (m, 1H), 2.05–1.86 (m, 2H), 0.96 (t, *J* = 7.5 Hz, 3H).

^{13}C NMR (101 MHz, $CDCl_3$, three quaternary carbon signals not observed): δ = 168.2, 141.9, 130.4 (d, *J* = 8.0 Hz), 128.9, 127.7, 126.8, 122.5 (d, *J* = 3.0 Hz), 118.6 (d, *J* = 21.5 Hz), 114.5 (d, *J* = 22.5 Hz), 55.7, 29.3, 11.0.

^{19}F NMR (377 MHz, $CDCl_3$): δ = −111.79

HRMS (ESI) m/z calc: [M+H]+ ($C_{16}H_{17}NOF$) 258.1289; measured: 258.1280 = 3.5 ppm difference

IR (FTIR, cm^{-1}): ν_{max} = 3283 (N–H), 3062, 2965, 2931, 1634 (C=O), 1586, 1536, 1483, 1303, 1222

*3-Fluoro-N-(1,2,3,4-tetrahydronaphthalen-1-yl)benzamide (**3n**)*

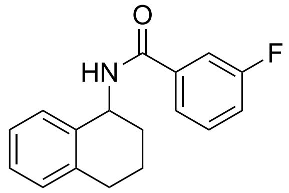

Title compound **3n** was prepared using the general procedure using 1,2,3,4-tetrahydronaphthalene (27 μL, 0.2 mmol) and 3-fluorobenzamide (56 mg, 0.4 mmol). After column chromatography on silica gel eluting with 15% EtOAc in *n*-pentane gave amide **3n** (40 mg, 74%) as an amorphous brown solid.

R_f = 0.30 (1:4 EtOAc–petrol)

^{1}H NMR (400 MHz, $CDCl_3$): δ = 7.56–7.47 (m, 2H), 7.43–7.36 (m, 1H), 7.35–7.29 (m, 1H), 7.24–7.10 (m, 4H), 6.31 (br d, *J* = 7.5 Hz, 1H), 5.45–5.32 (m, 1H), 2.92–2.74 (m, 2H), 2.21–2.08 (m, 1H), 2.02–1.82 (m, 3H).

^{13}C NMR (101 MHz, $CDCl_3$): δ = 165.5, 163.0 (d, *J* = 248 Hz), 137.9, 137.1 (d, *J* = 7.0 Hz), 136.5, 130.4 (d, *J* = 8.0 Hz), 129.5, 128.9, 127.7, 126.6, 122.5 (d, *J* = 3.0 Hz), 118.6 (d, *J* = 21.5 Hz), 114.6 (d, *J* = 23 Hz), 48.3, 30.3, 29.4, 20.1.

^{19}F NMR (377 MHz, $CDCl_3$): δ = −111.76.

HRMS (ESI) m/z calc: [M+H]+ ($C_{17}H_{17}NOF$) 270.1289; measured: 270.1278 = 4.4 ppm difference

IR (FTIR, cm^{-1}): ν_{max} = 3287 (N–H), 3064, 2935, 1634 (C=O), 1585, 1536, 1482, 1270

*3-Chloro-N-(1-(4-methoxyphenyl)ethyl)benzamide (**3o**)*

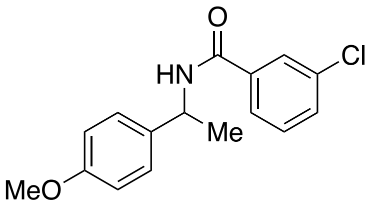

Title compound **3o** was prepared using the general procedure using 1-ethyl-4-methoxybenzene (28 μL, 0.2 mmol) and 3-chlorobenzamide (62 mg, 0.4 mmol). After column chromatography on silica gel eluting with 20% EtOAc in *n*-pentane gave amide **3o** (22 mg, 38%) as a brown oil.

R_f = 0.13 (1:4 EtOAc–petrol)

^{1}H NMR (400 MHz, $CDCl_3$): δ = 7.73 (t, *J* = 2.0 Hz, 1H), 7.64–7.59 (m, 1H), 7.45 (ddd, *J* = 8.0, 2.0, 1.0 Hz, 1H), 7.37–7.28 (m, 3H), 6.91–6.86 (m, 2H), 6.26 (br d, *J* = 7.0 Hz, 1H), 5.27 (quin, *J* = 7.0 Hz, 1H), 3.80 (s, 3H), 1.59 (d, *J* = 7.0 Hz, 3H).

^{13}C NMR (101 MHz, $CDCl_3$): δ = 165.3, 159.2, 136.6, 135.0, 134.9, 131.6, 130.0, 127.6, 127.4, 125.2, 114.3, 55.5, 49.0, 21.6.

HRMS (ESI) m/z calc: [M+H]+ ($C_{16}H_{17}NO_2Cl$) 290.0942; measured: 290.0933 = 3.1 ppm difference

IR (FTIR, cm^{-1}): ν_{max} = 3286 (N–H), 3064, 2970, 2932, 2834, 2053, 1883, 1735, 1633 (C=O), 1533, 1511, 1469, 1324, 1245, 1105, 1036, 830, 809, 747.

*N-(1-(4-Methoxyphenyl)ethyl)-3-methylbenzamide (**3p**)*

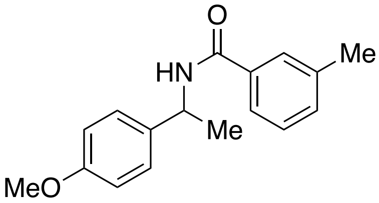

Title compound **3p** was prepared using the general procedure using 1-ethyl-4-methoxybenzene (28 μL, 0.2 mmol) and 3-methylbenzamide (54 mg, 0.4 mmol). After column chromatography on silica gel eluting with 20% EtOAc in *n*-pentane gave amide **3p** (24 mg, 45%) as a brown oil.

R_f = 0.16 (1:4 EtOAc–petrol)

^{1}H NMR (400 MHz, $CDCl_3$): δ = 7.58 (s, 1H), 7.56–7.50 (m, 1H), 7.36–7.27 (m, 4H), 6.92–6.85 (m, 2H), 6.31 (br d, *J* = 7.5 Hz, 1H), 5.29 (quin, *J* = 7.0 Hz, 1H), 3.80 (s, 3H), 2.38 (s, 3H), 1.59 (d, *J* = 7.0 Hz, 3H).

^{13}C NMR (101 MHz, $CDCl_3$): δ = 166.8, 159.0, 138.5, 135.4, 134.8, 132.3, 128.5, 127.8, 127.6, 124.0, 114.2, 55.4, 48.7, 21.7, 21.4.

HRMS (ESI) m/z calc: [M+H]+ ($C_{17}H_{20}NO_2$) 270.1489; measured: 270.1480 = 3.3 ppm difference

IR (FTIR, cm^{-1}): ν_{max} = 3303 (N–H), 3052, 2975, 2925, 2836, 1955, 1879, 1730, 1636 (C=O), 1609, 1527, 1456, 1276, 1249, 1178, 1038, 836, 746, 701.

*N-(1-(4-Methoxyphenyl)ethyl)-4-methylbenzamide (**3q**)*

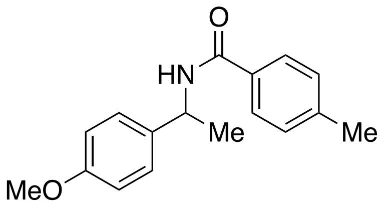

Title compound **3q** was prepared using the general procedure using 1-ethyl-4-methoxybenzene (28 μL, 0.2 mmol) and *p*-toluamide (54 mg, 0.4 mmol). After column chromatography on silica gel eluting with 20% EtOAc in *n*-pentane gave amide **3q** (51 mg, 95%) as a brown oil.

R_f = 0.11 (1:4 EtOAc–petrol)

^{1}H NMR (400 MHz, $CDCl_3$): δ = 7.66 (d, *J* = 8.0 Hz, 2H), 7.33–7.29 (m, 2H), 7.20 (d, *J* = 8.0 Hz, 2H), 6.91–6.85 (m, 2H), 6.37 (br d, *J* = 7.5 Hz, 1H), 5.28 (quin, *J* = 7.0 Hz 1H), 3.79 (s, 3H), 2.38 (s, 3H), 1.57 (d, *J* = 7.0 Hz, 3H).

^{13}C NMR (101 MHz, $CDCl_3$): δ = 166.6, 159.0, 141.9, 135.5, 131.9, 129.3, 127.6, 127.0, 114.1, 55.4, 48.6, 21.7, 21.5.

HRMS (ESI) m/z calc: [M+H]+ ($C_{17}H_{20}NO_2$) 270.1489; measured: 270.1482 = 2.6 ppm difference

IR (FTIR, cm^{-1}): ν_{max} = 3348 (N–H), 2971, 2930, 2836, 2054, 1973, 1901, 1636 (C=O), 1519, 1505, 1447, 1249, 1182, 1025, 827, 764.

*N-(1-(4-Methoxyphenyl)ethyl)-3,4-dimethylbenzamide (**3r**)*

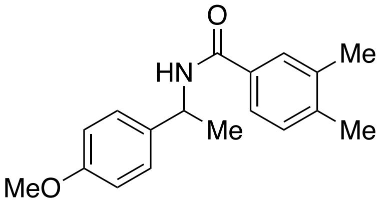

Title compound **3r** was prepared using the general procedure using 1-ethyl-4-methoxybenzene (28 μL, 0.2 mmol) and 3,4-dimethylbenzamide (60 mg, 0.4 mmol). After column chromatography on silica gel eluting with 20% EtOAc in *n*-pentane gave amide **3r** (51 mg, 90%) as a brown oil.

R_f = 0.16 (1:4 EtOAc–petrol)

^{1}H NMR (400 MHz, $CDCl_3$): δ = 7.55 (s, 1H), 7.48 (dd, *J* = 8.0, 2.0 Hz, 1H), 7.34–7.30 (m, 2H), 7.15 (d, *J* = 8.0 Hz, 1H), 6.90–6.86 (m, 2H), 6.33 (br d, *J* = 7.5 Hz, 1H), 5.29 (quin, *J* = 7.0 Hz, 1H), 3.79 (s, 3H), 2.28 (overlapping s, 6H), 1.58 (d, *J* = 7.0 Hz, 3H).

^{13}C NMR (101 MHz, $CDCl_3$): δ = 166.7, 159.0, 140.5, 137.0, 135.6, 132.3, 129.8, 128.3, 127.6, 124.4, 114.1, 55.4, 48.6, 21.7, 19.9, 19.8.

HRMS (ESI) m/z calc: [M+H]+ ($C_{18}H_{22}NO_2$) 284.1645; measured: 284.1635 = 3.5 ppm difference

IR (FTIR, cm^{-1}): ν_{max} =3299 (N–H), 2971, 2939, 2832, 2049, 1879, 1775, 1640 (C=O), 1533, 1515, 1456, 1254, 1182, 1034, 831, 750.

*N-(1-(4-Methoxyphenyl)ethyl)-3,5-dimethylbenzamide (**3s**)*

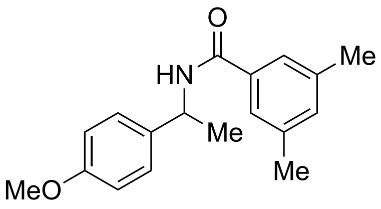

Title compound **3s** was prepared using the general procedure using 1-ethyl-4-methoxybenzene (28 μL, 0.2 mmol) and 3,5-dimethylbenzamide (60 mg, 0.4 mmol). After column chromatography on silica gel eluting with 20% EtOAc in *n*-pentane gave amide **3s** (19 mg, 34%) as a brown oil.

R_f = 0.17 (1:4 EtOAc–petrol)

^{1}H NMR (400 MHz, $CDCl_3$): δ = 7.37–7.30 (m, 4H), 7.11 (s, 1H), 6.92–6.86 (m, 2H), 6.25 (br d, *J* = 7.5 Hz, 1H), 5.29 (quin, *J* = 7.0 Hz, 1H), 3.80 (s, 3H), 2.33 (overlapping s, 6H), 1.58 (d, *J* = 7.0 Hz, 3H).

^{13}C NMR (101 MHz, $CDCl_3$): δ = 167.0, 159.0, 138.4, 135.5, 134.8, 133.1, 127.6, 124.8, 114.2, 55.4, 48.6, 21.7, 21.3.

HRMS (ESI) m/z calc: [M+H]+ ($C_{18}H_{22}NO_2$) 284.1645; measured: 284.1635 = 3.5 ppm difference

IR (FTIR, cm^{-1}): ν_{max} =3299 (N–H), 3052, 2971, 2926, 2832, 2054, 1888, 1735, 1636 (C=O), 1596, 1515, 1465, 1254, 1182, 1025, 867, 827, 764.

Failed Substrates and Limitations of Benzylic Amidation Reaction

Failed heteroaromatic benzylic substrates:

- Reactions were carried out using general procedure with benzamide.

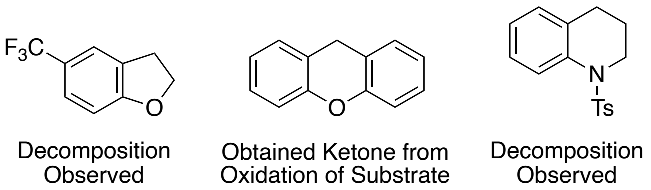

Figure S5

Failed tertiary C-H benzylic substrates:

- Reactions were carried out using general procedure with benzamide.

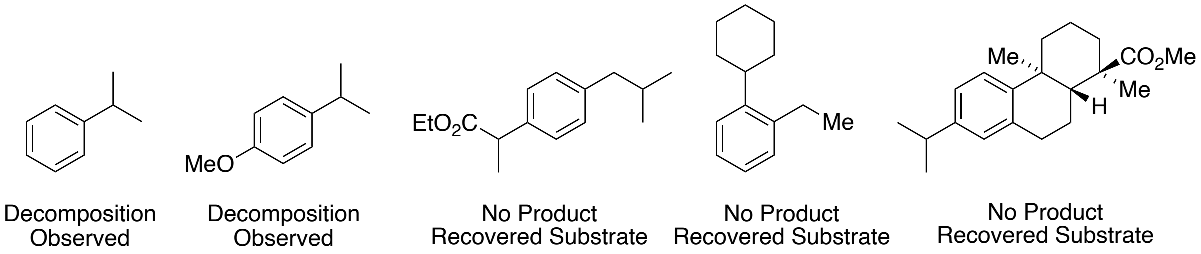

Figure S6

Failed alkylamide and secondary amide substrates:

- Reactions were carried out using general procedure with *t*-butyl-3-ethylbenzene.

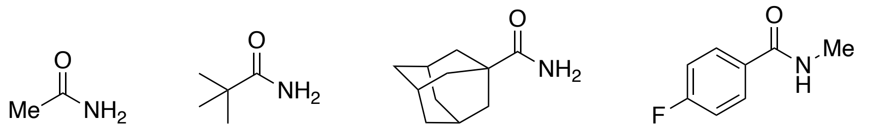

No product formation observed in all reactions *via* ^{1}H-NMR analysis of crude product

Figure S7

Addition of HFIP to benzylic amidation reaction:

- The reaction below was performed to access any potential benefit from adding HFIP to the reaction. In summary, no desired product was formed.

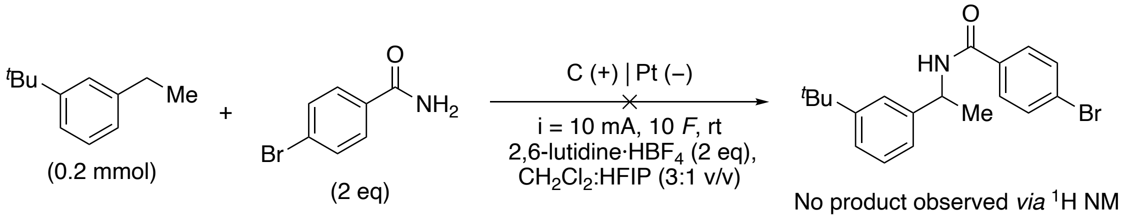

No product observed *via* ^{1}H NMR.
Obtained ketone from
oxidation of starting material.

Addition of 2,6-Lutidine and $TBAPF_6$ to benzylic amidation reaction:

- The reaction below was performed to access any potential benefit from using 2,6-Lutidine and $TBAPF_6$ to the reaction. In summary, no desired product was formed.

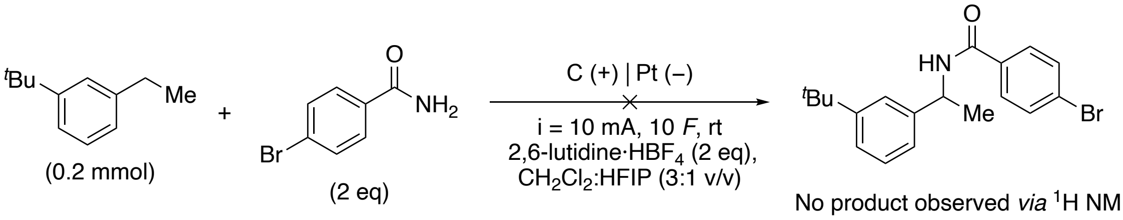

No product observed *via* ^{1}H NMR.
Obtained ketone from
oxidation of starting material.

GCMS of Crude Product from Optimised Reaction Conditions:

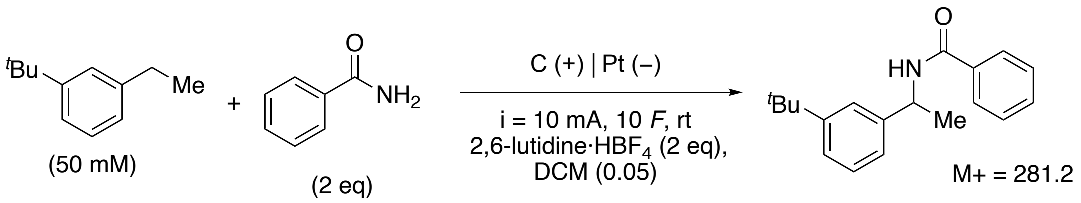

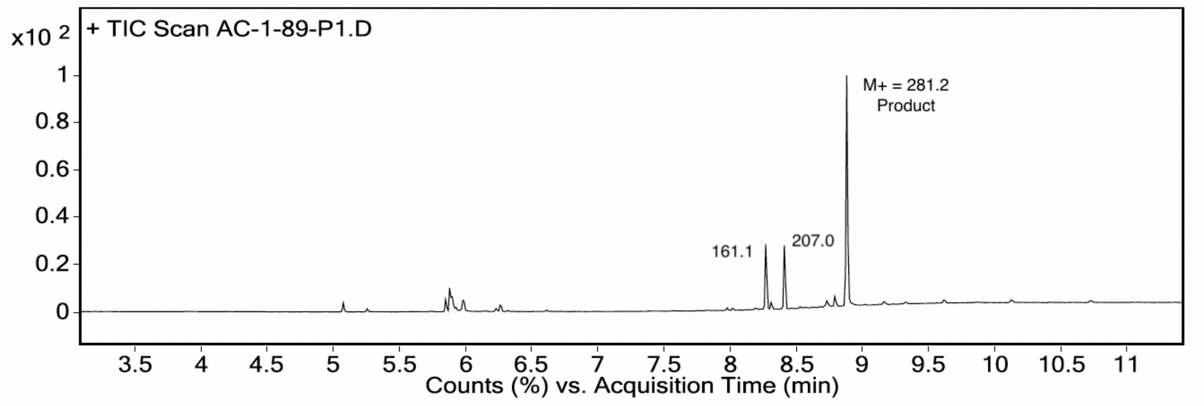

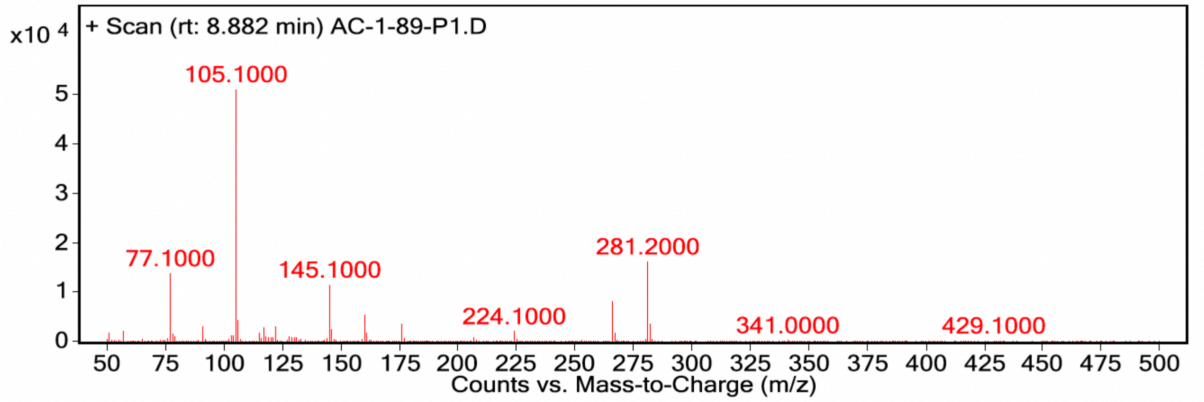

Figure S8

Electrochemical Flow Procedure

General Information

The ElectroVortex used for these reactions was set-up with a graphite outer (anode) and a stainless-steel rotor (cathode) and one of the three different gap sizes (0.5, 1.0 and 1.5 mm giving reaction volumes of 4.6 mL, 9.4 mL and 14.4 mL respectively). The current was supplied to the electrodes by a 720 W Keithley power supply unit (PSU) and the rotation of the rod was provided by a BLM260HP brushless motor. Both the inlet and outlet pumps are ColePalmer MasterFlex L/S peristaltic pumps using PTFE pump heads connected with 1/8” and 1/16” OD PFA tubing. The cooling is provided by a Haake F3 cooler using a mixture of IPA and H_2O (1:1).

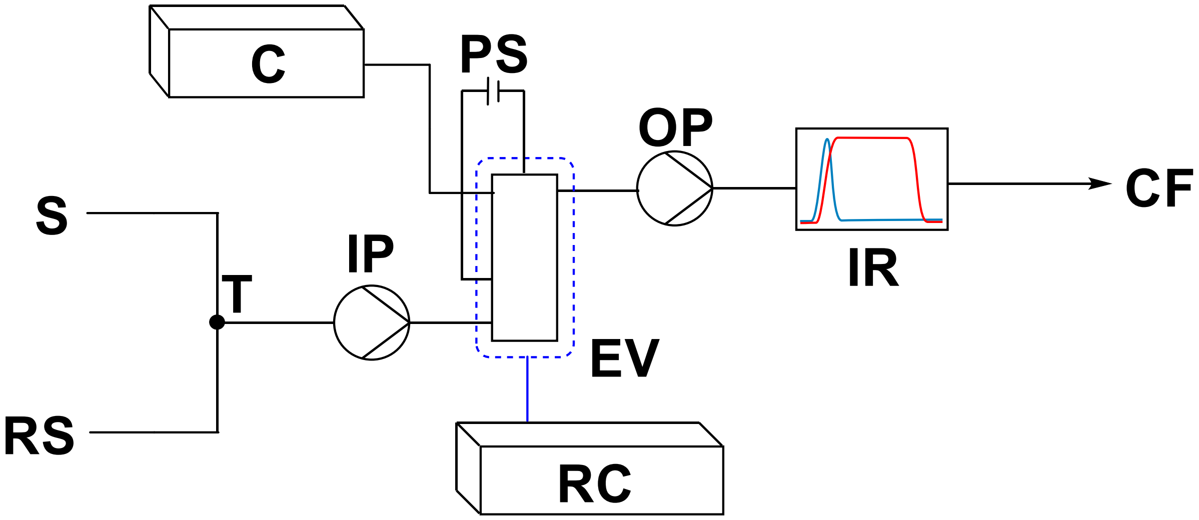

Figure S9: Overview of the continuous flow set-up of the ElectroVortex: S = Solvent; RS = Reaction Solution; T = T-Piece; IP = Inlet Pump; C = Controller; PS = Power Suply; EV = ElectroVortex; RC = Recirculating Chiller; OP = Outlet Pump; IR = Online FTIR and CF = Collection Flask.

General Procedure for using the ElectroVortex Reactor

Both the inlet and outlet pumps are calibrated to the desired flow rate using DCM and the chiller is set to 20 °C (1:1 IPA-MeOH). A flask containing DCM is attached and the inlet and outlet pumps are set to 5 mL min^{-1}, the rotor is then turned to the desired rotation and the reactor is allowed to flush with neat DCM for 10 minutes. A flask containing the reaction solution is then connected and the inlet pump is set to the desired flow rate. The solution is then left pumping until the FTIR monitoring indicates that a steady-state concentration is achieved within the system monitored by IR. The power supply is then turned on and set to the desired current using the constant current mode. A sample is then collected for analysis after steady-state has been observed. After all experiments are completed, the system was flushed with clean DCM for 10 minutes.

Preparation of indane and 4-ethylanisole reaction solutions:

In a 100 mL volumetric flask was weighed benzamide (3.03 g, 25.0 mmol), 2,6-lutidine·HBF_4 (4.87 g, 25.0 mmol) and tetrabutylammonium tetrafluoroborate (8.23 g, 25.0 mmol) followed by the addition of DCM (50 mL). The solids were dissolved using a sonicator and subsequently either indane (6.13 mL, 50.0 mmol) or 4-ethylanisole (6.30 mL, 50.0 mmol) were then added and the flask filled to 100 mL using DCM. The solution was then degassed by bubbling argon for 30 minutes. General procedure was then followed to carry out all subsequent flow experiments.

IR data analysis

An Multivariate Curve Resolution (MCR) model was constructed using MATLAB and PLS toolbox from Eigenvector, with pure calibration solutions with a range of concentrations (0 to 0.5 M). The subsequent MCR model was applied to reaction data, which allowed the reaction concentrations to be estimated as the reaction proceeded. 2D-COS analysis was performed using in-house MATLAB scripts developed following the analysis previously described.[5]

^{1}H, ^{13}C and ^{19}F NMR Spectra

Compound **3a** ^{1}H NMR (400 MHz, $CDCl_3$)

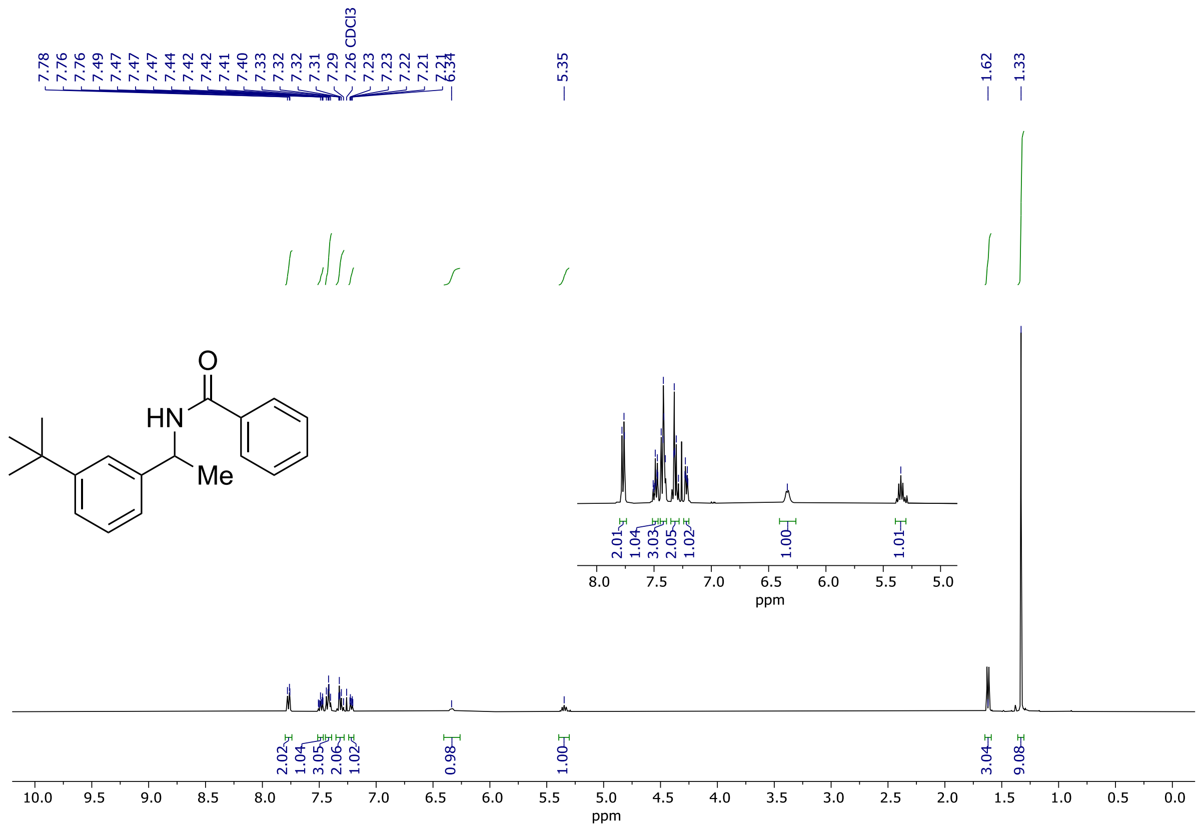

Compound **3a** ^{13}C NMR (101 MHz, $CDCl_3$)

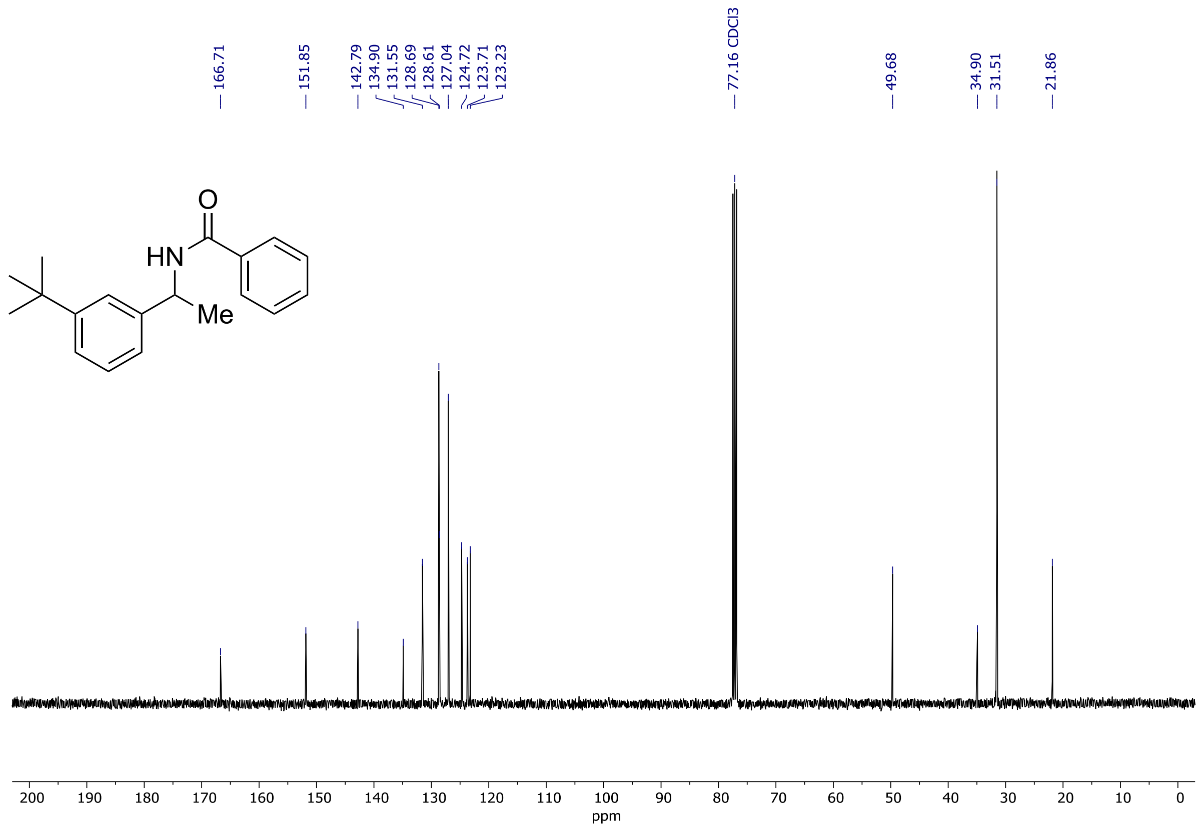

Compound **3b** ^{1}H NMR (400 MHz, $CDCl_3$)

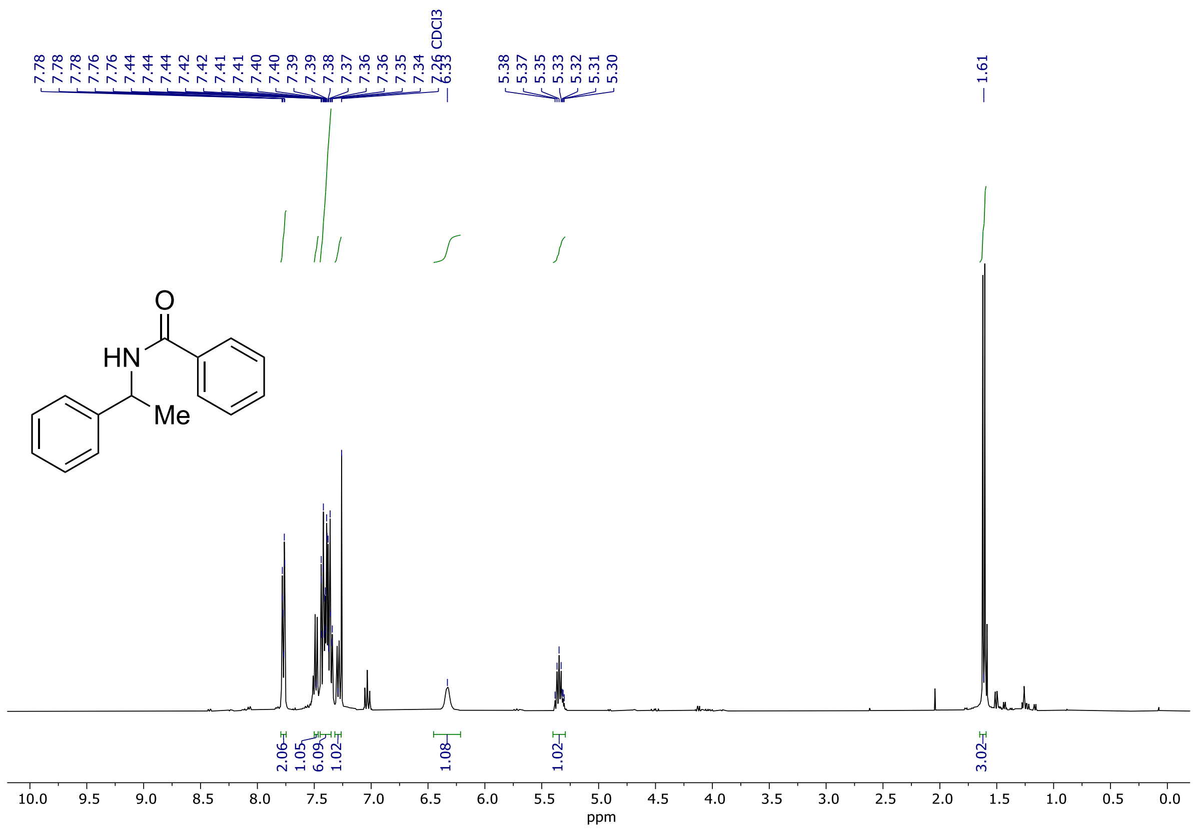

Compound **3b** ^{13}C NMR (101 MHz, $CDCl_3$)

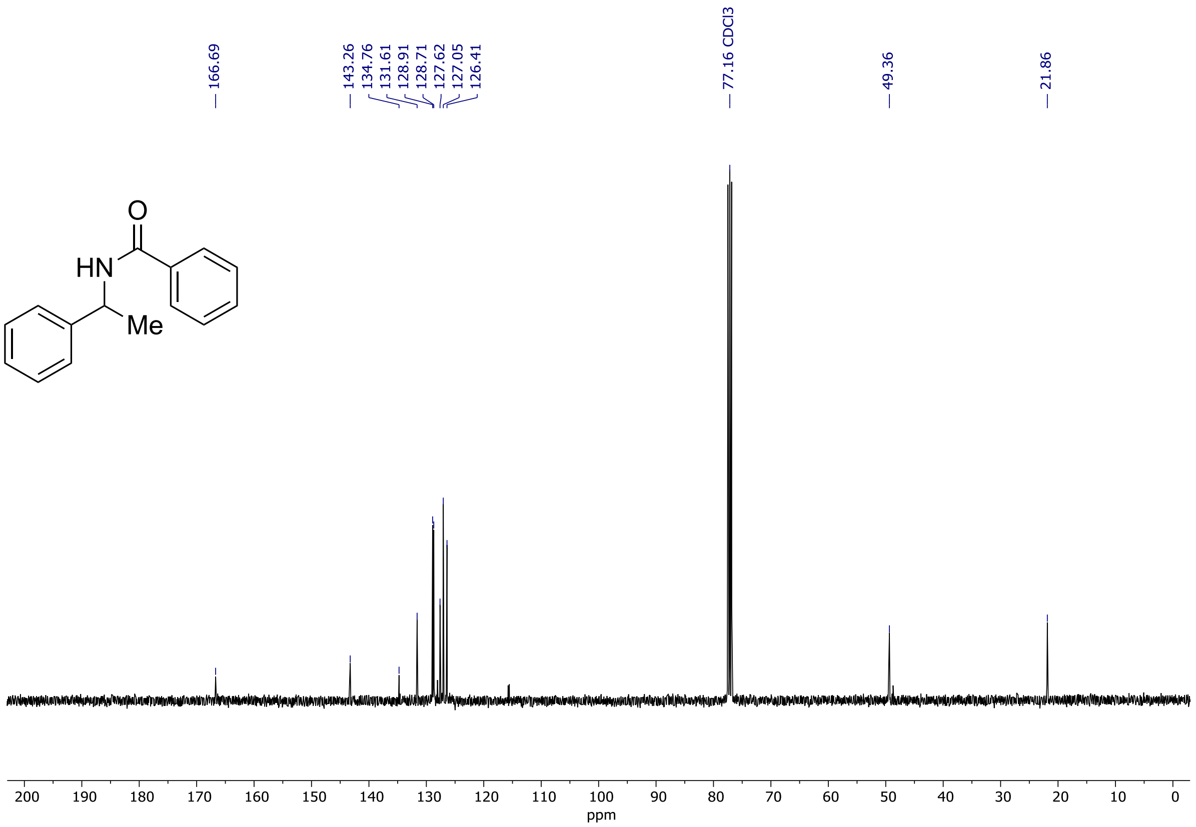

Compound **3c** ^{1}H NMR (400 MHz, $CDCl_3$)

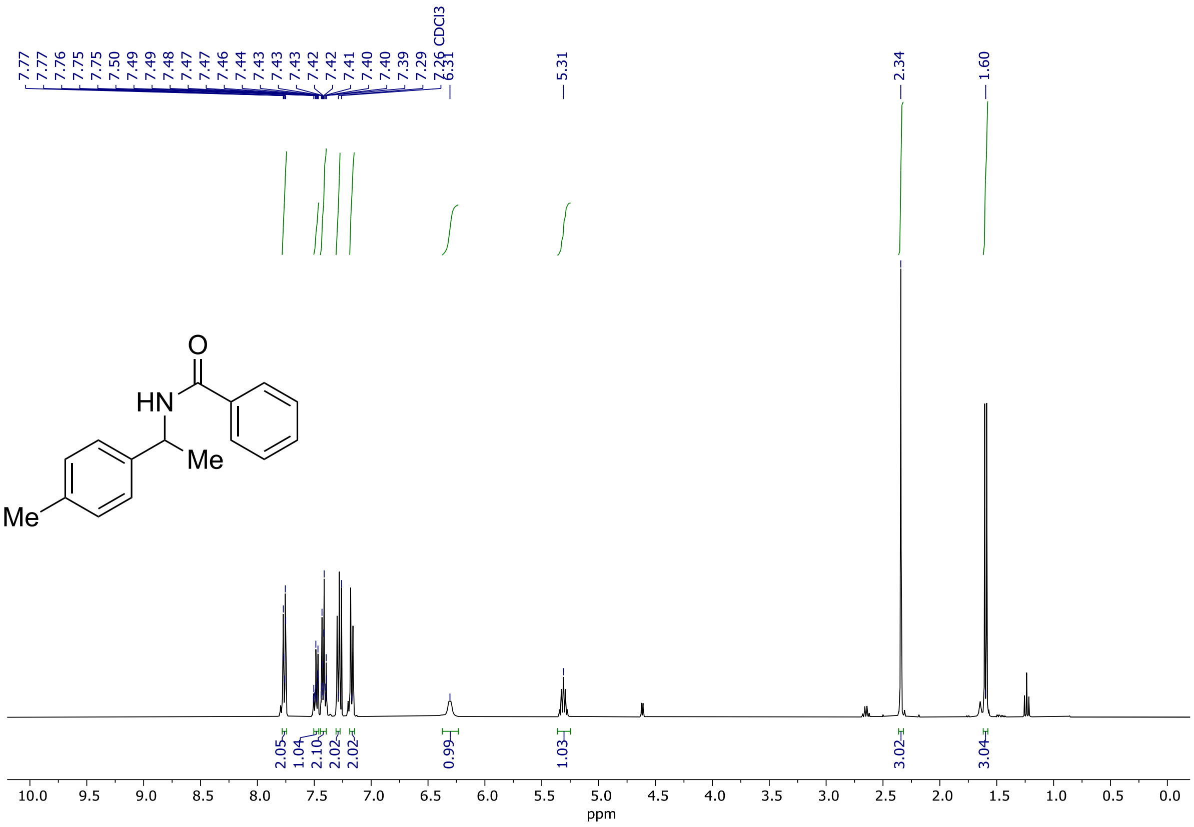

Compound **3c** ^{13}C NMR (101 MHz, $CDCl_3$)

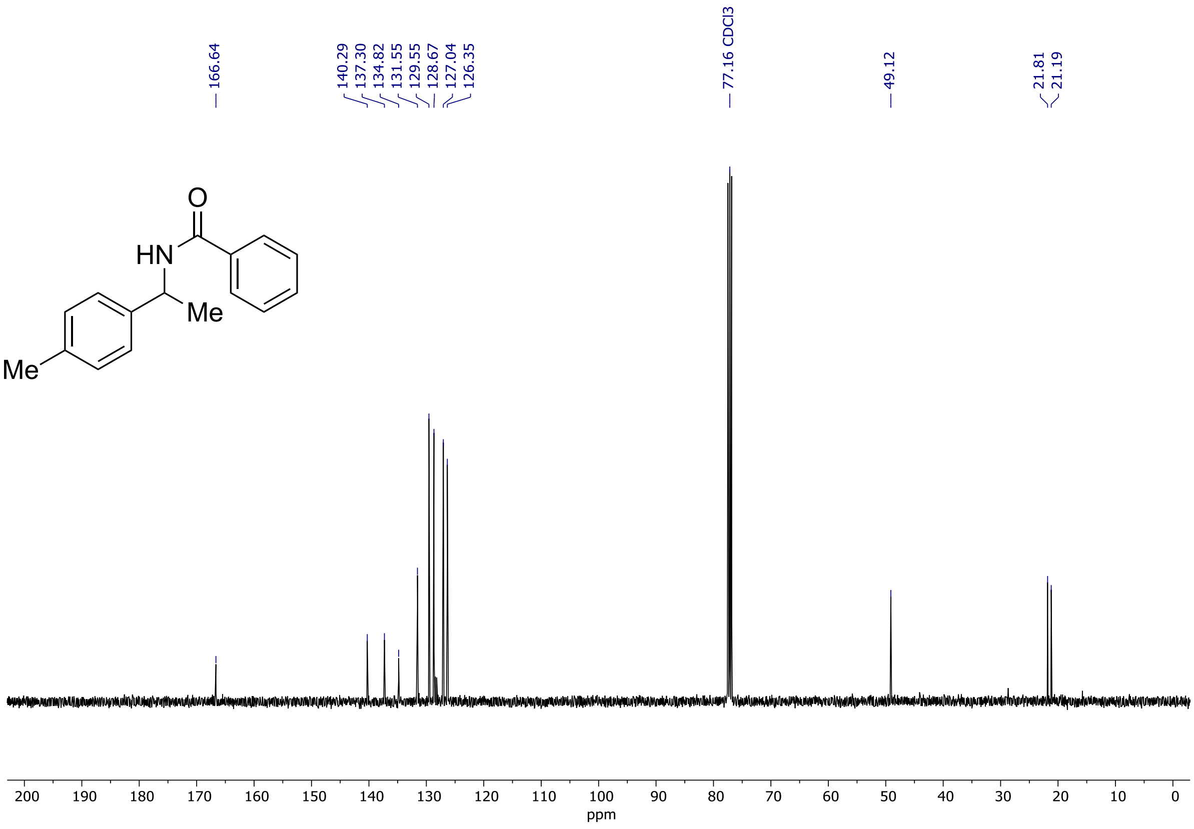

Compound **3d** ^{1}H NMR (400 MHz, $CDCl_3$)

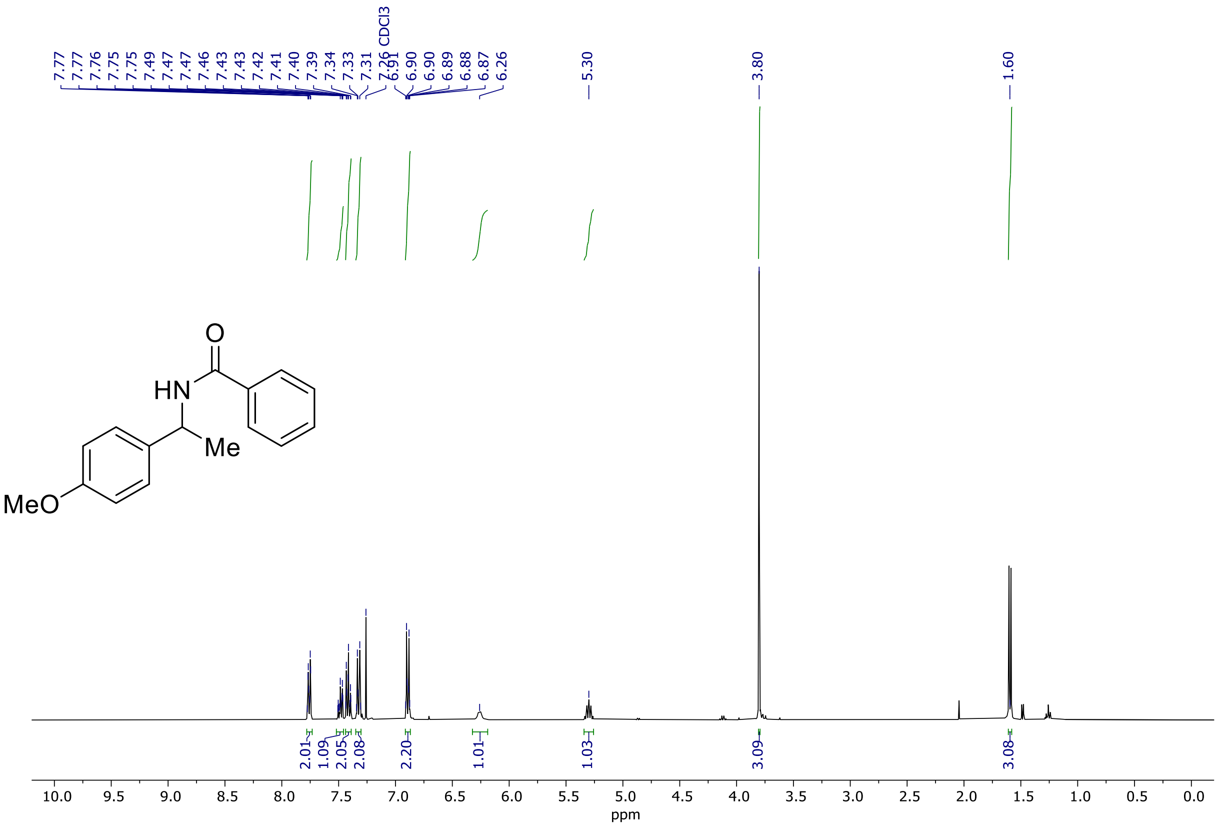

Compound **3d** ^{13}C NMR (101 MHz, $CDCl_3$)

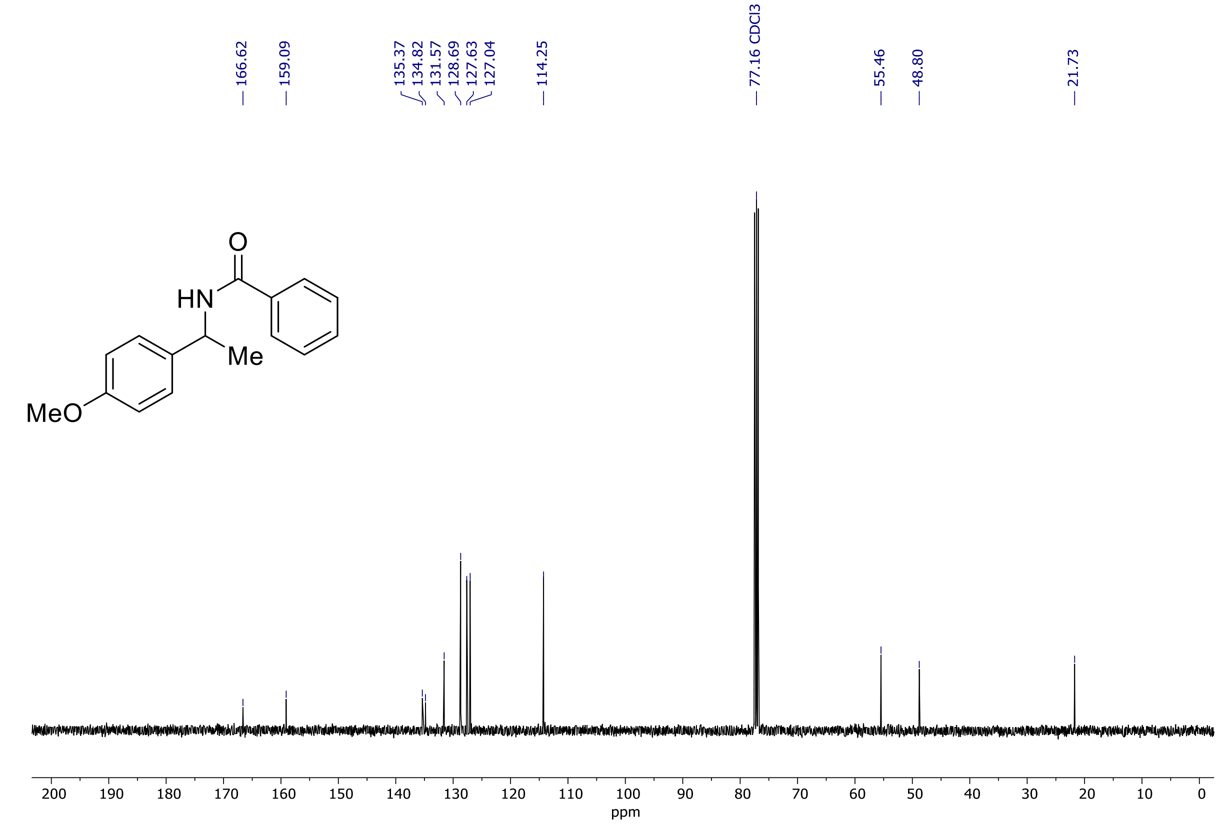

Compound **3e** ^{1}H NMR (400 MHz, $CDCl_3$)

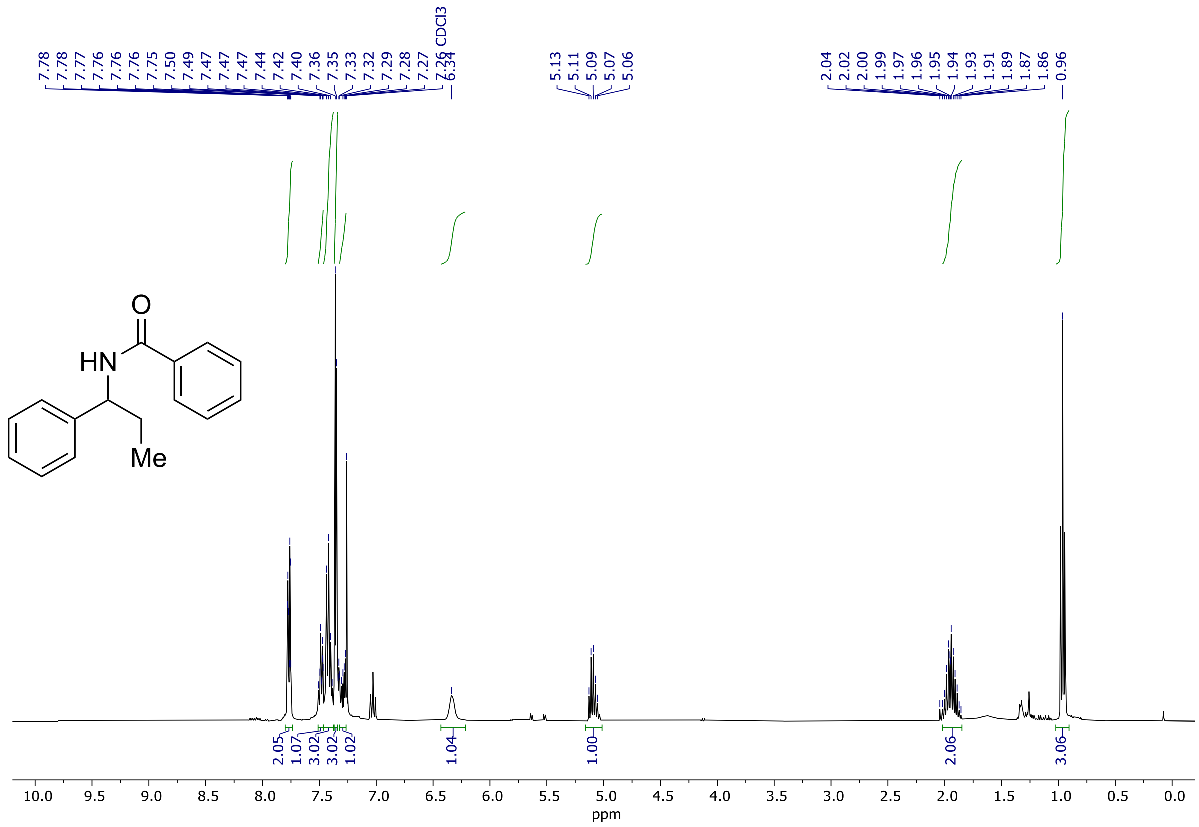

Compound **3e** ^{13}C NMR (101 MHz, $CDCl_3$)

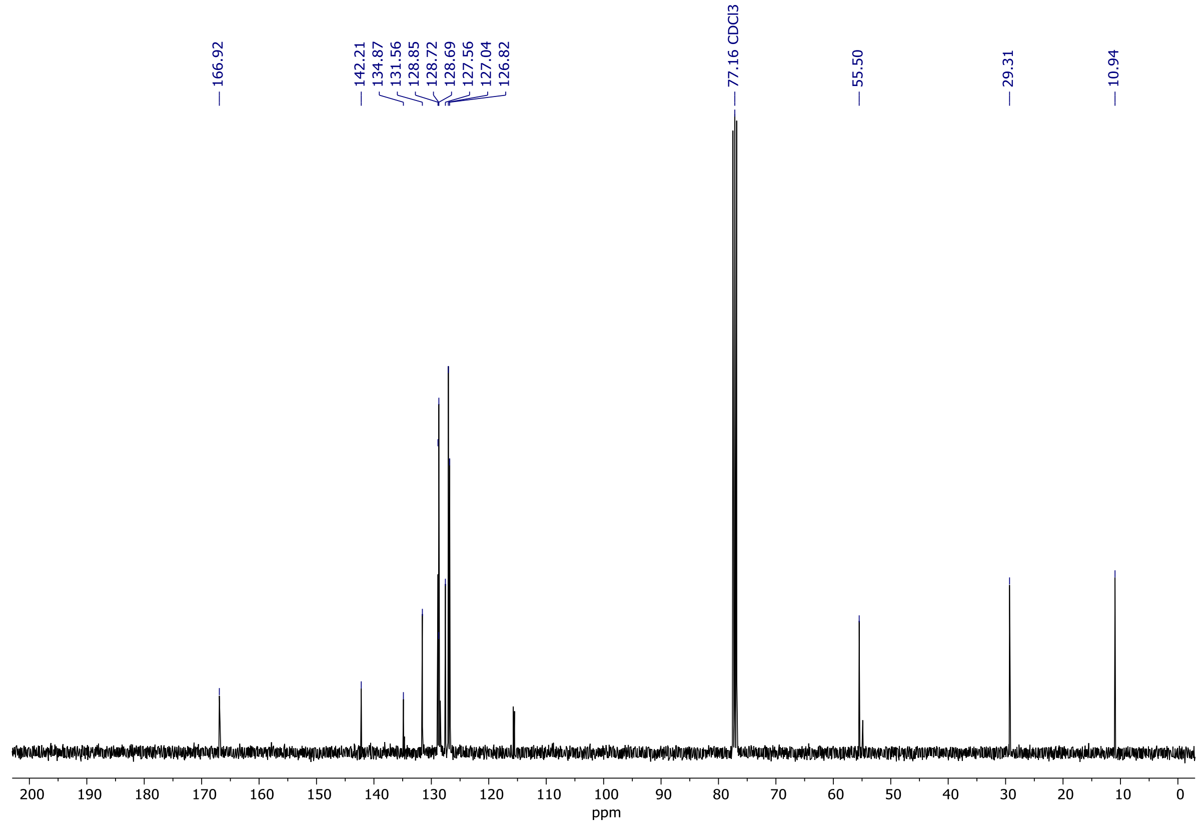

Compound **3f** ^1H NMR (400 MHz, $CDCl_3$)

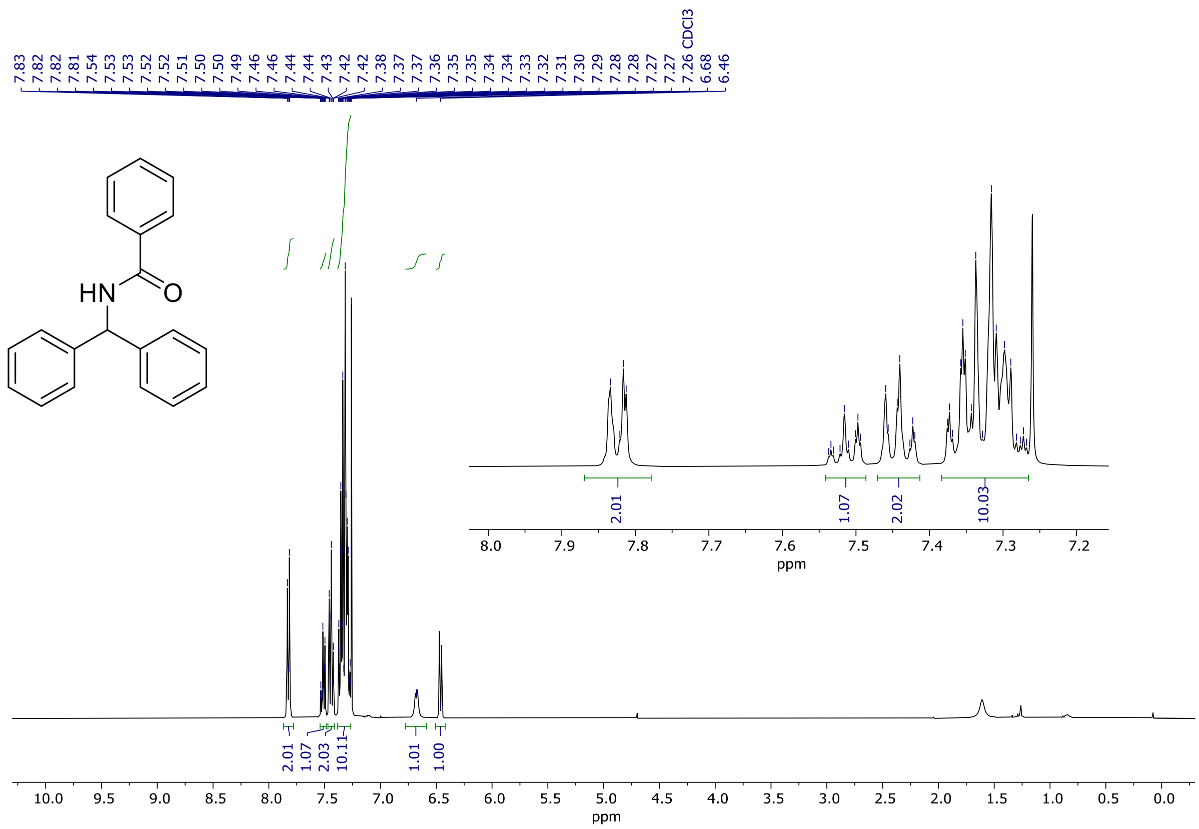

Compound **3f** ^{13}C NMR (101 MHz, $CDCl_3$)

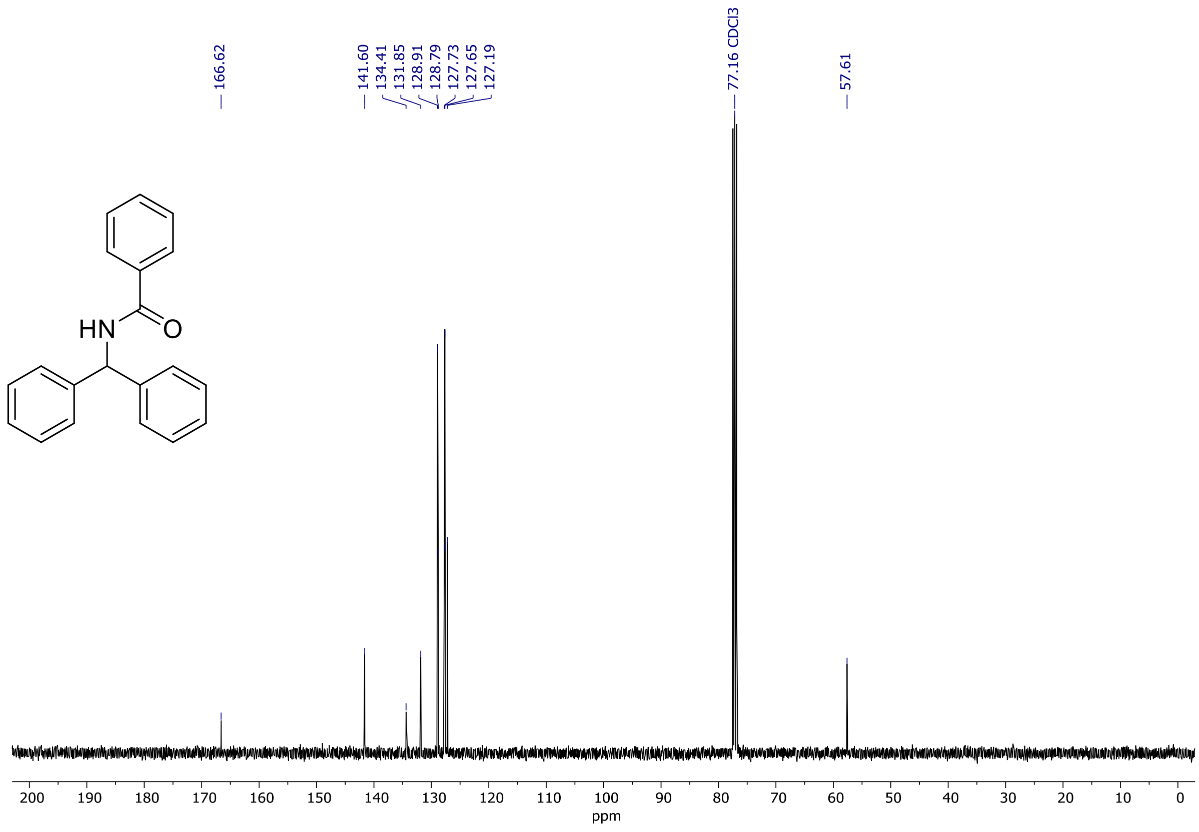

Compound **3g** ^1H NMR (400 MHz, $CDCl_3$)

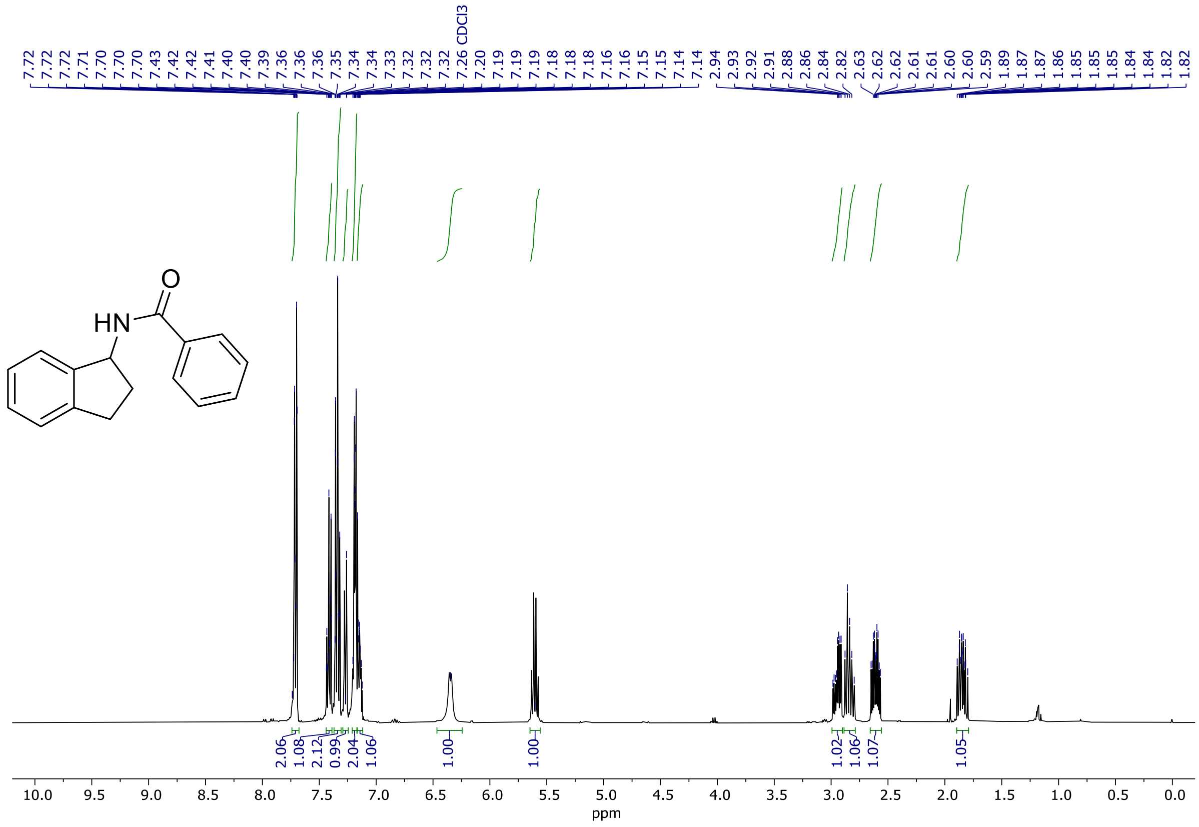

Compound **3g** ^{13}C NMR (101 MHz, $CDCl_3$)

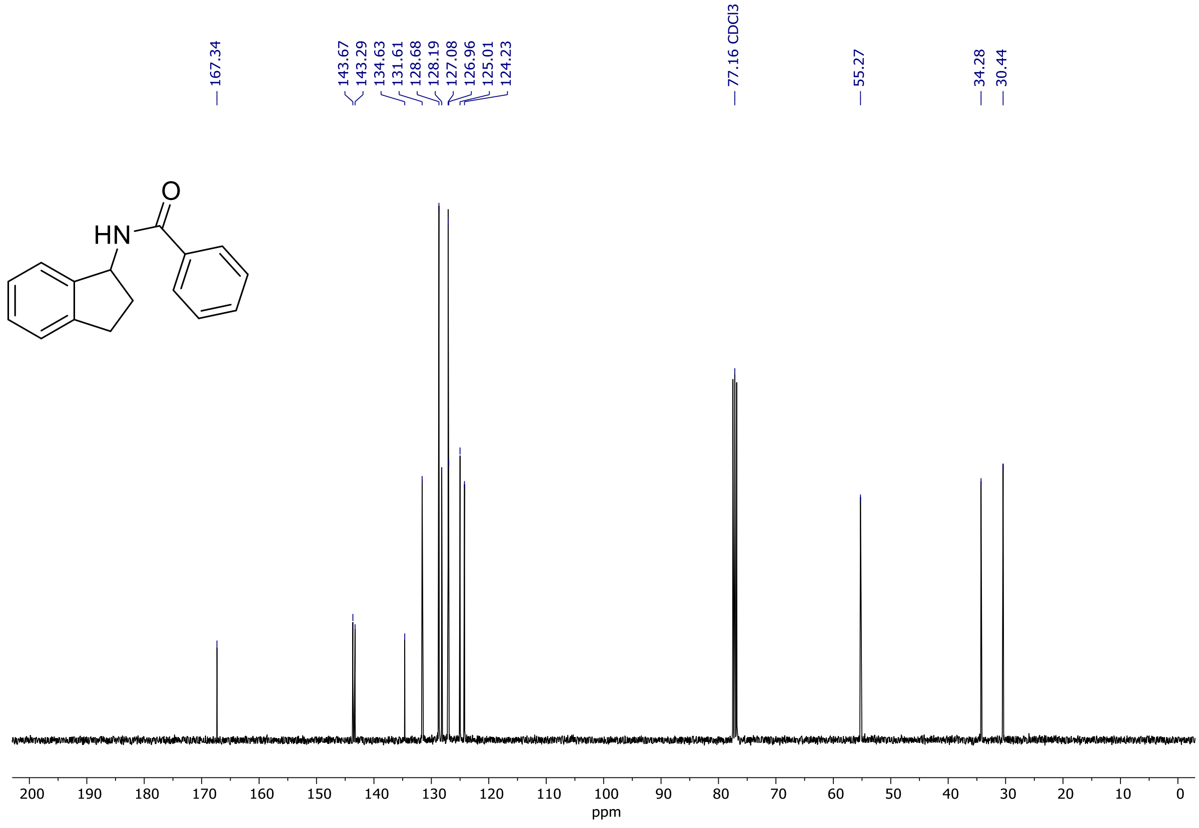

Compound **3h** ^{1}H NMR (400 MHz, $CDCl_3$)

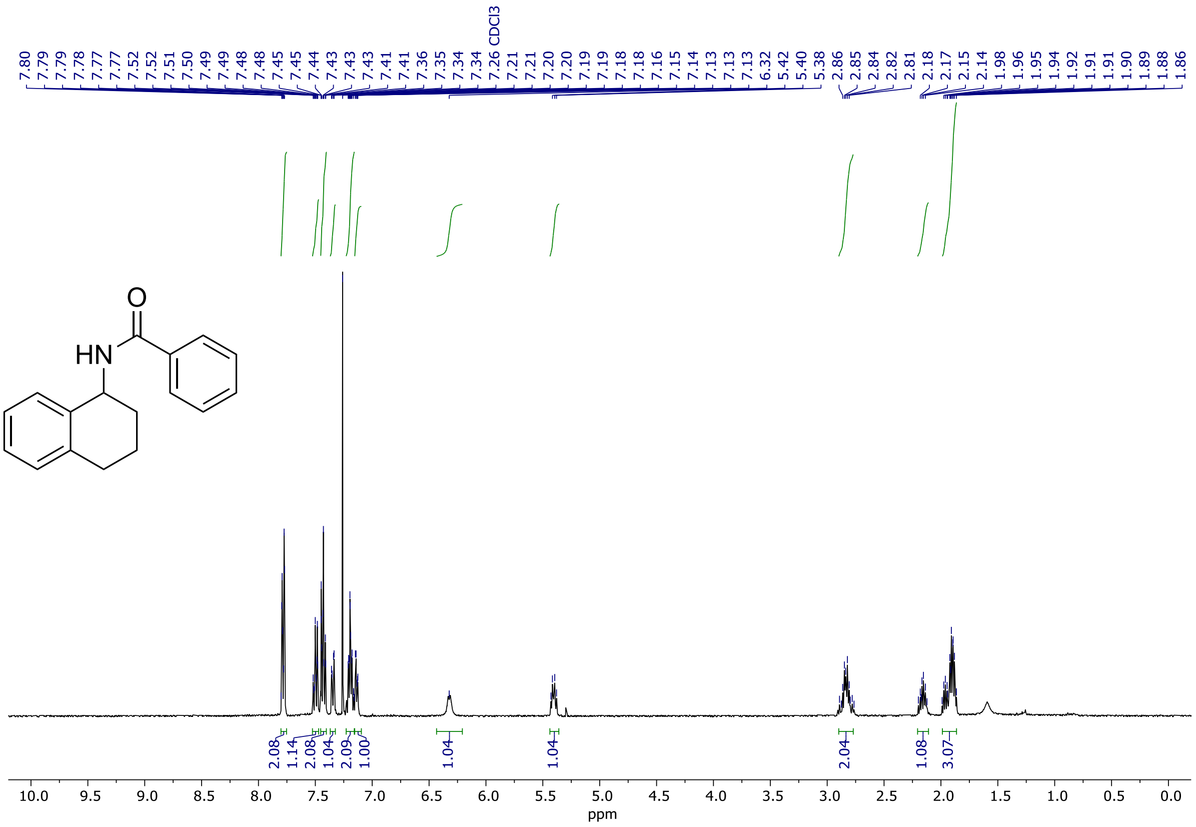

Compound **3h** ^{13}C NMR (101 MHz, $CDCl_3$)

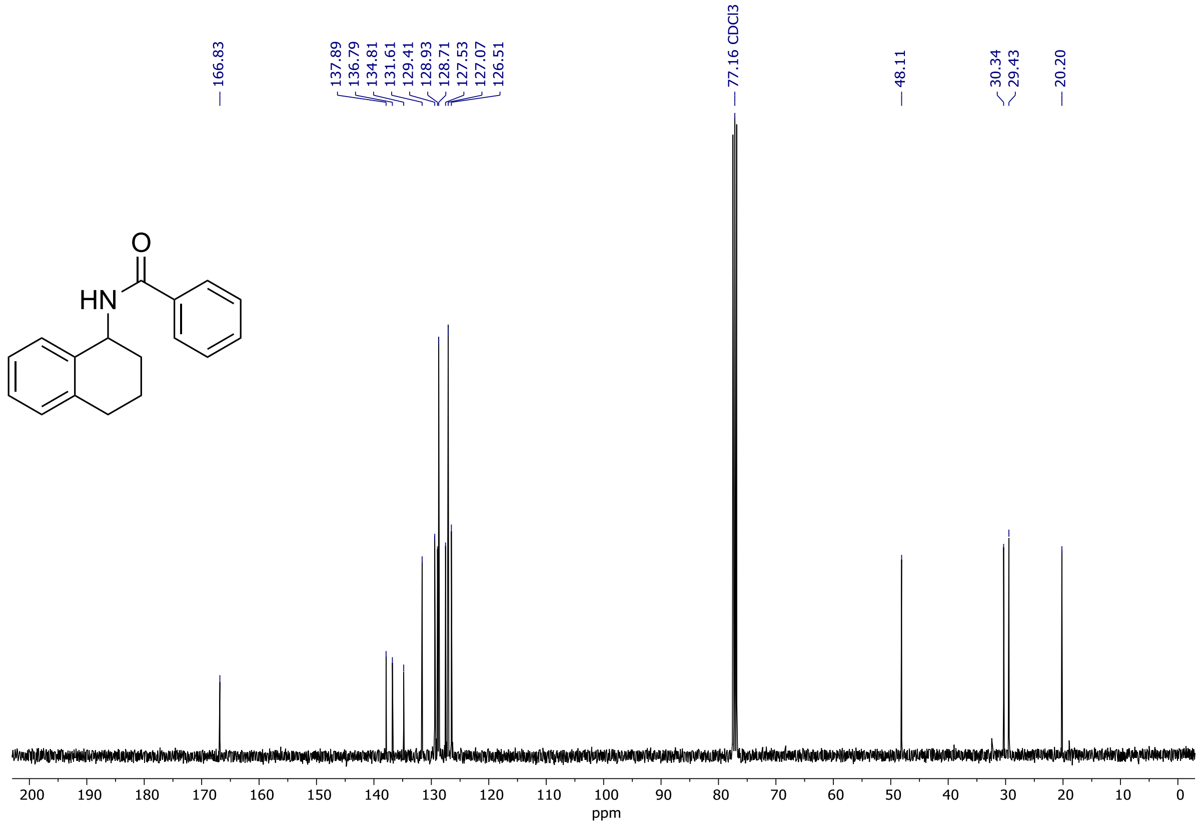

Compound **3i** ^{1}H NMR (400 MHz, $CDCl_3$)

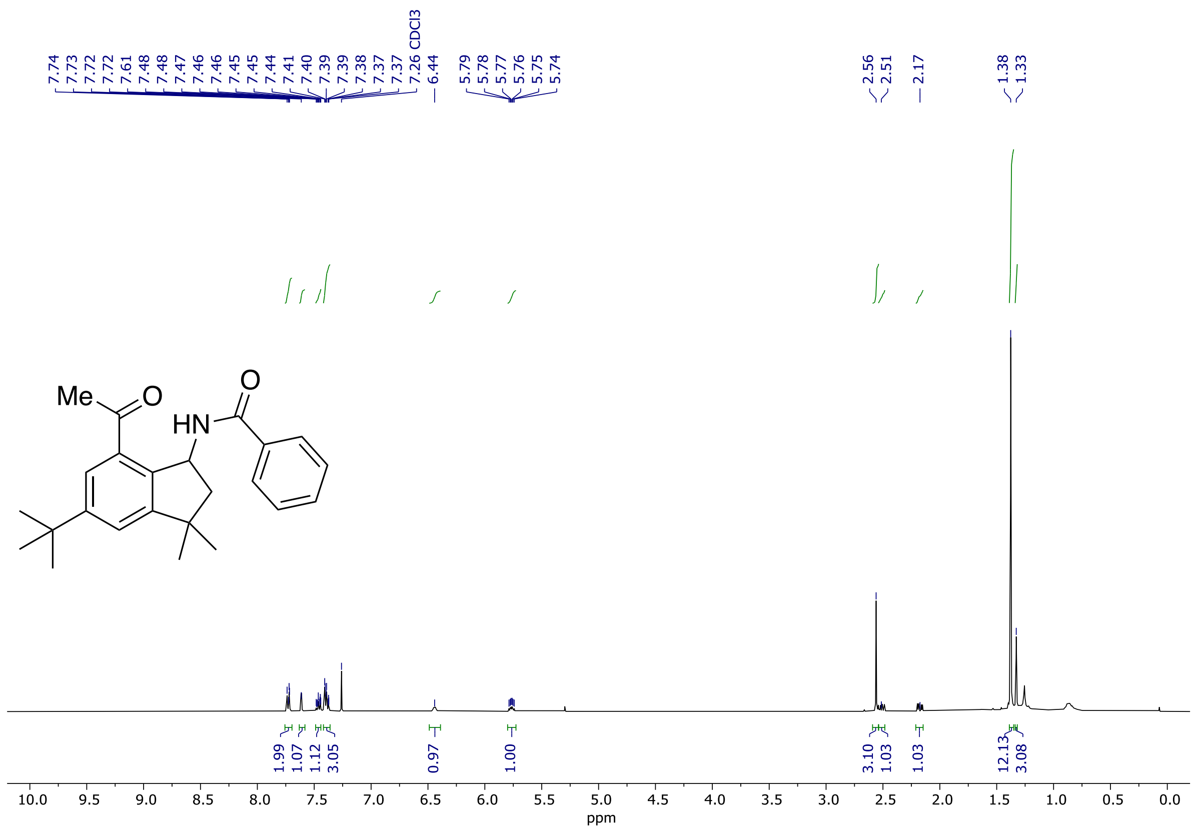

Compound **3i** ^{13}C NMR (101 MHz, $CDCl_3$)

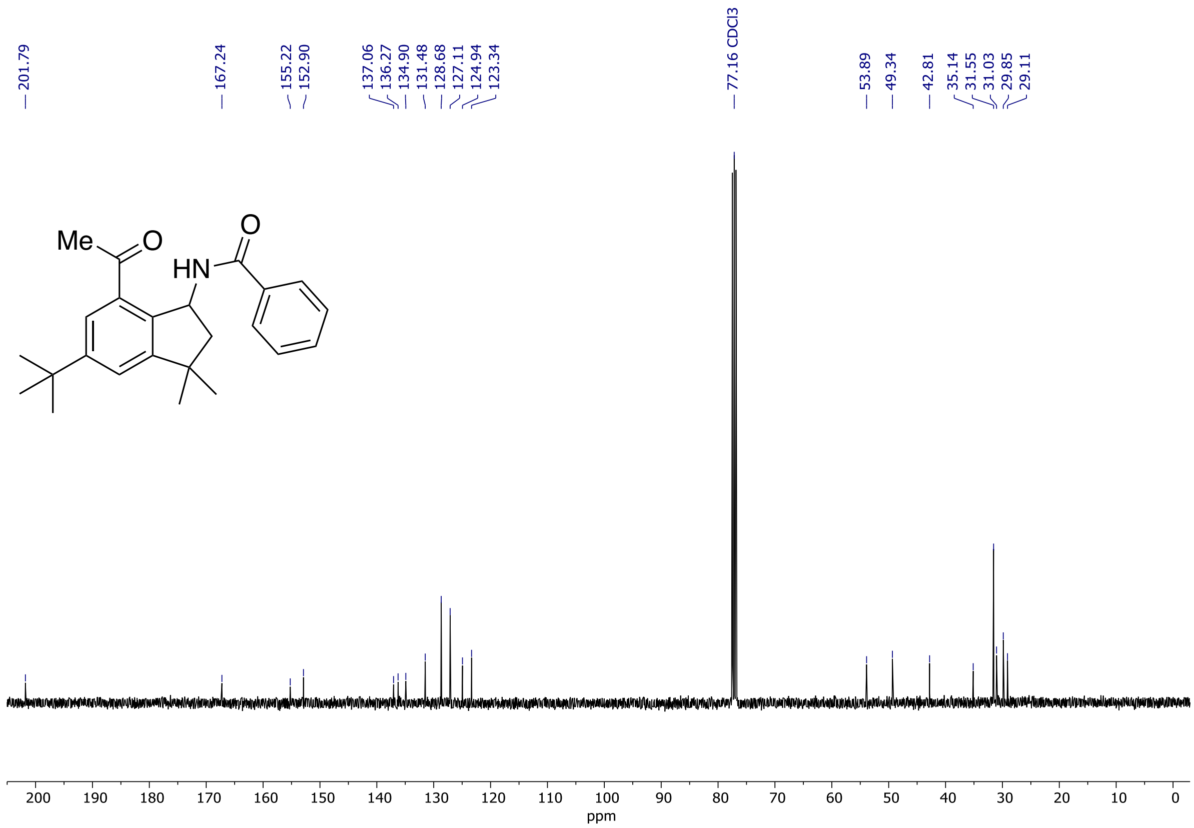

Compound **3j** ^{1}H NMR (400 MHz, $CDCl_3$)

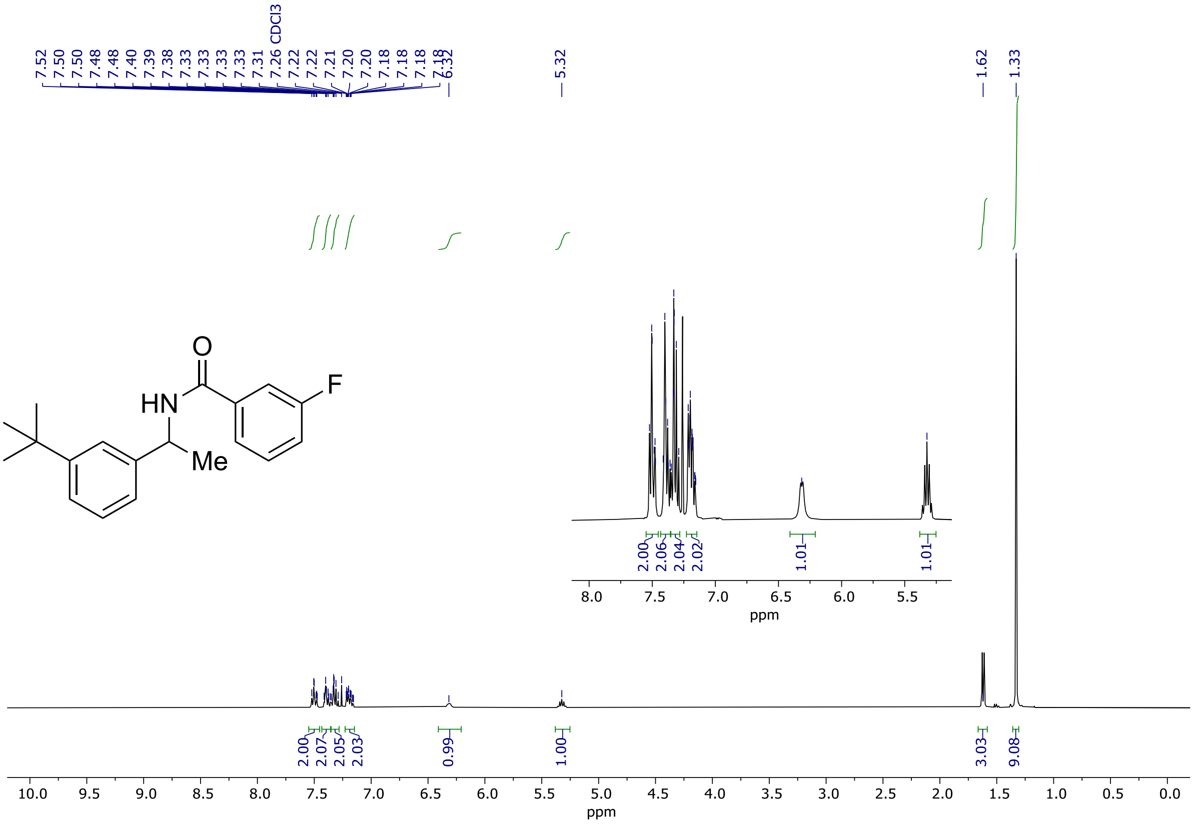

Compound **3j** ^{13}C NMR (101 MHz, $CDCl_3$)

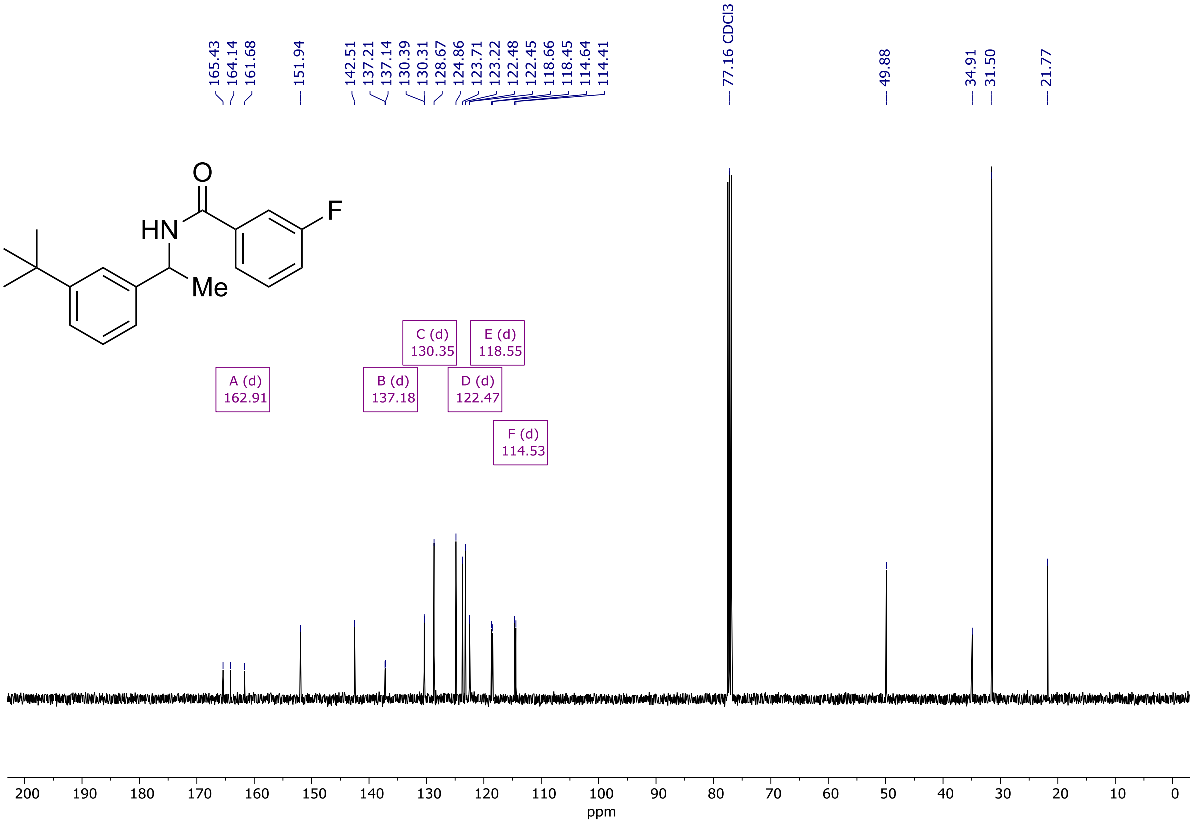

Compound **3j** ^{19}F NMR (377 MHz, $CDCl_3$)

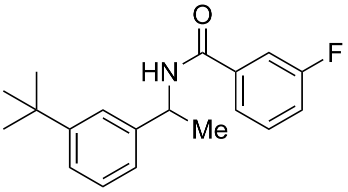

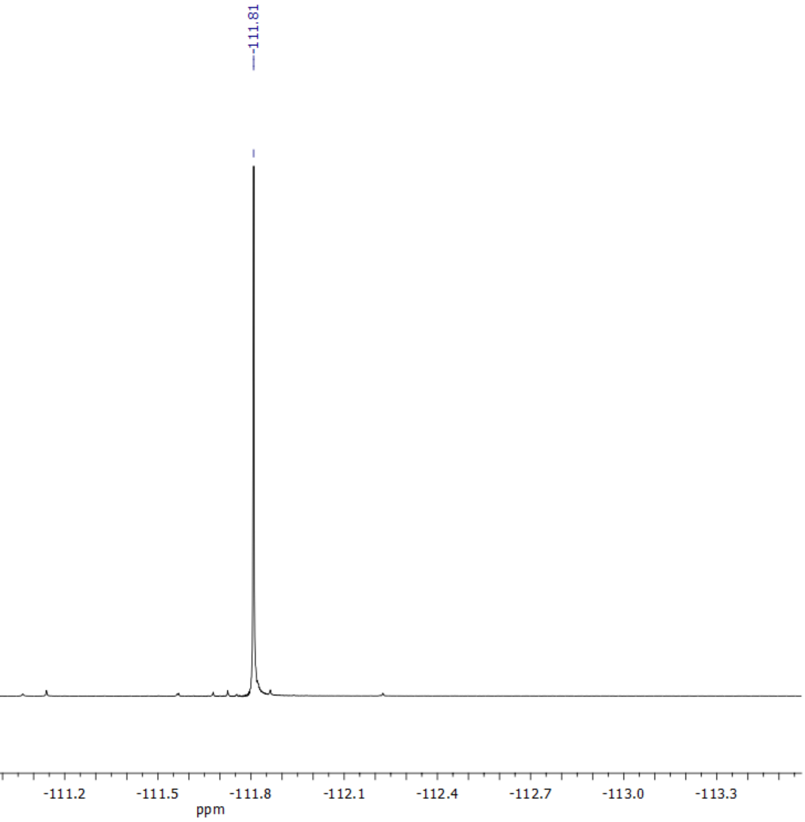

Compound **3k** ^{1}H NMR (400 MHz, $CDCl_3$)

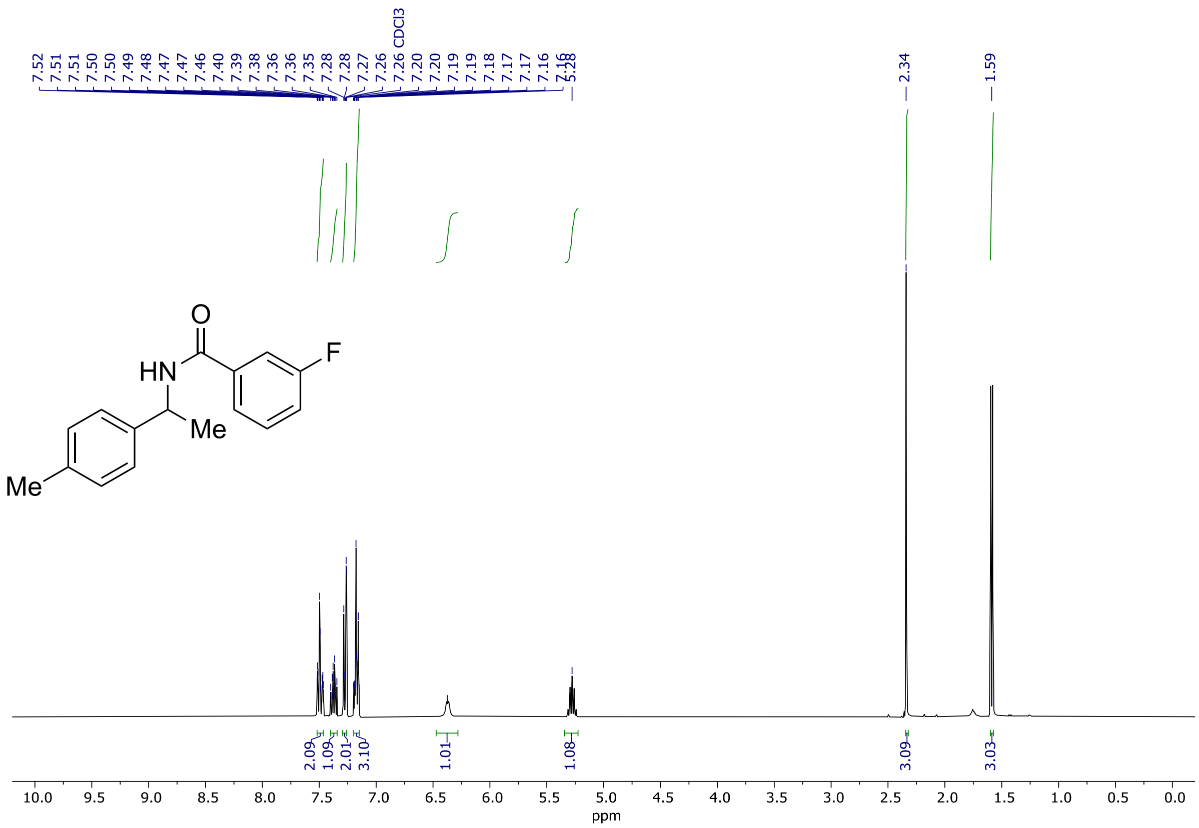

Compound **3k** ^{13}C NMR (101 MHz, $CDCl_3$)

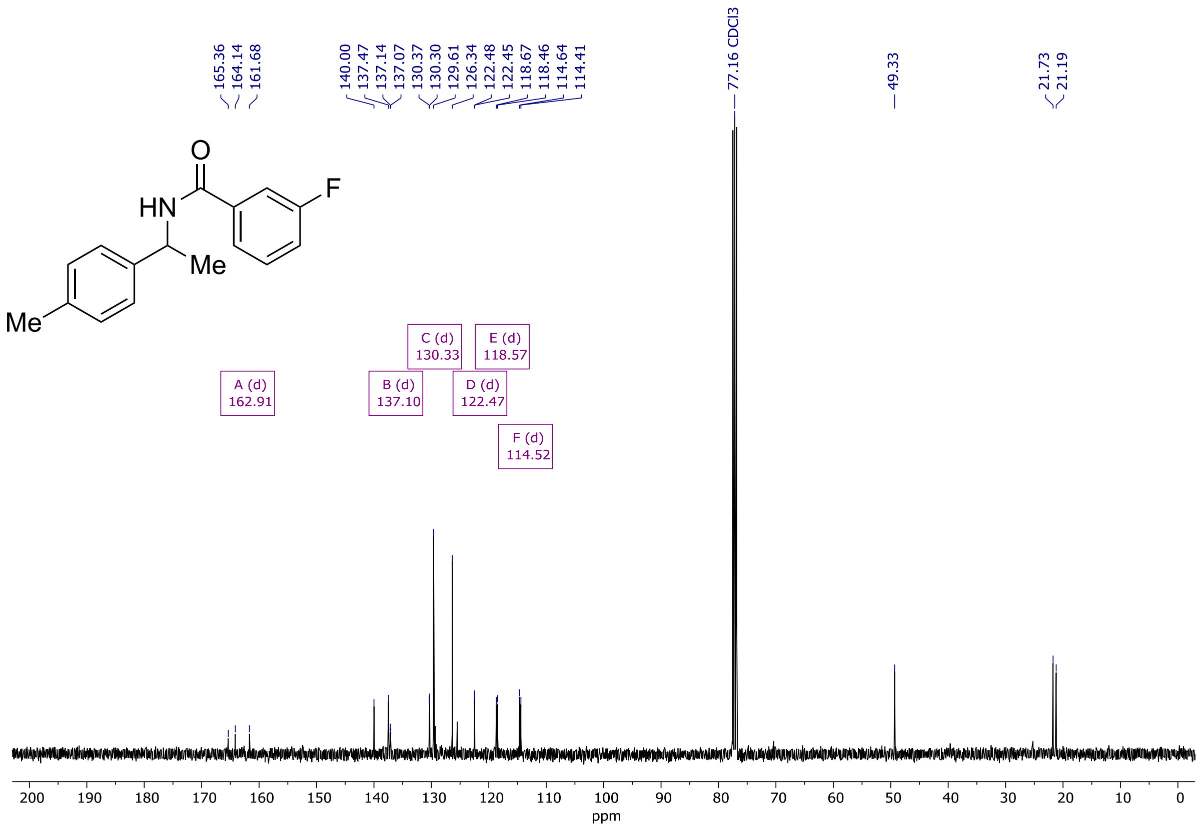

Compound **3k** ^{19}F NMR (377 MHz, $CDCl_3$)

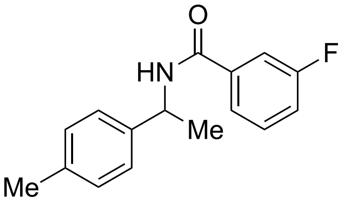

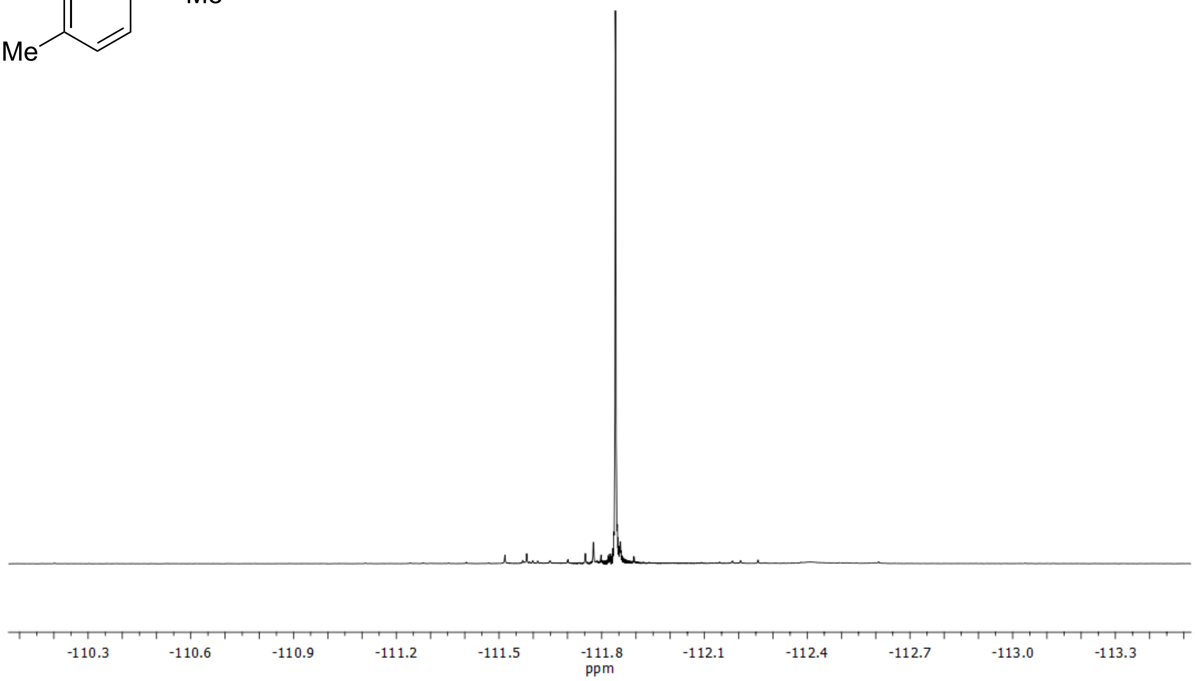

Compound **3l** ^{1}H NMR (400 MHz, $CDCl_3$)

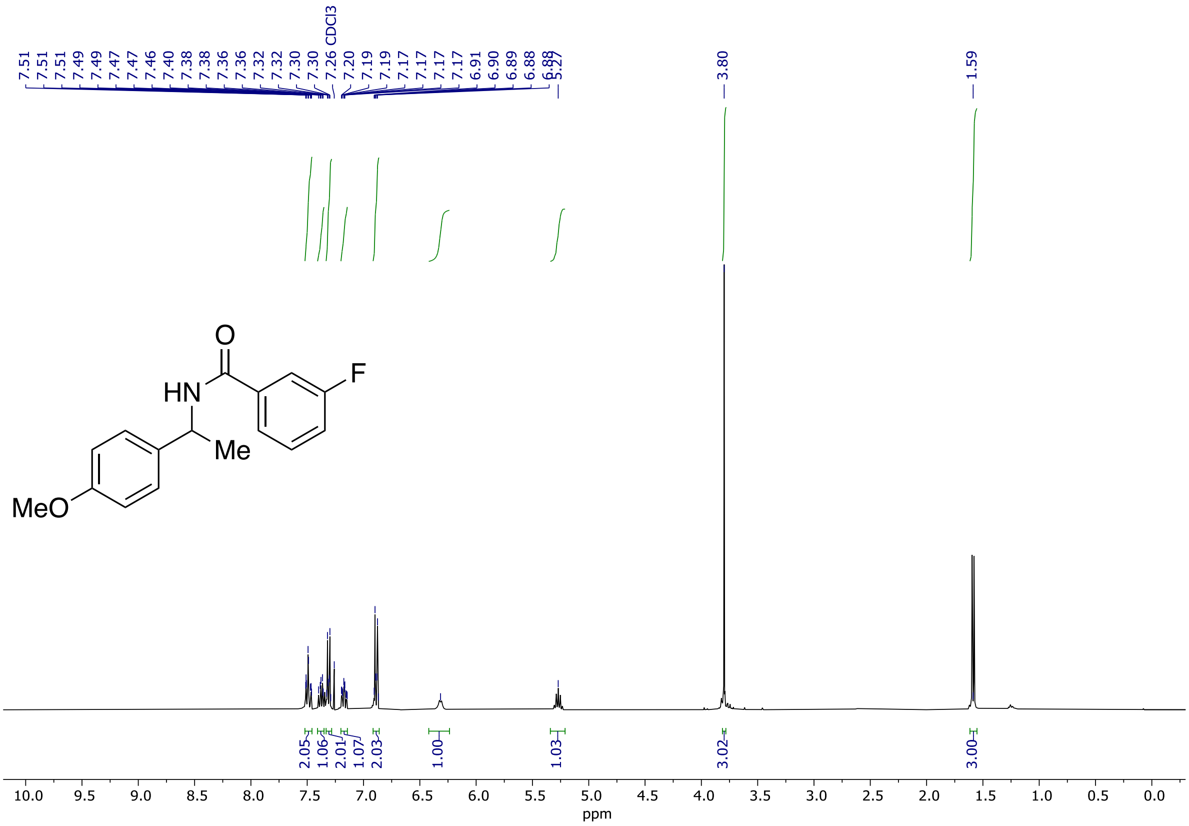

Compound **3l** ^{13}C NMR (101 MHz, $CDCl_3$)

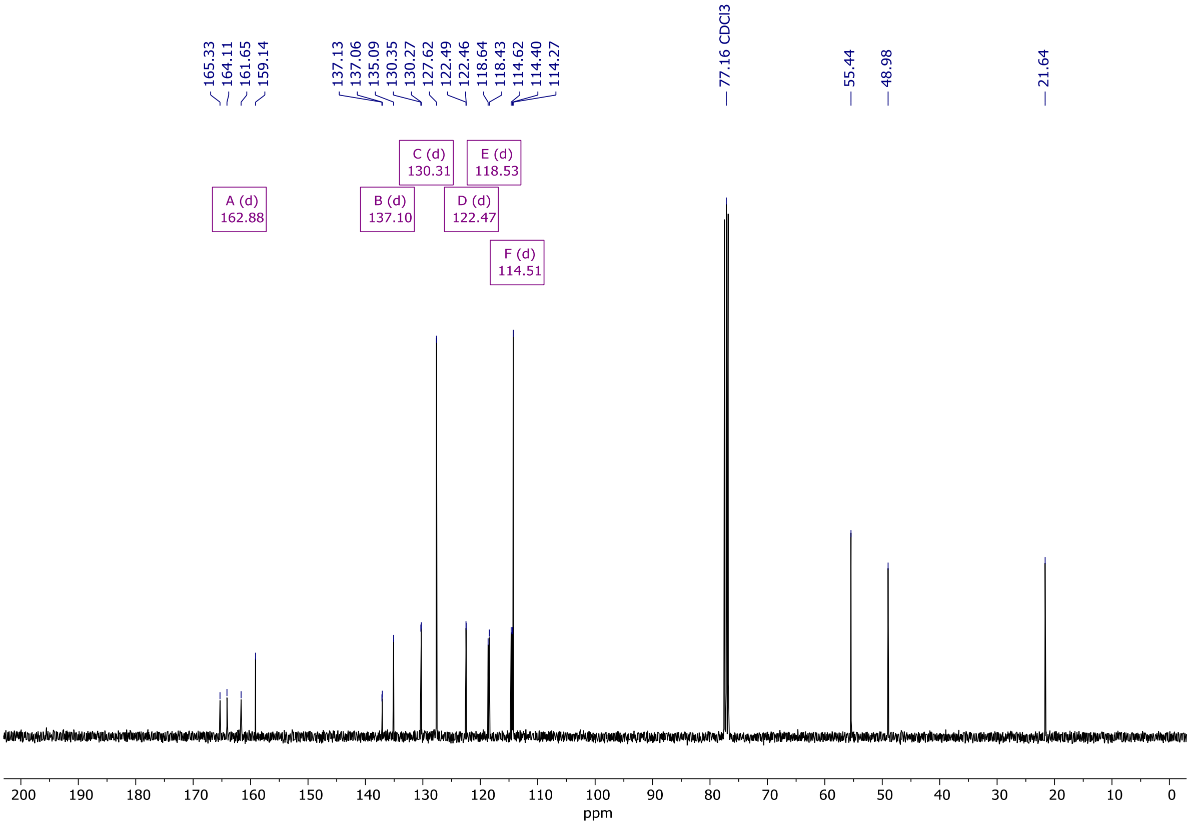

Compound **3l** ^{19}F NMR (377 MHz, $CDCl_3$)

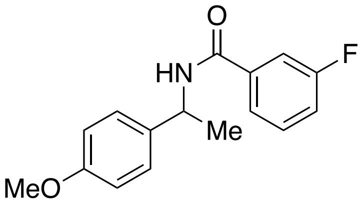

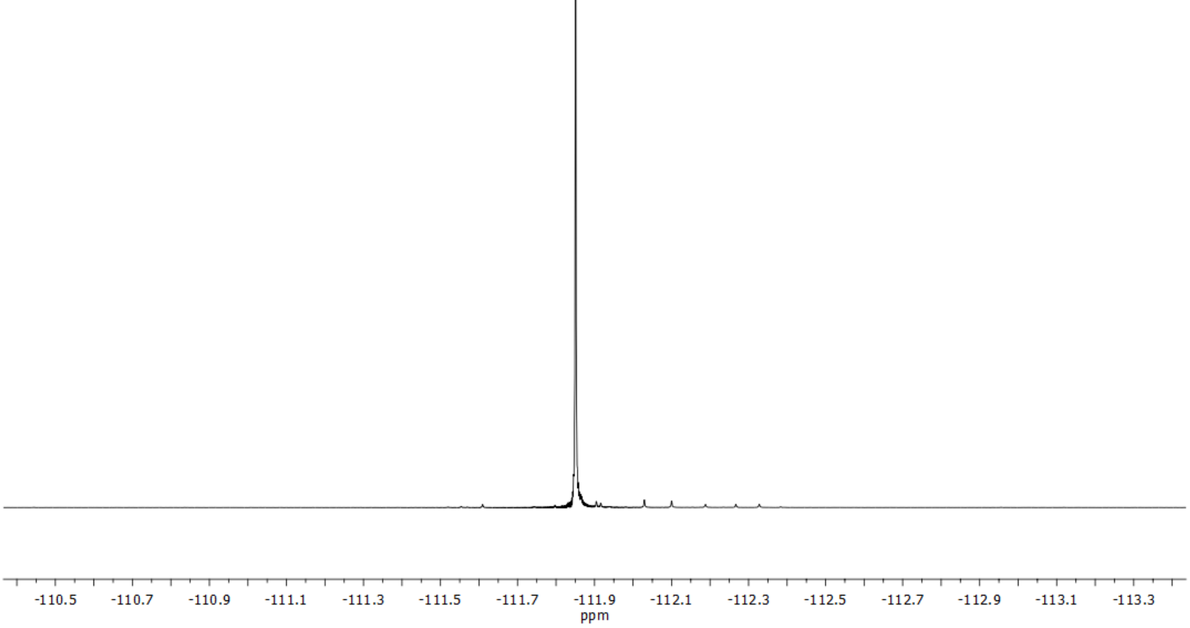

Compound **3m** ^{1}H NMR (400 MHz, $CDCl_3$)

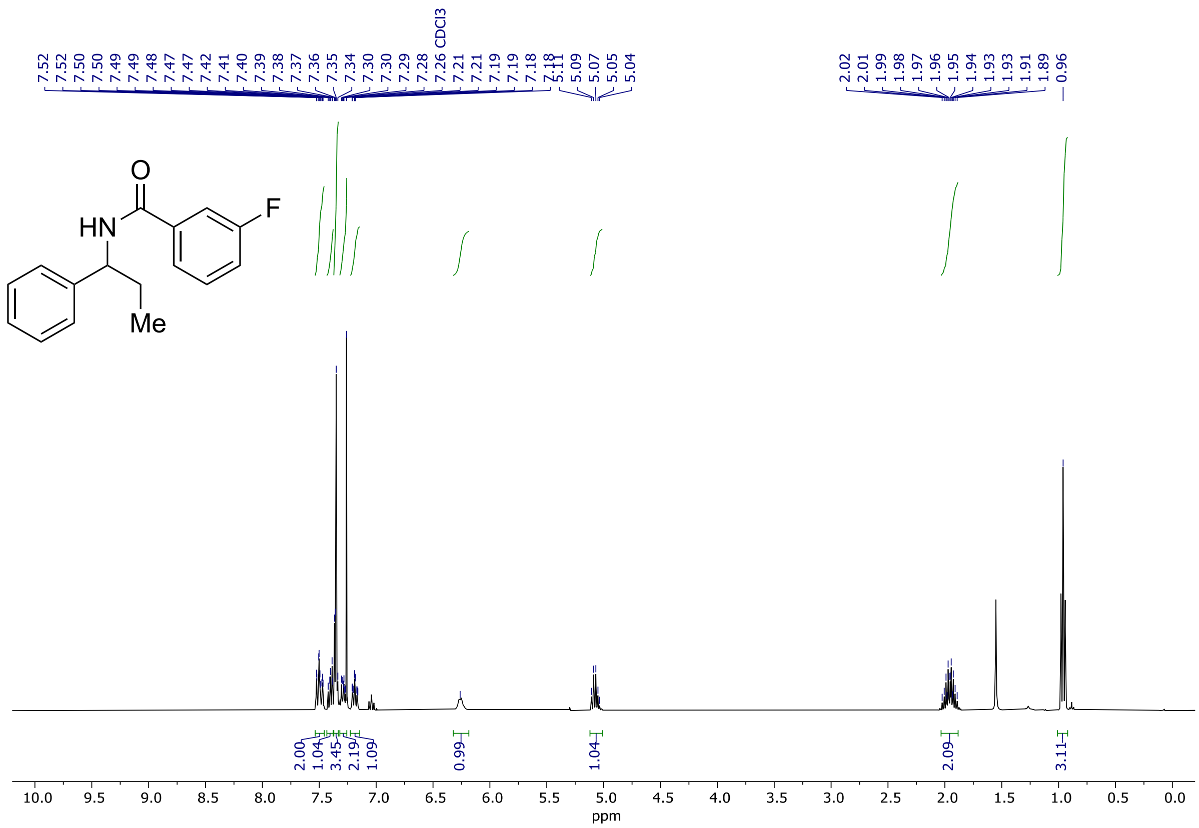

Compound **3m** ^{13}C NMR (101 MHz, $CDCl_3$)

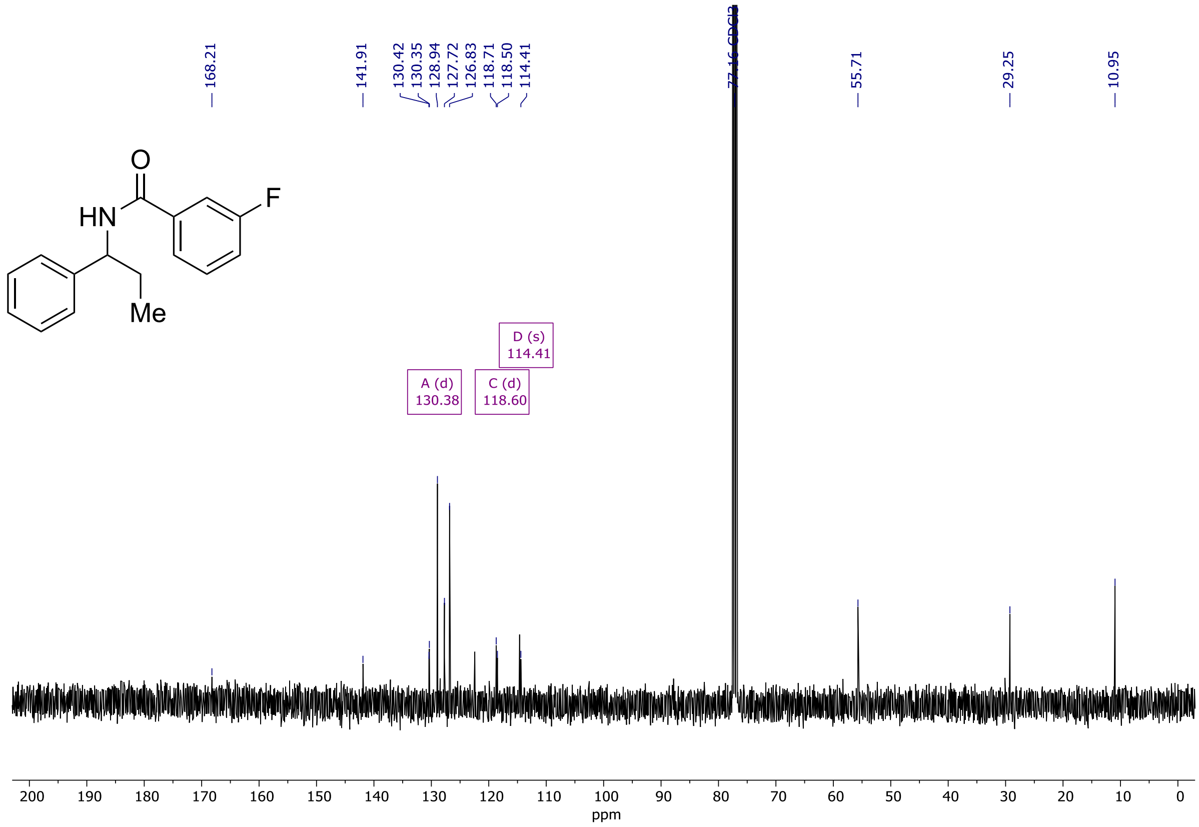

Compound **3m** ^{19}F NMR (377 MHz, $CDCl_3$)

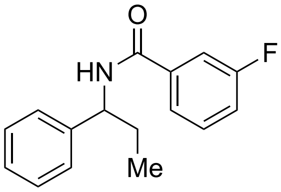

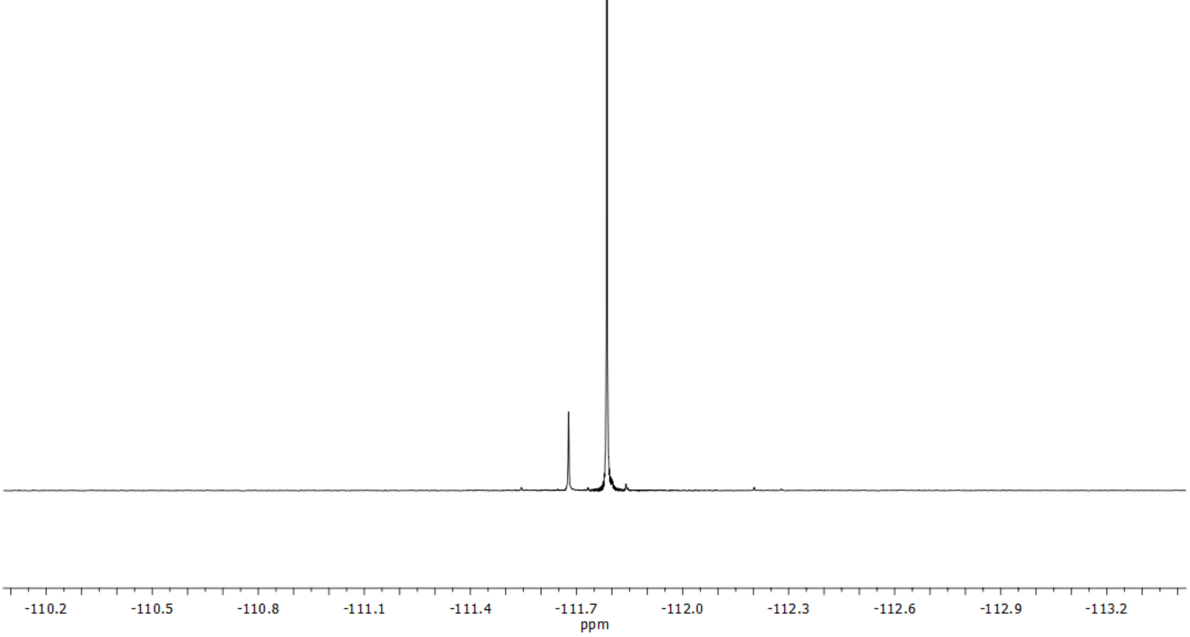

Compound **3n** ^{1}H NMR (400 MHz, $CDCl_3$)

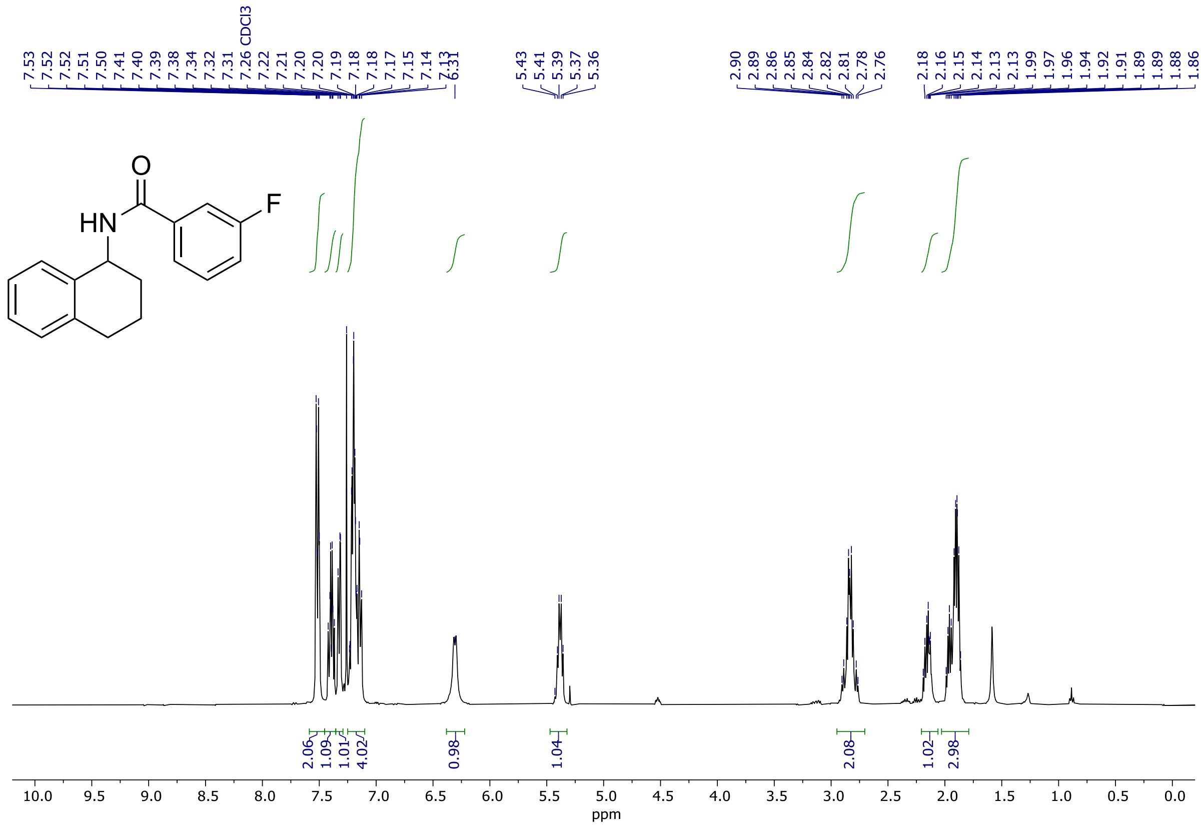

Compound **3n** ^{13}C NMR (101 MHz, $CDCl_3$)

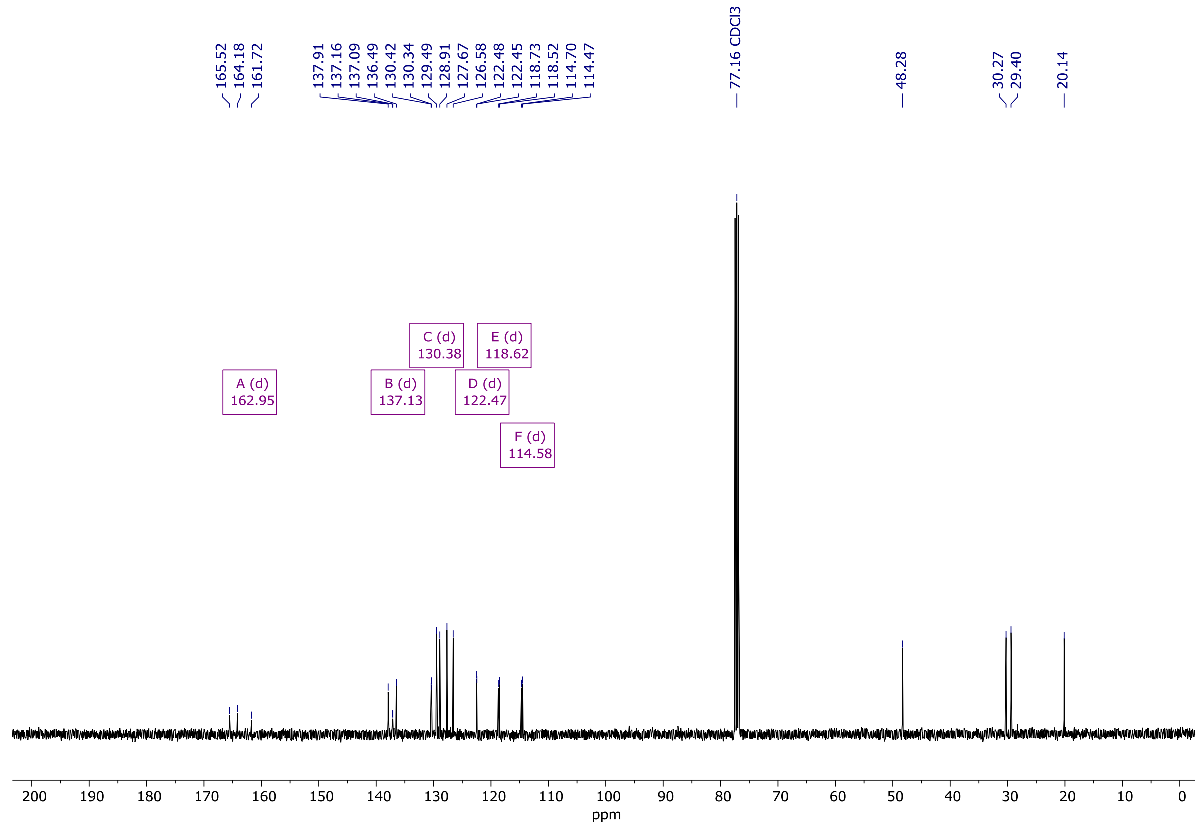

Compound **3n** ^{19}F NMR (377 MHz, $CDCl_3$)

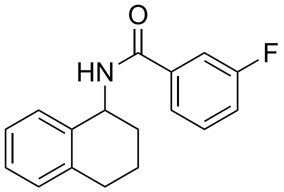

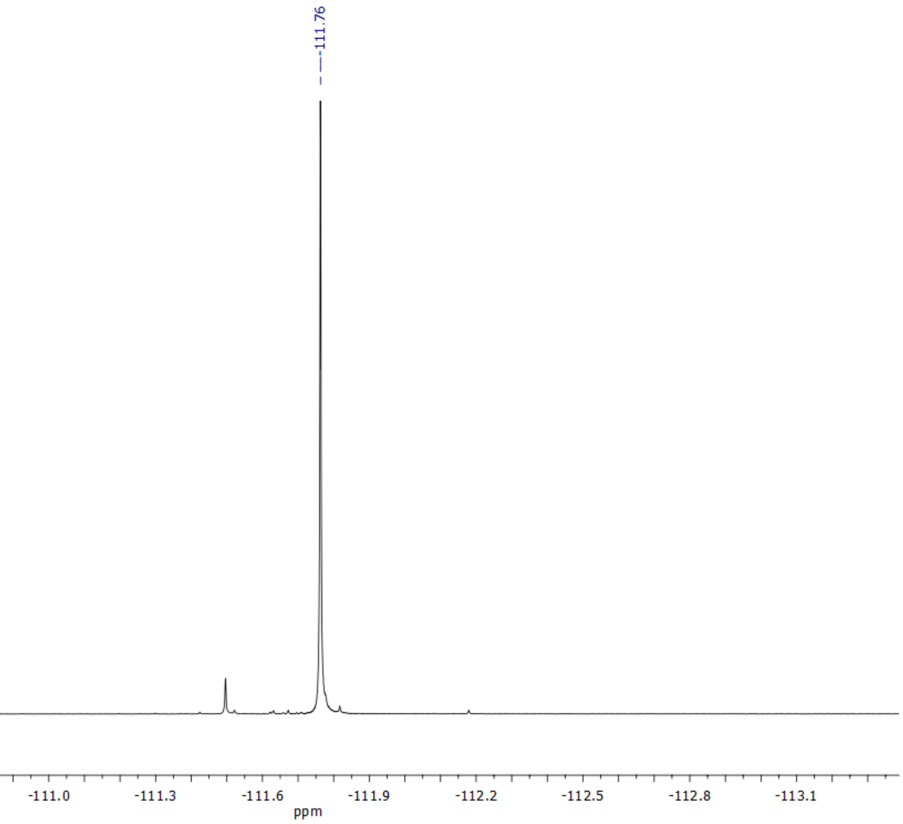

Compound **3o** ^{1}H NMR (400 MHz, $CDCl_3$)

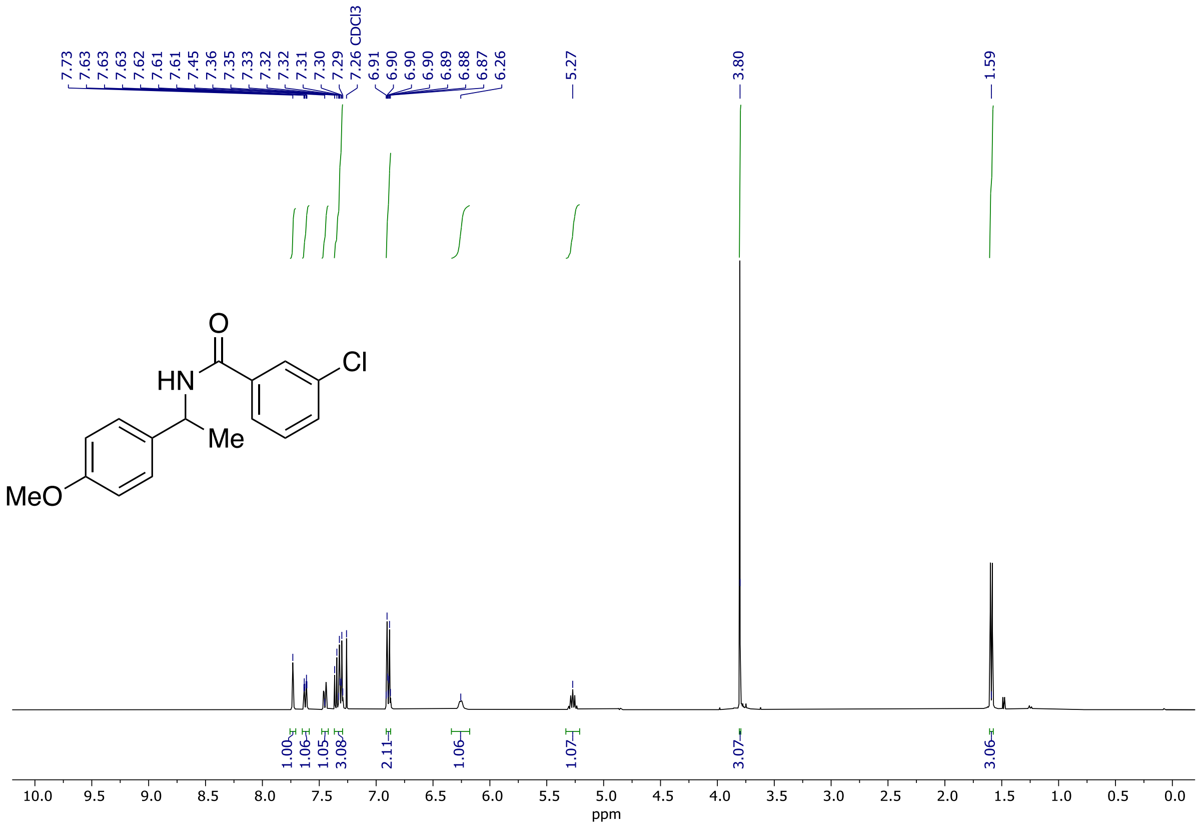

Compound **3o** ^{13}C NMR (101 MHz, $CDCl_3$)

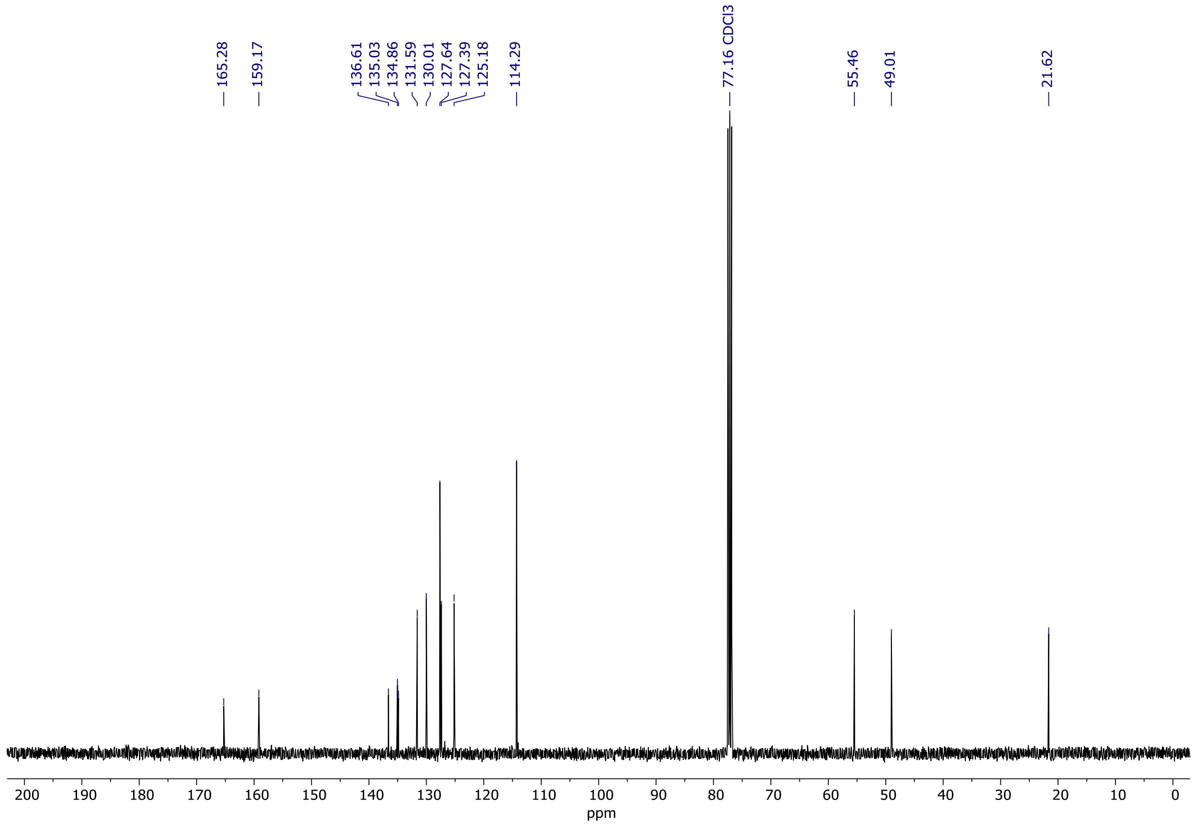

Compound **3p** ^{1}H NMR (400 MHz, $CDCl_3$)

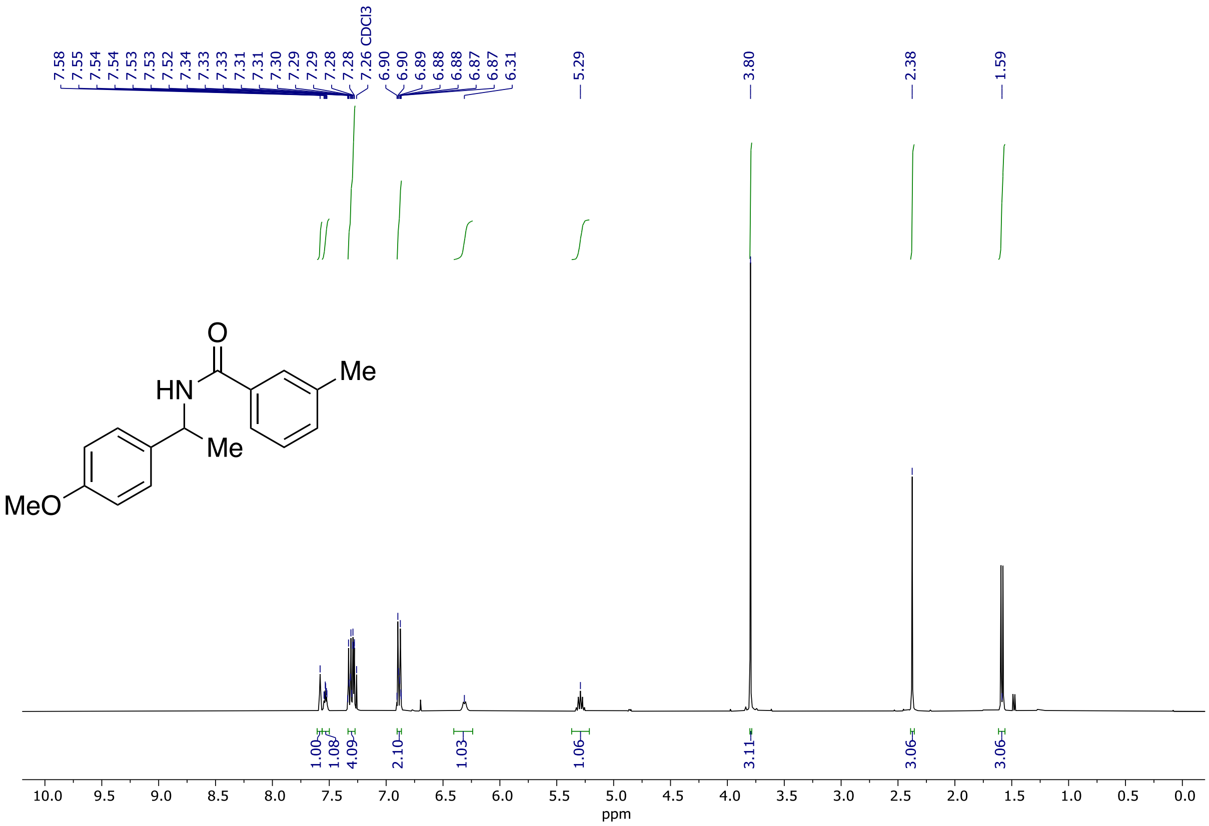

Compound **3p** ^{13}C NMR (101 MHz, $CDCl_3$)

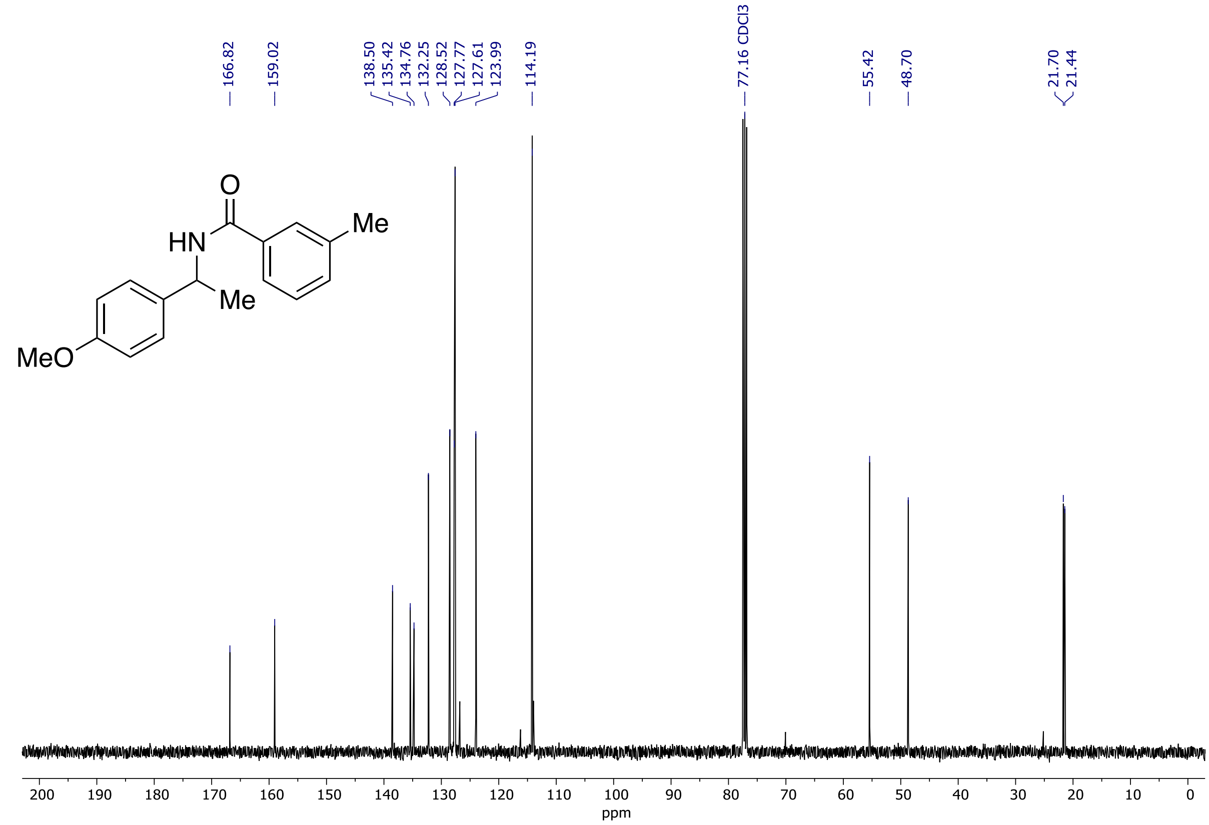

Compound **3q** ^{1}H NMR (400 MHz, $CDCl_3$)

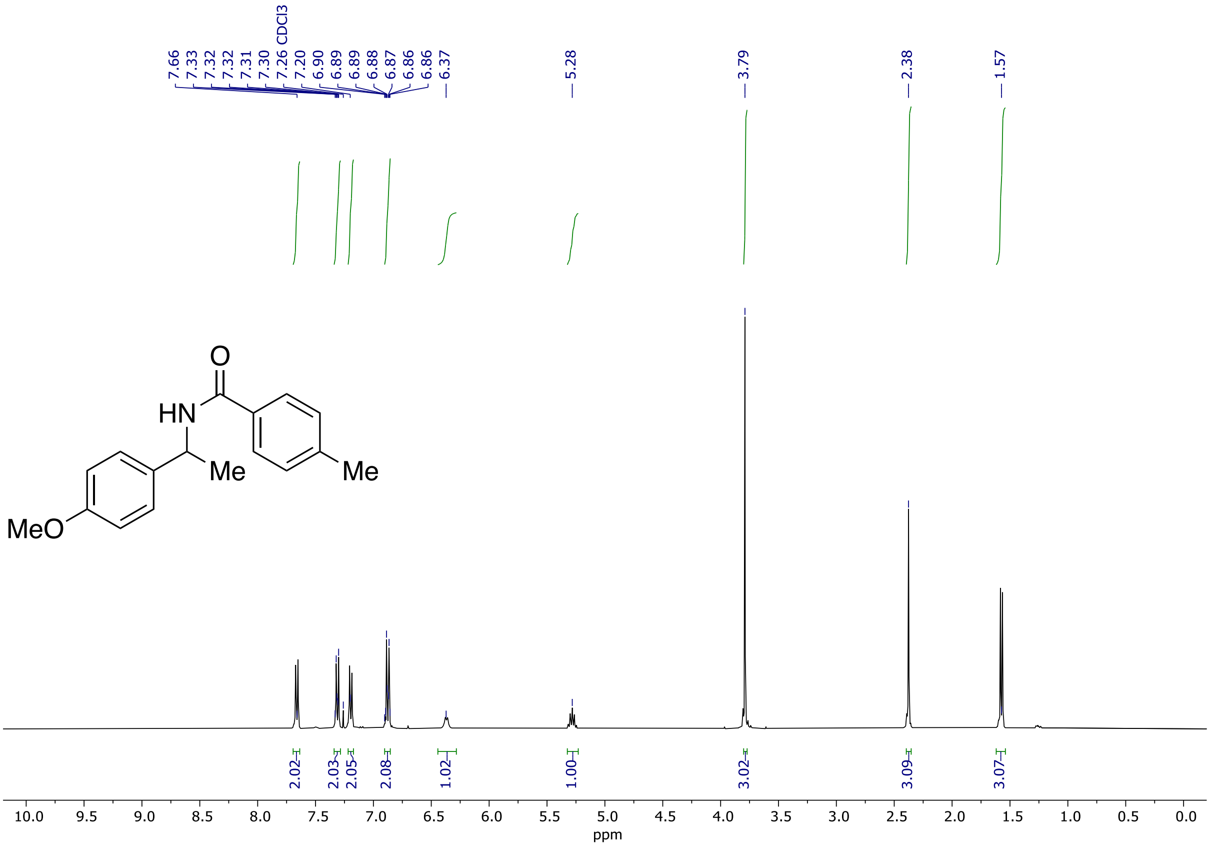

Compound **3q** ^{13}C NMR (101 MHz, $CDCl_3$)

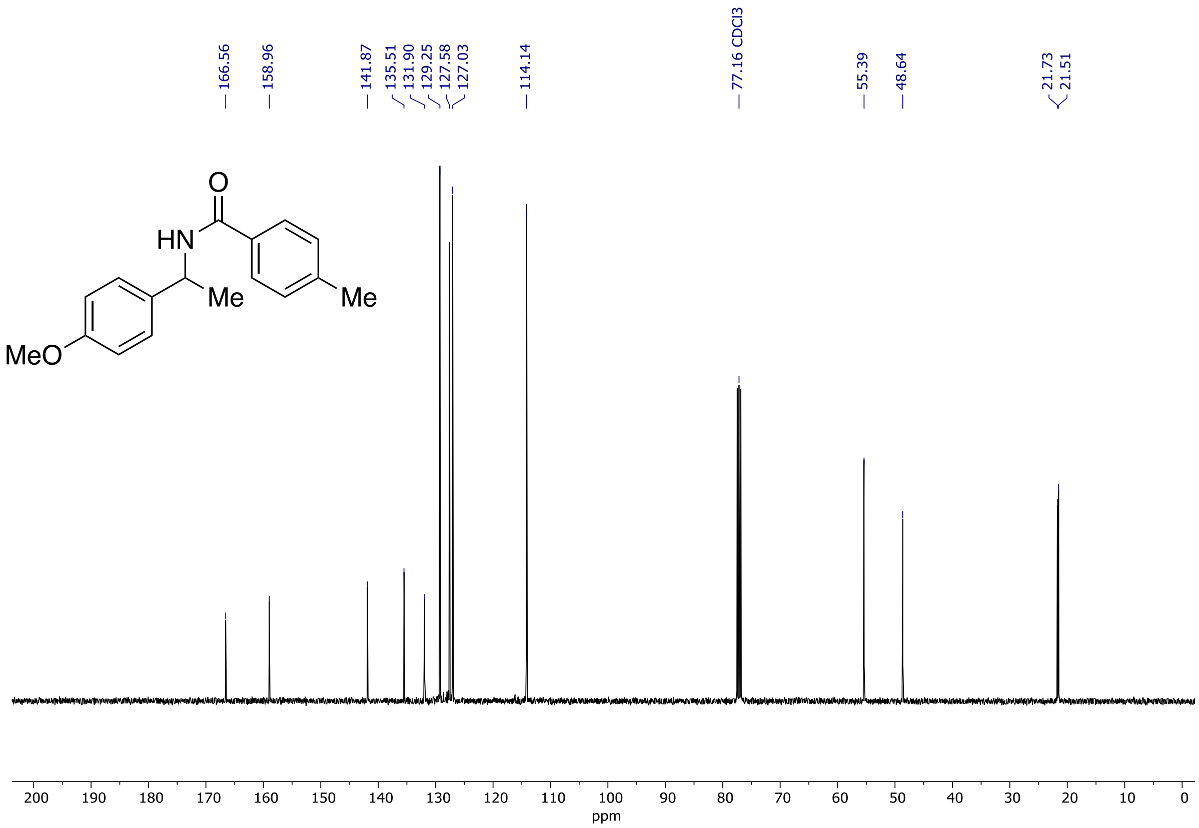

Compound **3r** ^{1}H NMR (400 MHz, $CDCl_3$)

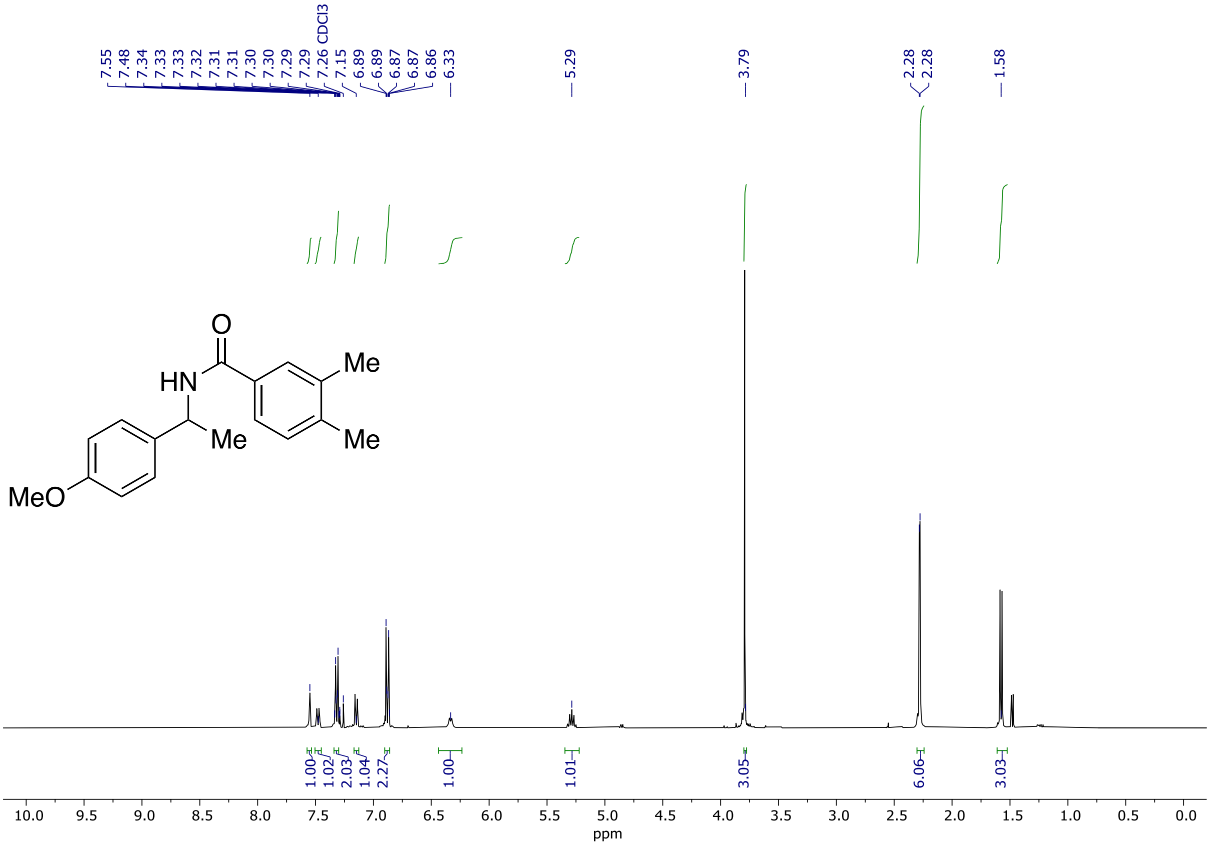

Compound **3r** ^{13}C NMR (101 MHz, $CDCl_3$)

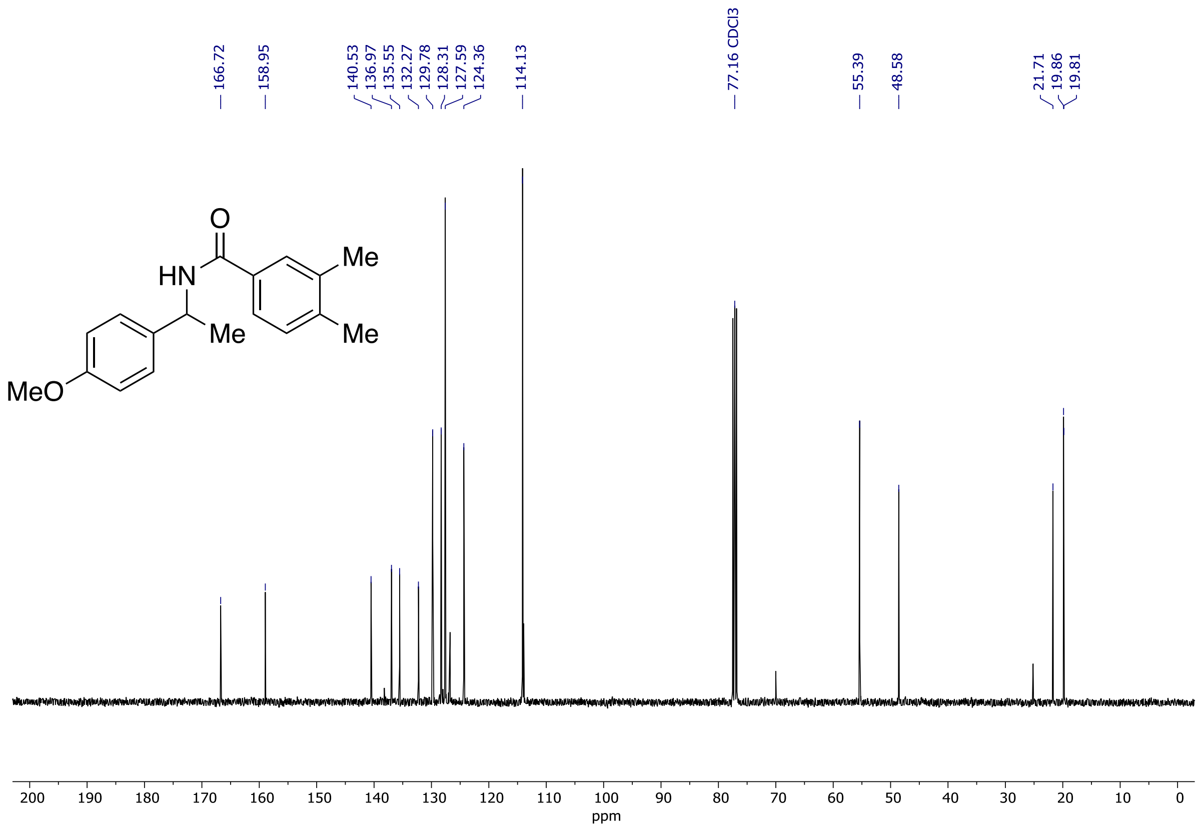

Compound **3s** ^{1}H NMR (400 MHz, $CDCl_3$)

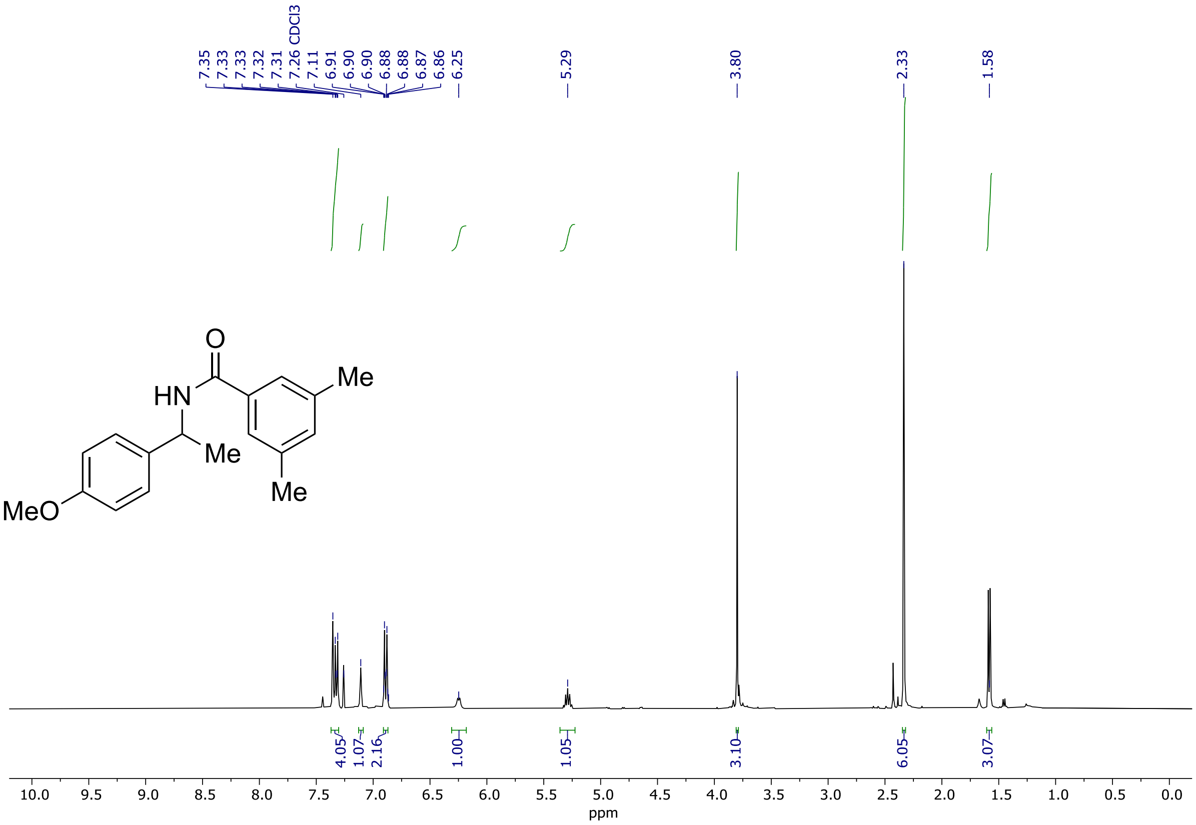

Compound **3s** ^{13}C NMR (101 MHz, $CDCl_3$)

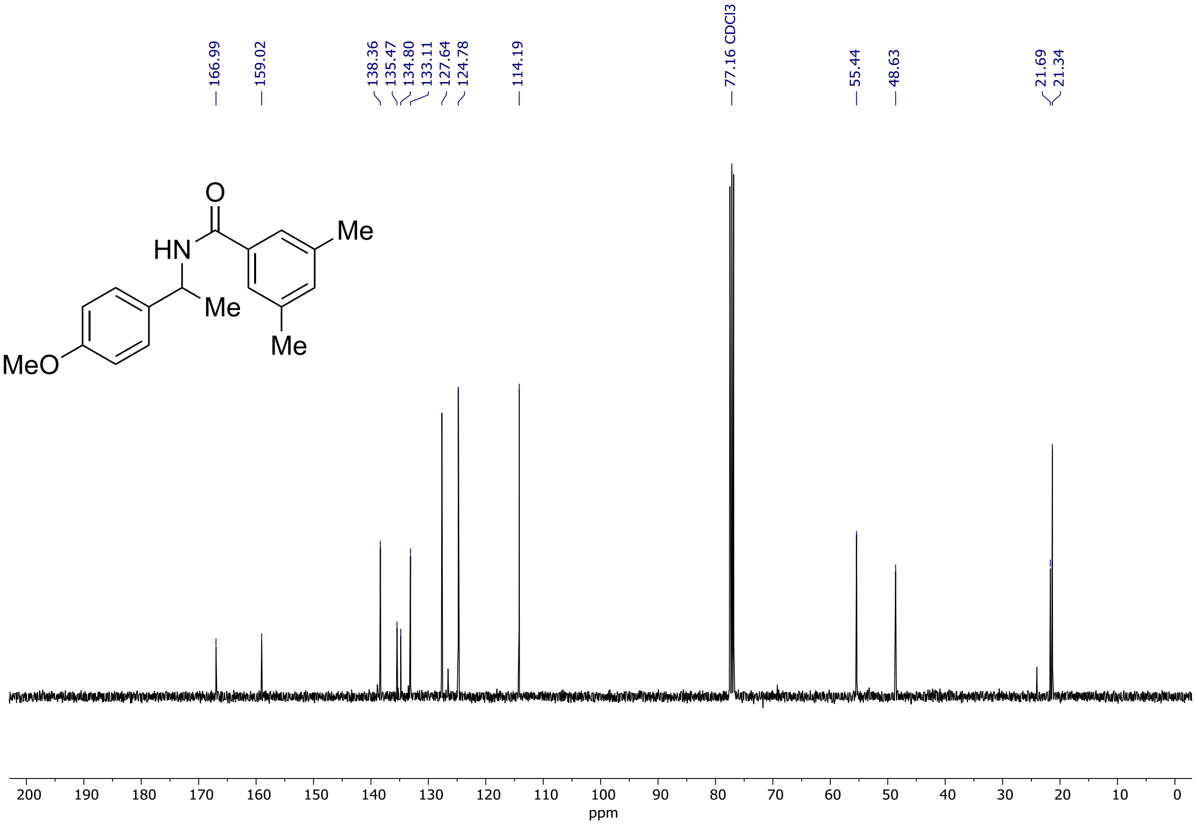

References

(1) Ye, Y.-H.; Zhang, J.; Wang, G.; Chen, S.-Y.; Yu, X.-Q. Cobalt-Catalyzed Benzylic C–H Amination via Dehydrogenative-Coupling Reaction. *Tetrahedron* **2011**, *67*, 4649–4654.

(2) Yasuda, K.; Obora, Y. $NbCl_5$-Mediated Amidation of Olefins with Nitriles to Secondary Amides. *J. Organomet. Chem.* **2015**, *775*, 33–38.

(3) Qian, H.; Widenhoefer, R. A. Platinum-Catalyzed Intermolecular Hydroamination of Vinyl Arenes with Carboxamides. *Org. Lett.* **2005**, *7*, 2635–2638.

(4) Bakhoda, A.; Jiang, Q.; Badiei, Y. M.; Bertke, J. A.; Cundari, T. R.; Warren, T. H. Copper-Catalyzed C(Sp^3)−H Amidation: Sterically Driven Primary and Secondary C−H Site-Selectivity. *Angew. Chem., Int. Ed.* **2019**, *58*, 3421–3425.

(5) Noda, I. Two-Dimensional Infrared Spectroscopy. *J. Am. Chem. Soc.* **1989**, *111*, 8116–8118.
